# Peroxisome-Derived Hydrogen Peroxide Modulates the Sulfenylation Profiles of Key Redox Signaling Proteins in Flp-In T-REx 293 Cells

**DOI:** 10.3389/fcell.2022.888873

**Published:** 2022-04-26

**Authors:** Celien Lismont, Iulia Revenco, Hongli Li, Cláudio F. Costa, Lisa Lenaerts, Mohamed A. F. Hussein, Jonas De Bie, Bernard Knoops, Paul P. Van Veldhoven, Rita Derua, Marc Fransen

**Affiliations:** ^1^ Laboratory of Peroxisome Biology and Intracellular Communication, Department of Cellular and Molecular Medicine, KU Leuven, Leuven, Belgium; ^2^ Laboratory of Protein Phosphorylation and Proteomics, Department of Cellular and Molecular Medicine, KU Leuven, Leuven, Belgium; ^3^ Group of Animal Molecular and Cellular Biology, Institute of Biomolecular Science and Technology (LIBST), Université Catholique de Louvain, Louvain-la-Neuve, Belgium; ^4^ SyBioMa, KU Leuven, Leuven, Belgium

**Keywords:** peroxisome, hydrogen peroxide, cysteine thiol group, YAP1C-based sulfenome mining, peroxiredoxin, mitochondria

## Abstract

The involvement of peroxisomes in cellular hydrogen peroxide (H_2_O_2_) metabolism has been a central theme since their first biochemical characterization by Christian de Duve in 1965. While the role of H_2_O_2_ substantially changed from an exclusively toxic molecule to a signaling messenger, the regulatory role of peroxisomes in these signaling events is still largely underappreciated. This is mainly because the number of known protein targets of peroxisome-derived H_2_O_2_ is rather limited and testing of specific targets is predominantly based on knowledge previously gathered in related fields of research. To gain a broader and more systematic insight into the role of peroxisomes in redox signaling, new approaches are urgently needed. In this study, we have combined a previously developed Flp-In T-REx 293 cell system in which peroxisomal H_2_O_2_ production can be modulated with a yeast AP-1-like-based sulfenome mining strategy to inventory protein thiol targets of peroxisome-derived H_2_O_2_ in different subcellular compartments. By using this approach, we identified more than 400 targets of peroxisome-derived H_2_O_2_ in peroxisomes, the cytosol, and mitochondria. We also observed that the sulfenylation kinetics profiles of key targets belonging to different protein families (e.g., peroxiredoxins, annexins, and tubulins) can vary considerably. In addition, we obtained compelling but indirect evidence that peroxisome-derived H_2_O_2_ may oxidize at least some of its targets (e.g., transcription factors) through a redox relay mechanism. In conclusion, given that sulfenic acids function as key intermediates in H_2_O_2_ signaling, the findings presented in this study provide valuable insight into how peroxisomes may be integrated into the cellular H_2_O_2_ signaling network.

## Introduction

Hydrogen peroxide (H_2_O_2_) has become recognized as one of the major physiological signaling agents (Sies and Jones, 2020). Depending on the cellular context and its local concentration, this oxidant may exhibit antagonistic pleiotropic effects, ranging from cell proliferation, differentiation, and migration to stress adaptations, growth arrest, and even cell death (Lennicke et al., 2015; Sies and Jones, 2020). A major mechanism by which H_2_O_2_ mediates its biological action is through protein thiol oxidation, a process that may trigger changes in protein structure, biochemical activity, subcellular localization, and/or binding affinity. A potential strategy to provide more insight into how temporary changes in local H_2_O_2_ levels can mediate signaling events is to inventory the oxidized proteins and cysteinyl residues involved. However, a key factor for the successful implementation of such an approach is to have access to a robust model system in which H_2_O_2_ production and redox-active cysteine trapping can be strictly controlled in a spatiotemporal manner.

We recently developed a DD-DAO Flp-In T-REx 293 cell line-based approach that allows modulating intracellular H_2_O_2_ production in a subcellular compartment-, dose-, and time-dependent manner (Lismont et al., 2019a). These cells are characterized by the doxycycline (DOX)-inducible expression of destabilization domain (DD)-tagged variants of D-amino acid oxidase (DAO), a peroxisomal flavoprotein that generates H_2_O_2_ while it oxidizes neutral and polar (but not acidic) D-amino acids to their corresponding imino acids. The subcellular localization of DD-DAO can be easily altered by inactivating its C-terminal peroxisomal targeting signal (PTS1) and/or appending other targeting signals. For example, we have generated stable cell lines in which DD-DAO, upon induction by DOX, is localized in the cytosol (c-DD-DAO) or the peroxisome lumen (po-DD-DAO) (Lismont et al., 2019a). Importantly, to stabilize DD-DAO in the cytosol or to allow the efficient post-translational import of this fusion protein into the organelle under study, the cells need to be cultured in the presence of both DOX and Shield1. The latter compound is a small cell-permeable molecule that binds to DD, thereby protecting cytosolic and nuclear DD-containing proteins from proteasomal degradation. To get rid of the not-yet-imported pool of organelle-targeted DD-DAO, which otherwise may complicate the interpretation of the results, the cells can—before the time of analysis—be chased in a culture medium lacking DOX and Shield1. To control the amount and duration of H_2_O_2_ production, varying concentrations of D-amino acids can be added to or withdrawn from the assay medium.

The primary messenger action of H_2_O_2_ depends on its ability to react with deprotonated cysteine residues (Cys-S^-^), a process that in first instance leads to the formation of (unstable) sulfenic acid (-SOH) intermediates (Lismont et al., 2019b). Interestingly, to gain more insight into the H_2_O_2_-dependent sulfenome *in cellulo*, a C-terminal region of yeast AP-1-like transcription factor (YAP1C)-based strategy was developed to trap, visualize, and enrich proteins that are sulfenylated in response to external H_2_O_2_ treatment in *Escherichia coli* (Takanishi et al., 2007) and *Arabidopsis thaliana* (Waszczak et al., 2014). This biological-based approach has several advantages over other more commonly used sulfenome labeling techniques based on (selective) reduction or chemoselective reactivity with sulfenic acids (Kettenhofen and Wood, 2010; Shi and Carroll, 2020). For example, trapping sulfenic acids with YAP1C circumvents signal reduction resulting from the chemical cross-reactivity of sulfenic acids with thiol-capturing electrophiles (e.g., N-ethylmaleimide, iodoacetamide) that are indispensable for most protocols (Reisz et al., 2013). In addition, in contrast to chemical approaches, a genetically encoded probe can be targeted to distinct subcellular locations, thereby reducing sample complexity and providing valuable information about the sulfenylation state of a target protein within different subcellular compartments. Here, we adopted this approach to trap, visualize, and enrich peroxisomal, cytosolic, or mitochondrial proteins that are sulfenylated in human cells in response to peroxisome-derived or externally added H_2_O_2_. Specifically, we employed compartment-specific variants of IBD-SBP-YAP1C, a hybrid protein in which 1) the YAP1C moiety can react with and trap protein sulfenic acids, 2) the SBP domain contains a high-affinity streptavidin-binding peptide that can be used to enrich IBD-SBP-YAP1C complexes on streptavidin matrices, and 3) the IBD domain consists of two streptococcal protein G IgG-binding domains that enable visualization of IBD-SBP-YAP1C complexes in IgG overlay assays.

Peroxisomal respiration may be responsible for up to 20% of total oxygen consumption and 35% of total H_2_O_2_ production, at least in some mammalian tissues such as liver (De Duve and Baudhuin, 1966; Boveris et al., 1972). In addition, disturbances in peroxisomal H_2_O_2_ metabolism have been associated with aging and age-associated disease (Fransen and Lismont, 2019). Despite this, very little is known about how peroxisomes are embedded in H_2_O_2_-mediated signaling networks. In this study, we were able for the first time to map the potential impact of peroxisome-derived H_2_O_2_ on cellular redox signaling networks. This opens new perspectives for research on how perturbations in peroxisomal H_2_O_2_ metabolism may contribute to the initiation and development of oxidative stress-related diseases.

## Materials and Methods

### Plasmids

The cDNA coding for a human codon-optimized variant of IBD-SBP-YAP1C (Supplementary Figure S1) was synthesized by Integrated DNA Technologies and provided in the pUCIDT (Kan) cloning vector (pMF1986). A mammalian expression plasmid encoding IBD-SBP-YAP1C (pMF1987) was generated by transferring the EcoRI/NotI-restricted fragment of pMF1986 into the EcoRI/NotI-restricted backbone fragment of pMF1839 (Walton et al., 2017). Mammalian expression vectors encoding mitochondrial (pMF1991), peroxisomal (pMF1992), or cytosolic (pMF2029) variants of IBD-SBP-YAP1C were generated by amplifying the IBD-SBP-YAP1C template via PCR (forward oligo: 5′-ggg​gga​tcc​cat​ggc​atc​aat​gca​gaa​gct​g-3′; reverse oligos: 5′-ccg​ggg​gcg​gcc​gct​cag​ttc​ata​tgt​t-3′ (for pMF1991 and pMF2029) and 5′-ccg​ggg​gcg​gcc​gct​caa​agc​tta​ctt​ttg​ttc​ata​tgt​tta​ttc​aat​gca-3′ (for pMF1992)) and subcloning the BamHI/NotI-restricted PCR products into the BamHI/NotI-restricted backbone fragments of pKillerRed-dMito (Evrogen) (for pMF1991) or pEGFP-N1 (Clontech) (for pMF1992 and pMF2029). Plasmid sequences were validated by DNA sequencing (LGC Genomics). The plasmids encoding EGFP-HsPEX11B (pTW110) (Fransen et al., 2001), c-roGFP2 (pMF1707) (Ivashchenko et al., 2011), po-roGFP2 (pMF1706) (Ivashchenko et al., 2011), or mt-roGFP2 (pMF1762) (Ivashchenko et al., 2011) have been described elsewhere. The plasmid encoding EGFP-HSPB1 was kindly provided by Prof. Dr. Ludo Van Den Bosch (KU Leuven, Belgium).

### Cell Culture and Transfections

Cell culture was essentially performed as previously described (Lismont et al., 2019a). Briefly, all cells were cultured at 37°C in a humidified 5% CO_2_ incubator in minimum essential medium Eagle α (Lonza, BE12-169F) supplemented with 10% (v/v) fetal bovine serum (Biowest, S181B), 2 mM UltraGlutamine I (Lonza, BE17-605E/U1), and 0.2% (v/v) MycoZap (Lonza, VZA-2012). Transfections were performed by using the Neon Transfection System (Thermo Fisher Scientific; 1,150 V, 20-ms pulse width, two pulses).

### Generation and Manipulation of DD-DAO/IBD-SBP-YAP1C Flp-In T-REx 293 Cell Lines

The Flp-In T-REx 293 cell lines expressing DD-DAO in peroxisomes or the cytosol have been detailed elsewhere (Lismont et al., 2019a). To generate po- or c-DD-DAO Flp-In T-REx 293 cell lines constitutively expressing c-IBD-SBP-YAP1C, po-IBD-SBP-YAP1C, or mt-IBD-SBP-YAP1C, the corresponding Flp-In T-REx 293 cells were transfected with pMF2029, pMF1992, or pMF1991, respectively. Starting from 2 days later, the cells were routinely cultured in a medium supplemented with 1) 10 μg/ml blasticidin (InvivoGen; ant-bl) and 100 μg/ml hygromycin B Gold (InvivoGen, ant-hg) to maintain the properties of the Flp-In T-REx 293 cell lines stably expressing peroxisomal or cytosolic DD-DAO, and 2) 200 μg/ml of G418 (Acros Organics, BP673-5) to select for cells carrying the neomycin resistance cassette (with a minimum period of 3 weeks). To modulate the expression levels of DD-DAO in these cells, they were incubated for 3 or 4 days in the absence or presence of 1 μg/ml doxycycline (DOX) (Sigma, D9891) and 500 nM Shield1 (Clontech, 632,189). Treatments were always followed by a 24-h chase period (no DOX, no Shield1) in order to remove the pool of residual cytosolic po-DD-DAO (Lismont et al., 2019a), unless specified otherwise.

### Fluorescence Microscopy

Fluorescence microscopy was carried out as described previously (Ramazani et al., 2021). The following excitation filters (Ex), dichromatic mirrors (Dm), and emission filters (Em) were chosen to match the fluorescent probe specifications: DAPI (Ex: BP360-370; Dm: 400 nm cut-off; Em: BA420-460); EGFP (Ex: BP470-495; Dm: 505 nm cut-off; Em: BA510-550); and Texas Red (Ex: BP545-580; Dm: 600 nm cut-off; Em: BA610IF). The cellSens software (Olympus Belgium) was used for image analysis. Samples for immunofluorescence microscopy were fixed, counterstained with 0.5 μg/ml DAPI (Sigma, D-9542) in Dulbecco’s phosphate-buffered saline (DPBS) for 1 min, and processed as described (Passmore et al., 2020).

### Redox Proteomics Sample Preparation and Analysis

Cells were grown to 60–80% confluency, trypsinized, collected in cell culture medium, pelleted (150 x g, 5 min), and washed once with DPBS without calcium and magnesium (BioWest, L0615). Cell density was determined by Bürker chamber counting and adjusted to 10^6^ cells/ml. After being subjected to different treatments (for specifications, see Results section), N-ethylmaleimide (NEM) (TCI, E0136) dissolved in methanol (Fisher Scientific, M/4062/17) was added to a final concentration of 10 mM. Next, the cells were pelleted (150 x g, 5 min), resuspended in lysis buffer (50 mM Tris-HCl pH 7.5, 150 mM NaCl, 1% (v/v) Triton X-100, 10% (v/v) glycerol) containing 10 mM NEM and a protease inhibitor mix (Sigma-Aldrich, P2714)) at a density of 10^7^ cells/ml, and lysed on ice for 10 min. Thereafter, a cleared lysate was produced by double centrifugation (20,000 x g, 10 min), each time discarding the pellet.

The cleared lysates were mixed with 300 µl of (prewashed) high capacity streptavidin agarose beads (Thermo Scientific, 20359) and incubated at 4°C on a rotation mixer to enrich IBD-SBP-YAP1C complexes. After 2 h, the bead suspensions were transferred to Micro-Spin columns (Thermo Scientific, 89879) and consecutively washed five times with lysis buffer, five times with wash buffer (50 mM Tris-HCl pH 7.5, 150 mM NaCl, 1% (v/v) Triton X-100), and five times with 50 mM Tris-HCl pH 8.0. Finally, the disulfide-bonded interaction partners of IBD-SBP-YAP1C were eluted by incubating the columns three times for 15 min with 200 µl of elution buffer (10 mM DTT in 50 mM Tris-HCl pH 8.0). The eluates were pooled and subsequently processed for proteomics analysis. At each step, small aliquots were saved for immunoblot analysis. To analyze comparable amounts of IBD-SBP-YAP1C-containing protein complexes, the ratio of cleared lysate to streptavidin agarose beads was chosen such that the affinity matrix was slightly oversaturated, as determined by detection of residual IBD-SBP-YAP1C in the non-bound fraction.

Eluates were incubated for 30 min at 37°C to allow the DTT to fully reduce all proteins. Thereafter, the samples were alkylated (37°C, 30 min) with 25 mM iodoacetamide (Sigma, I-6125). Excess iodoacetamide was quenched with 25 mM DTT (AppliChem, A2948) (37°C, 30 min). Subsequently, the proteins were precipitated as described (Wessel and Flügge, 1984) and digested overnight with modified trypsin (Pierce, 90057) in the presence of 50 mM ammonium bicarbonate (Sigma-Aldrich, A6141), 5% acetonitrile (Applied Biosystems, 400315), and 0.01% ProteaseMAX surfactant (Promega, V2072). Trypsin was inactivated by addition of 0.5% (v/v) trifluoroacetic acid (Applied Biosystems, 400028). The resulting peptides were desalted with C18 ZipTip pipette tips (Merck Millipore, ZTC18S960) and loaded onto an Ultimate 3000 UPLC system (Dionex, Thermo Fisher Scientific) equipped with an Acclaim PepMap100 pre-column (C18; particle size: 3 μm; pore size 100 Å; diameter: 75 μm; length: 20 mm; Thermo Fisher Scientific) and a C18 PepMap analytical column (particle size: 2 μm; pore size: 100 Å; diameter: 50 μm; length: 150 mm; Thermo Fisher Scientific) using a 40 min linear gradient (300 nl/min) coupled to a Q Exactive Orbitrap mass spectrometer (Thermo Fisher Scientific) operated in data-dependent acquisition mode. After the initial pilot experiment, the mass spectrometry (MS) method was adapted, essentially doubling the maximum injection time for MS/MS. Peptides were identified by Mascot (Matrix Science) using Uniprot *Homo sapiens* as a database (# entries: 194619). S-carbamidomethylation (C), N-ethylmaleimide (C), and oxidation (M) were included as variable modifications. Two missed cleavages were allowed, peptide tolerance was set at 10 ppm and 20 mmu for MS and MS/MS, respectively.

Progenesis QI software (Nonlinear Dynamics) was used for the relative quantification of proteins based on peptides validated by the Proteome Discoverer 2.2 Percolator node. Only exclusive peptides having a peptide spectral match (PSM) with a posterior error probability (PEP) smaller than 0.001 (10^–3^) in at least one of the conditions of the experiment were taken into account for quantification (Käll et al., 2008). Proteins that could not be unambiguously identified or were identified as keratins, extracellular proteins, or proteins that—according to the Human Protein Atlas database (http://www.proteinatlas.org)—are not expressed in HEK-293 cells, were manually removed. Proteins enriched at least 2.5-fold upon H_2_O_2_ exposure were retained as H_2_O_2_ targets. For every experiment, data derived from one biological replicate are shown.

### Antibodies

The pre-immune serum was collected from a rabbit before immunization with the 21 kDa subunit of rat palmitoyl-CoA oxidase (Baumgart et al., 1996); the rabbit polyclonal antisera against EGFP (Fransen et al., 2001), PEX13 (Fransen et al., 2001), PRDX1 (Goemaere and Knoops, 2012), PRDX3 (Goemaere and Knoops, 2012), or PRDX5 (Goemaere and Knoops, 2012) have been described elsewhere; and the rabbit polyclonal antisera against TUBA (Santa Cruz Biotechnology, sc-5546) and the goat anti-rabbit secondary antibodies, conjugated to Texas Red (Calbiochem, 401355) or alkaline phosphatase (Sigma, A3687), were commercially obtained.

### Mobility Shift Electrophoresis

Electrophoretic mobility shift assays (EMSAs) were performed as described previously (Lismont et al., 2019a). A slightly modified protocol was used for samples taken during the proteomics sample preparation. Specifically, for the “input” and “non-bound” samples, 50 µl of cleared cell lysates and bead supernatants were respectively mixed with 2X SDS-PAGE sample buffer without reducing agent and heated to 65°C for 10 min. For the “bound” and “bound after elution” samples, 10 µl of bead volume was mixed with non-reducing 2X SDS-PAGE sample buffer and heated to 100°C for 10 min.

### Other

Subcellular fractionations of rat liver (Anthonio et al., 2009) and HEK-293 cells (Lismont et al., 2019c) were carried out as described elsewhere. The animal and human cell-related studies were reviewed and approved by the local UZ/KU Leuven ethics Committee (approval numbers: P092/2018, S63097, and S62366). Functional enrichment analysis was performed using g:GOST (https://biit.cs.ut.ee/gprofiler/gost) within the g:Profiler tool (Raudvere et al., 2019). Heat maps were generated with Graphpad Prism version 9.0.0 for Windows (GraphPad Software). The iCysMod (http://icysmod.omicsbio.info/index.php) (Wang et al., 2021) and TF2DNA (http://fiserlab.org/tf2dna_db//index.html) (Pujato et al., 2014) databases were used as resources to search for known protein cysteine oxidation sites and transcription factors in the human dataset, respectively.

## Results

### Validation of the Po-DD-DAO/IBD-SBP-YAP1C Flp-In T-REx 293 Cell Lines

**FIGURE 1:**
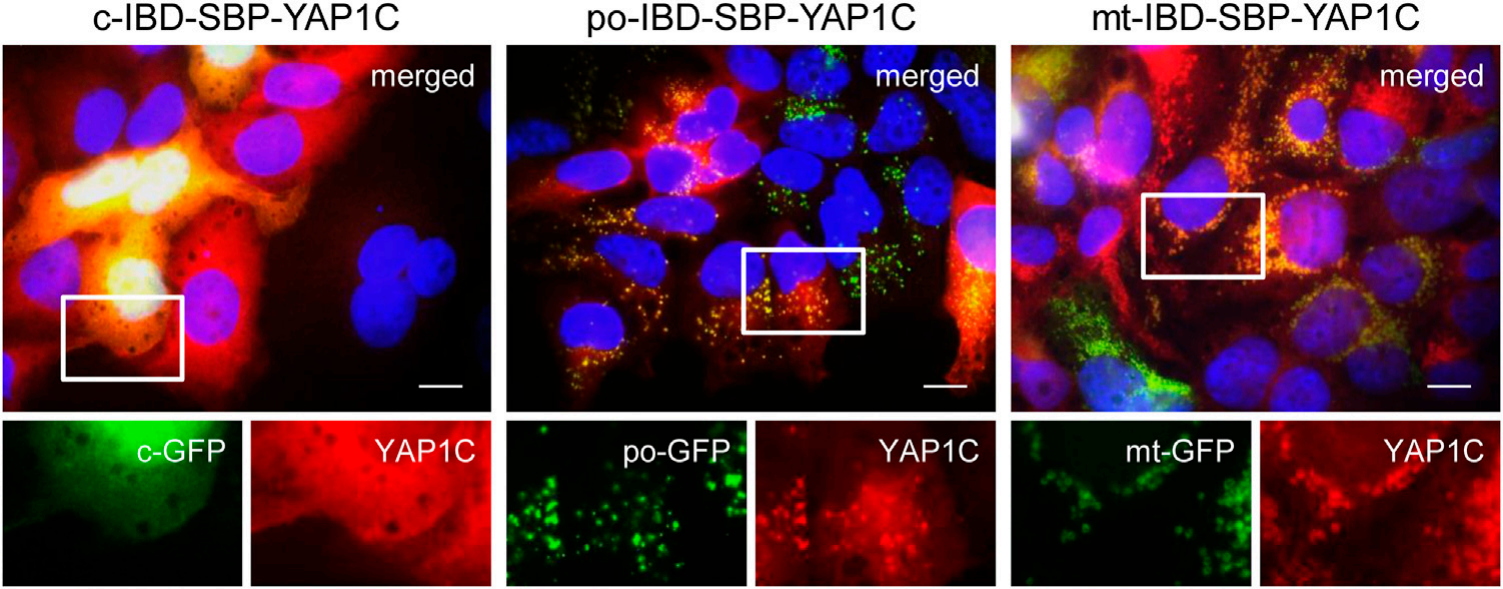
Validation of the subcellular localization of different IBD-SBP-YAP1C fusion proteins in po-DD-DAO Flp-In T-REx 293 cells. Po-DD-DAO Flp-In T-REx 293 cells enriched for expression of c-, po-, or mt-IBD-SBP-YAP1C were transfected with plasmids encoding c-, po-, or mt-roGFP2 (GFP) as marker for the respective cell compartment. After 3 days, the cells were processed for immunofluorescence microscopy using rabbit pre-immune serum and goat anti-rabbit secondary antibody conjugated to Texas Red. Nuclei were counterstained with DAPI. Scale bars, 10 µm. Representative images are shown. The boxed areas in the upper panels are enlarged in the lower panels.

To confirm the correct localization of the different IBD-SBP-YAP1C fusion proteins in the G418-enriched po-DD-DAO Flp-In T-REx 293 cell lines, we co-expressed compartment-specific fluorescent marker proteins (Figure 1). From this experiment, it is clear that the majority of the cells express IBD-SBP-YAP1C. However, it is also evident that, at least in some cells, varying portions of po-IBD-SBP-YAP1C and, to a certain extent, also mt-IBD-SBP-YAP1C still reside in the cytosol. Given that 1) the functionality of peroxisomal and mitochondrial targeting signals fused to a heterologous protein strongly depends on the protein context (Yogev and Pines, 2011; Kunze, 2018), 2) the priority of protein import into mitochondria and peroxisomes is governed by competition for binding to limiting amounts of import receptor (Weidberg and Amon, 2018; Rosenthal et al., 2020), and 3) expression of the YAP1C fusion proteins is driven by the cytomegalovirus promoter, one of the strongest naturally occurring promoters (Even et al., 2016), this observation may not be that surprising. Although this may complicate the interpretation of downstream results, this knowledge also allows us to correctly anticipate this shortcoming.

### Differentially-Localized IBD-SBP-YAP1C Proteins Form Different Protein Complexes Upon Exposure of Cells to Exogenous or Peroxisome-Derived H_2_O_2_

**FIGURE 2:**
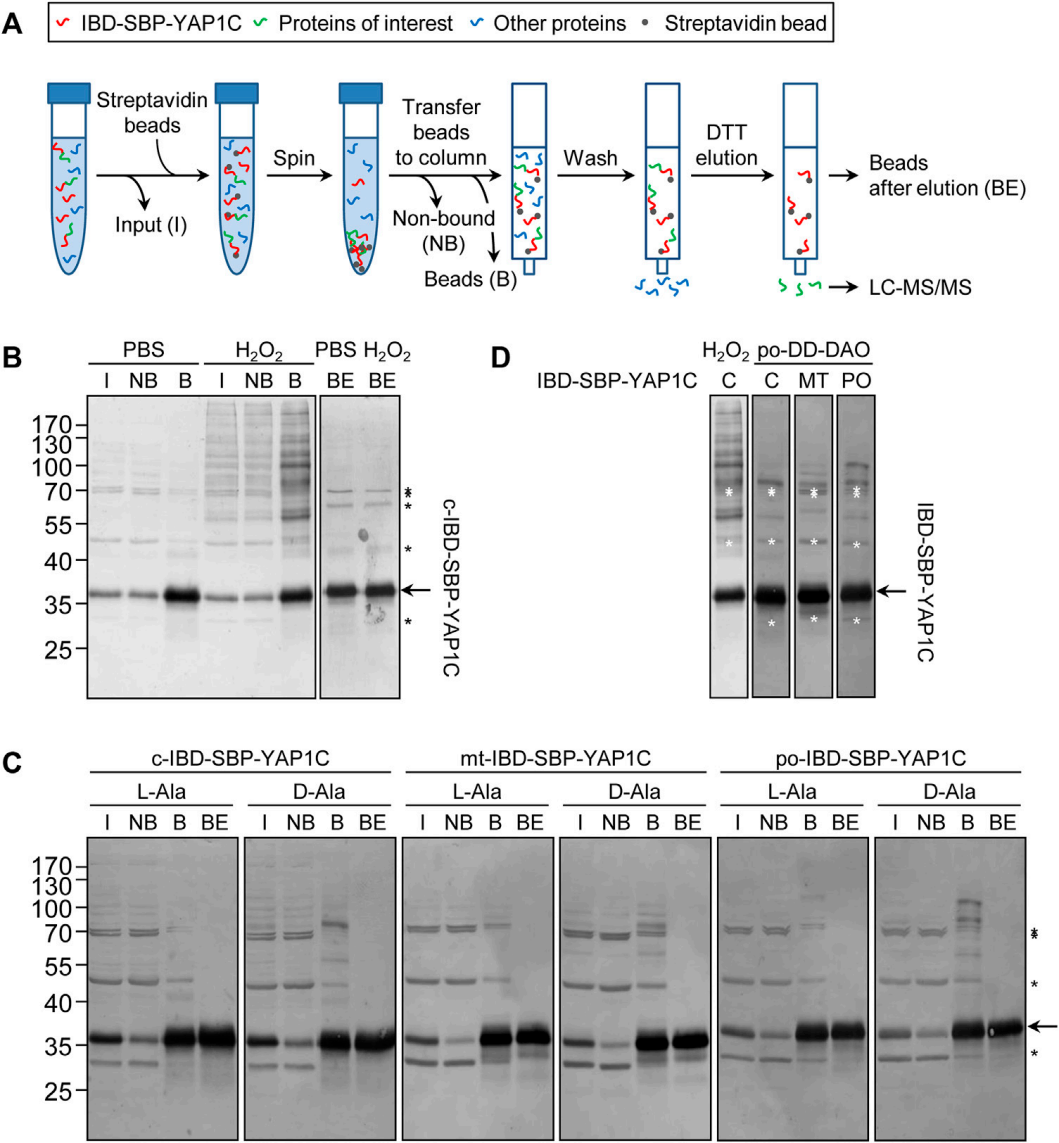
The interaction profiles of IBD-SBP-YAP1C with sulfenylated proteins differ considerably depending on the source of H_2_O_2_ as well as its subcellular location. **(A)** Outline of the experimental workflow (for a detailed explanation, see Materials and Methods section). I, input; NB, non-bound proteins; B, bound IBD-SBP-YAP1C complexes; BE, beads after elution. **(B)** C-IBD-SBP-YAP1C complex formation upon external H_2_O_2_ treatment. Flp-In T-REx 293 cells expressing c-IBD-SBP-YAP1C were incubated in DPBS containing 10 mM 3-AT and supplemented or not with 1 mM H_2_O_2_. After 10 min, free thiol groups were blocked with NEM, and the samples were processed as depicted in panel A and subsequently subjected to immunoblotting with rabbit pre-immune serum (see Materials and Methods section). **(C)** Peroxisomal H_2_O_2_ production triggers IBD-SBP-YAP1C complex formation in the cytosol, mitochondria, and peroxisomes. After induction and chase, po-DD-DAO Flp-In T-REx 293 cells expressing c-, mt-, or po-IBD-SBP-YAP1C were incubated in DPBS containing 10 mM 3-AT and supplemented with either 10 mM L- or D-Ala. After 10 min, the cells were processed as detailed in the legend of panel **(B)**. **(D)** Comparison of IBD-SBP-YAP1C complexes formed under different experimental conditions. In the two left lanes, the staining patterns of c-IBD-SBP-YAP1C formed upon external H_2_O_2_ addition or peroxisomal H_2_O_2_ production are compared; in the three right lanes, differentially located IBD-SBP-YAP1C complexes formed upon peroxisomal H_2_O_2_ production are compared. The different lanes are the aligned bound samples of those shown in panels **(B)** and **(C)**. The arrows and asterisks mark IBD-SBP-YAP1C and non-specific immunoreactive bands, respectively.

To validate the *in cellulo* trapping strategy for sulfenylated proteins, we first investigated IBD-SBP-YAP1C complex formation in different subcellular compartments upon exposure of cells to exogenous or peroxisome-derived H_2_O_2_. An outline of the experimental workflow is depicted in Figure 2A and detailed in the Materials and Methods section. Importantly, given that inhibition of catalase activity with 3-amino-1,2,4-triazole (3-AT) increases the responsiveness of Flp-In T-REx 293 cells to peroxisome-derived H_2_O_2_ (Lismont et al., 2019a), we routinely added this inhibitor to the assay medium. In a first experiment, Flp-In T-REx 293 cells expressing c-IBD-SBP-YAP1C were exposed or not to 1 mM H_2_O_2_ for 10 min and subsequently processed as detailed in the legend to Figure 2A. IgG blot overlay analysis of the input fractions (I), the non-bound fractions (NB), the streptavidin-bound fractions (B), and the streptavidin-bound fractions after elution with a reducing agent (BE) confirmed that exposure of the cells to external H_2_O_2_ triggered the formation of many c-IBD-SBP-YAP1C-containing higher molecular weight complexes that can be enriched on streptavidin beads and are sensitive to the reducing agent dithiothreitol (DTT) (Figure 2B). The latter feature is important to allow selective elution of target proteins from the affinity matrix. Next, a similar experiment was performed with Flp-In T-REx 293 cells expressing po-DD-DAO and a compartment-specific variant of IBD-SBP-YAP1C and in which peroxisomal H_2_O_2_ production was induced or not by supplementing the assay medium (DPBS) with 10 mM D- or L-Ala, respectively. Multiple IBD-SBP-YAP1C-containing protein complexes could be detected in all conditions in which peroxisomal H_2_O_2_ was produced (Figure 2C). Interestingly, a direct comparison of the staining patterns of the streptavidin-enriched fractions clearly shows that the interaction profiles of IBD-SBP-YAP1C with sulfenylated proteins differed considerably depending on the source of H_2_O_2_ as well as on the subcellular location of the YAP1C fusion protein (Figure 2D).

### The Subcellular Sulfenome Upon Exogenous H_2_O_2_ Treatment: A Pilot Experiment

To corroborate the sulfenome mining strategy, we carried out an exploratory experiment in which cells expressing c-, mt-, or po-IBD-SBP-YAP1C were treated or not with 1 mM H_2_O_2_ for 10 min. After documenting IBD-SBP-YAP1C complex formation (Supplementary Figure S2), the DTT eluates were processed for LC-MS/MS analysis. After validation of the identified peptides, probable contaminating proteins (e.g., keratins, IgGs, and extracellular proteins) and proteins that could not be unambiguously identified were manually removed. A set of 48 proteins that were enriched 2.5-fold or more in at least one of the H_2_O_2_-treated conditions could be listed (Supplementary Table S1).

**FIGURE 3:**
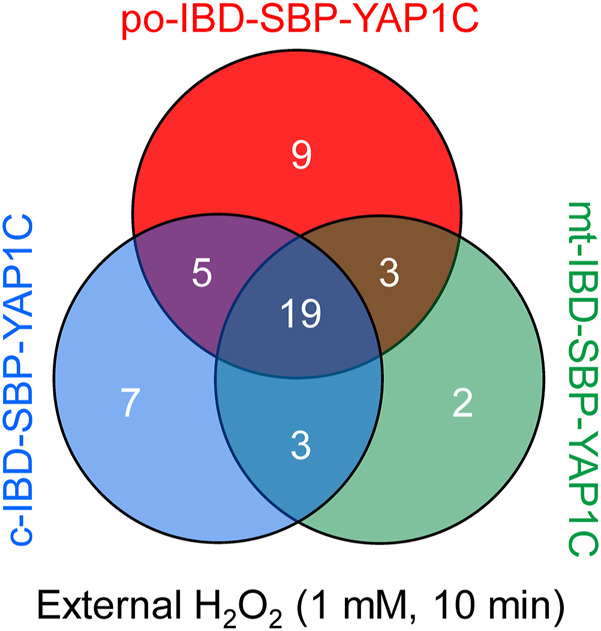
Venn diagram showing the number and overlap of po-, c-, and mt-IBD-SBP-YAP1C interactors upon treatment of Flp-In T-REx 293 cells with 1 mM H_2_O_2_.

Gene ontology analysis of the hits revealed significant enrichment for proteins primarily implicated in cell redox homeostasis and cellular oxidant detoxification (Supplementary Figure S3). These include GSR, PRDX1, PRDX2, PRDX3, PRDX4, PRDX5, PRDX6, TXN, and TXNRD2. Interestingly, while 19 out of 48 proteins were enriched in all H_2_O_2_-treated samples, each IBD-SBP-YAP1C fusion protein also retained unique interactors (Figure 3). This finding agrees with our previous results showing that differentially-localized IBD-SBP-YAP1C proteins form different complexes upon treatment of po-DD-DAO Flp-In T-REx 293 cells with D-Ala (Figure 2D).

To gain more insight into the binding selectivity of each IBD-SBP-YAP1C, we calculated the percentage distribution of each interactor with each of the YAP1C-fusion proteins (Supplementary Table S2). In line with expectations, this analysis revealed that 1) po-IBD-SBP-YAP1C predominantly interacts with ACOX1, a *bona fide* peroxisomal matrix protein (Yifrach et al., 2018), and proteins that show a partial peroxisomal localization (e.g., LDHB (Schueren et al., 2014) and MDH1 (Hofhuis et al., 2016)), 2) mt-IBD-SBP-YAP1C mainly interacts with genuine mitochondrial proteins (e.g., TXNRD2, ME2, ECI1, HSD17B10, and PRDX3 (Rath et al., 2021)), 3) c-IBD-SBP-YAP1C preferentially interacts with proteins that are predominantly located in the cytosol (e.g., DNPEP, HBA2, UCHL3, TUBB1, and CDK4 (Thul et al., 2017)), and 4) interactors located simultaneously in peroxisomes, mitochondria, and the cytosol (e.g., HSPA9 (Jo et al., 2020) and PRDX5 (Knoops et al., 2011)) are trapped by all YAP1C fusion proteins. However, diverse interactors known to be located in the cytosol and/or nucleus (e.g., BOLA2B, CSTB, HPRT1, PRDX1, PRDX2, PRDX6, PRMT5, SKP1, and WDR77 (Thul et al., 2017)) also bound to po- and mt-IBD-SBP-YAP1C, at least to a certain extent. This phenomenon can most likely be explained by the fact that a small but significant portion (rough estimation: 5–25%) of these IBD-SBP-YAP1C-fusion proteins still resides in the cytosol (Figure 1). Similar reasoning can be applied with regard to the observation that a small portion of ACOX1 interacts with c-IBD-SBP-YAP1C. Indeed, it can be expected that the c-IBD-SBP-YAP1C-interacting portion of ACOX1 represents the protein pool that has not yet been imported into peroxisomes. Here, it is also important to note that the percentage distribution of some interactors displayed an unexpected behavior. For example, some cytosolically located target proteins preferentially interacted with po-IBD-SBP-YAP1C (e.g., MARCKSL1, RPL13, and SET). A comprehensive explanation is currently lacking, but it cannot be excluded that portions of these proteins are partially associated with peroxisomes. However, this remains to be further investigated.

Finally, this experiment clearly shows that some interactors (e.g., PRDX1, PRDX2, and TXN) are already partially trapped by their respective IBD-SBP-YAP1Cs even in the absence of H_2_O_2_ treatment (Supplementary Table S1). On one hand, this may point to background contamination. However, a more likely explanation is that these interactors are extremely sensitive to sulfenic acid formation, a hypothesis supported by their recognized roles in localized, rapid, specific, and reversible redox-regulated signaling events (Hanschmann et al., 2013).

### The Cytosolic, Mitochondrial, and Peroxisomal Sulfenome Undergo Time-dependent Changes Upon Peroxisomal H_2_O_2_ Generation

**FIGURE 4:**
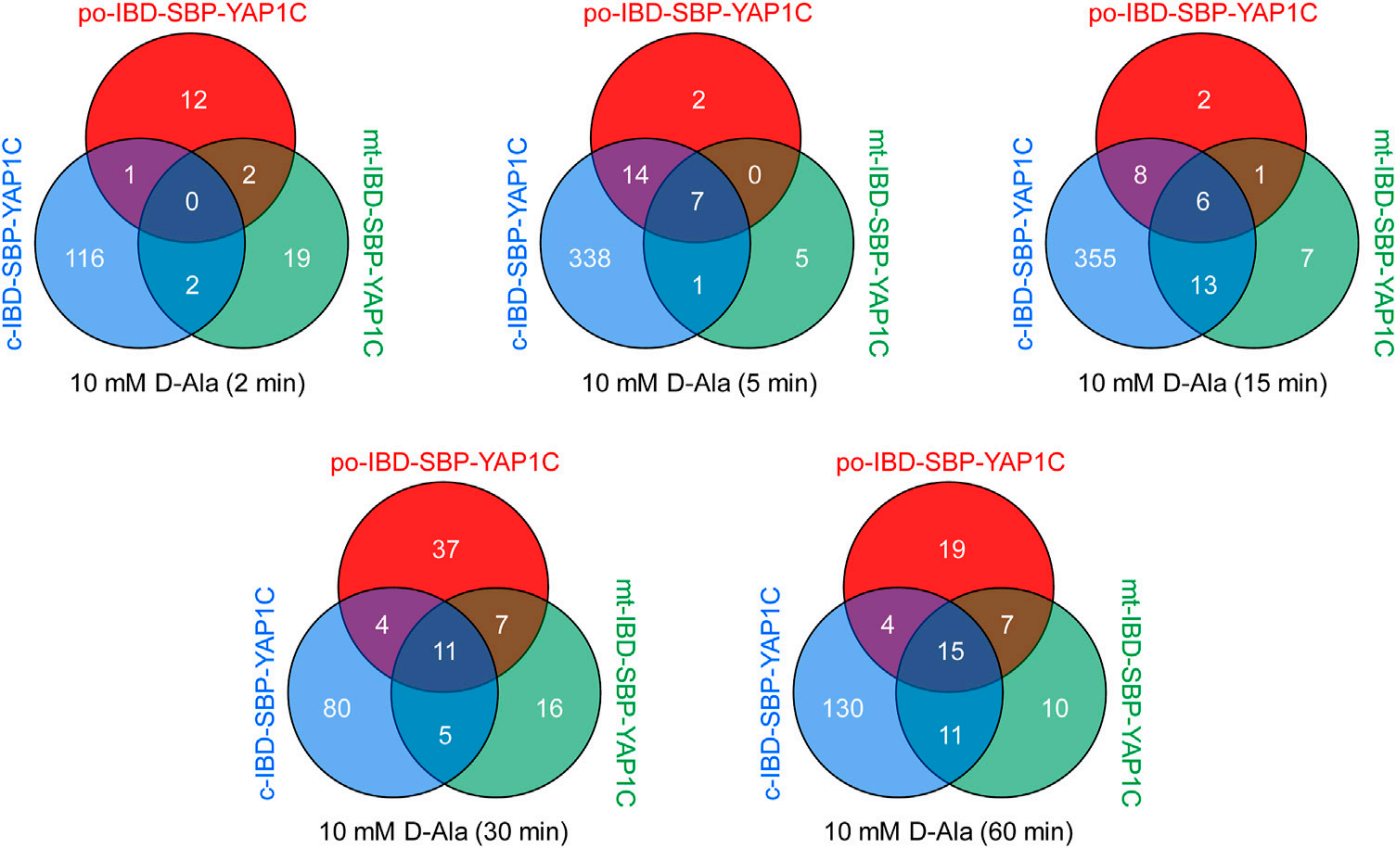
Venn diagrams showing the number and overlap of po-, c-, and mt-IBD-SBP-YAP1C interactors at different time points after treatment of Flp-In T-REx 293 cells expressing po-DD-DAO with 10 mM D-Ala.

**FIGURE 5:**
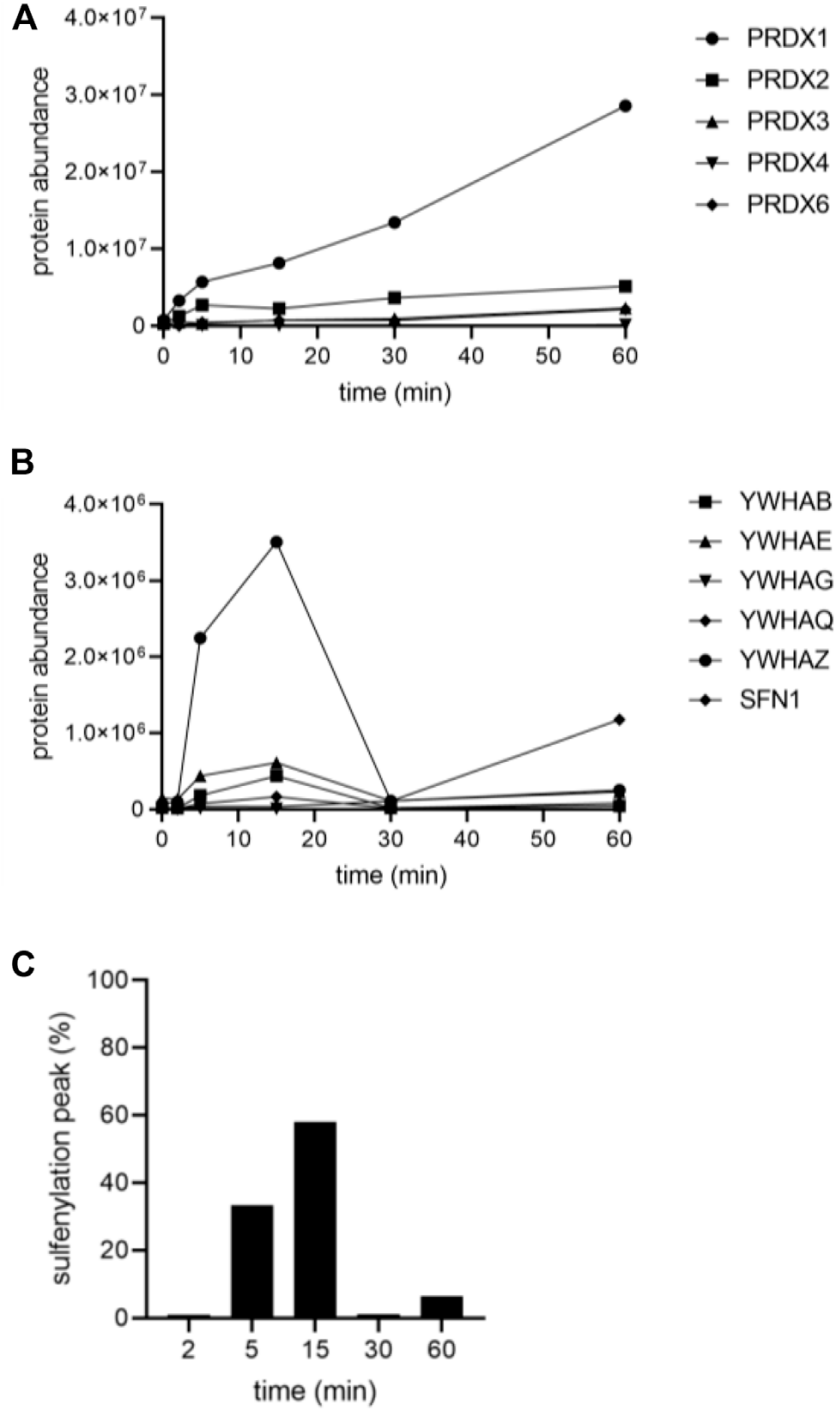
Sulfenylation kinetics of various c-IBD-SBP-YAP1C interactors in response to peroxisomal H_2_O_2_ production. Flp-In T-REx 293 cells expressing po-DD-DAO and c-IBD-SBP-YAP1C were incubated in DPBS containing 10 mM 3-AT and 10 mM D-Ala. At selected time points (0, 2, 5, 15, 30, and 60 min), free thiol groups were blocked with NEM. Next, the IBD-SBP-YAP1C-containing protein complexes were affinity purified, and the c-IBD-SBP-YAP1C interaction partners were eluted with reducing agent and processed for LC-MS/MS analysis (see Materials and Methods). **(A)** PRDX trapping by c-IBD-SBP-YAP1C (PRDX5 was not identified as hit). **(B)** 14-3-3 trapping by c-IBD-SBP-YAP1C (YWHAH was not identified as hit). **(C)** Percentage distribution of the sulfenylation peak time of all c-IBD-SBP-YAP1C interactors (n, 429).

A follow-up experiment was performed with Flp-In T-REx 293 cells expressing po-DD-DAO and compartment-specific variants of IBD-SBP-YAP1C and in which peroxisomal H_2_O_2_ production was induced by supplementing the assay medium with 10 mM D-Ala. However, this time we included different time points (0, 2, 5, 15, 30, and 60 min) (Supplementary Figure S4) and adapted the LC-MS/MS method providing a higher sensitivity in MS/MS. After validation of the MS results, a total of 444 unique proteins that were 2.5-fold or more enriched in at least one time point upon peroxisomal H_2_O_2_ production were retained. The number and overlap of interactors identified with po-, c-, or mt-IBD-SBP-YAP1C at each time point are visualized in Figure 4. From this overview, it is clear that the number of individual and common interactors of different IBD-SBP-YAP1Cs changed in function of time. One may argue that these differences are due to variations in experimental handling. However, given that peroxisomal H_2_O_2_ generation resulted in different but consistent response profiles for distinct interactor classes, this assumption is unlikely to hold. A specific example is shown for c-IBP-SBP-YAP1C, which traps multiple peroxiredoxins (PRDXs) and 14-3-3 proteins upon such treatment (Figures 5A,B). More examples can be found in Supplementary Table S3, Supplementary Table S4 and Supplementary Table S5, which respectively provide raw abundance heat maps of proteins trapped at different times by c-, po-, and mt-IBD-SBP-YAP1C in response to peroxisome-derived H_2_O_2_.

Manual inspection of the 75 different po-IBD-SBP-YAP1C interactors (Supplementary Table S4) surprisingly revealed the presence of only a few proteins that are frequently (e.g., CAT and HSD17B4) (Yifrach et al., 2018) or sporadically (e.g., HSPA9) (Jo et al., 2020) detected in peroxisomes. This observation can potentially be explained in different ways. For example, given that the peroxisomal H_2_O_2_ sensor po-roGFP-ORP1 is already almost fully oxidized in Flp-In T-REx 293 cells under basal conditions (Lismont et al., 2019a), it may well be that no redox-sensitive protein thiol groups were left as targets for oxidation by newly formed H_2_O_2_. On the other hand, it cannot be ruled out that the peroxisomal proteins trapped by po-IBD-SBP-YAP1C represent the cytosolic protein pools that have not yet been imported into peroxisomes. To gain more insight into this problem, we compared the response kinetics of CAT and HSD17B4 to peroxisome-derived H_2_O_2_ in cells expressing po- or c-IBD-SBP-YAP1C (Supplementary Figure S5). From this figure, it is clear that 1) depending on the interactor and time point, the amount of protein trapped by po-IBD-SBP-YAP1C is up to 1.5-fold higher (e.g., CAT, 30 min) or up to 200-fold lower (e.g., HSD17B4, 15 min) than the amount trapped by c-IBD-SBP-YAP1C, and 2) the sulfenylation profiles of po-IBD-SBP-YAP1C (left panels) and c-IBD-SBP-YAP1C (middle panels) interactors can exhibit a bimodal behavior, thereby reflecting a heterogeneous character of protein thiol oxidation (see Discussion). The observation that the capturing ratios of the peroxisomal targets by po- and c-IBD-SBP-YAP1C vary between different time points (e.g., compare CAT at 5 and 30 min), demonstrates that at least a portion of these complexes was formed inside peroxisomes. However, it is also clear that, at least for HSD17B4, complex formation inside peroxisomes is a rather negligible phenomenon compared to complex formation in the cytosol.

**FIGURE 6:**
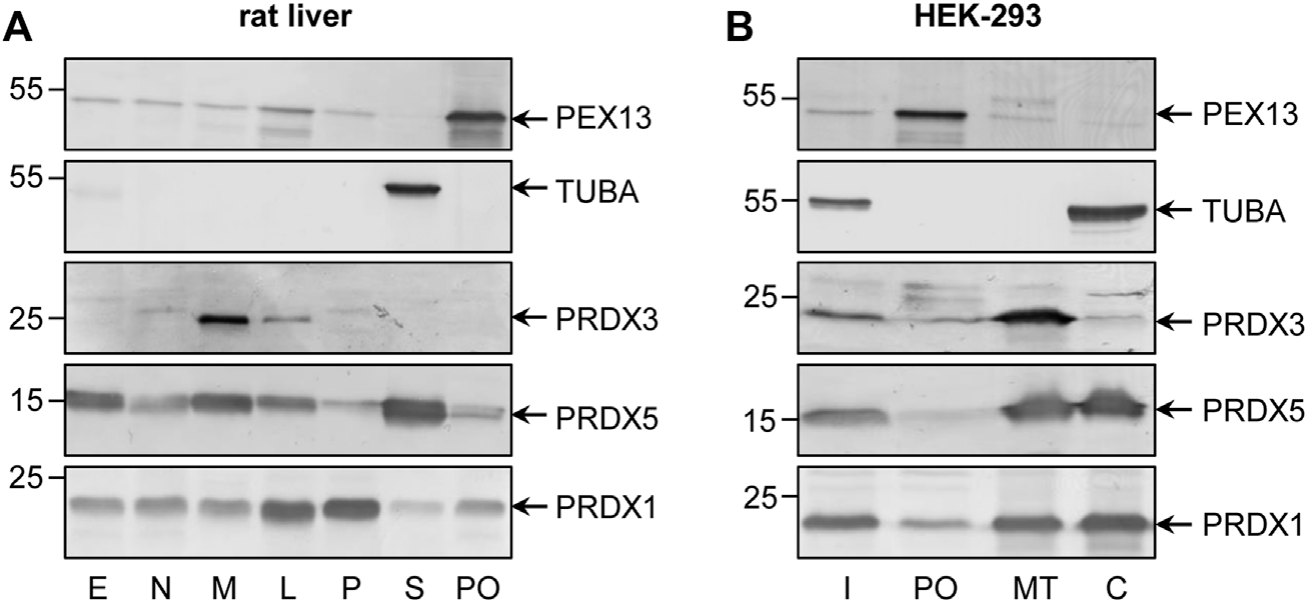
Subcellular distribution of PRDX1 in rat liver and HEK-293 cells. **(A)** Rat liver protein (20 µg) present in a post-nuclear fraction (E), a nuclear fraction (N), a heavy mitochondrial fraction (M), a light mitochondrial fraction (L), a microsomal fraction (P), the cytosol (S), or purified peroxisomes (PO) were subjected to SDS-PAGE and processed for immunoblotting with antibodies directed against PEX13 (peroxisomes), TUBA (cytosol), PRDX3 (mitochondria), PRDX5 (cytosol, mitochondria, nucleus, and peroxisomes), or PRDX1. **(B)** Total cell homogenates from Flp-In T-REx 293 cells were fractionated by differential centrifugation to yield a 1,500 x g supernatant (Input, (I)) that was subsequently subjected to Nycodenz gradient centrifugation (Lismont et al., 2019c). The I fraction (8% of the amount loaded onto the gradient) and equal volumes of gradient fractions enriched in peroxisomes (PO), mitochondria (MT), or the cytosol (C) were processed for immunoblot analysis as described for the rat liver fractions. The migration points of relevant molecular mass markers (expressed in kDa) are shown on the left. Specific proteins are marked by arrows.

Unlike external H_2_O_2_ addition, peroxisomal H_2_O_2_ production did not result in po-IBD-SBP-YAP1C-mediated trapping of ACOX1 or MDH1. Nonetheless, such treatment did result in the trapping of various cytosolic, cytoskeletal, and plasma membrane-associated proteins (Supplementary Table S4), many of which are known to be sulfenylated on at least one cysteine residue (Wang et al., 2021). Importantly, given that 1) we and others have previously demonstrated that peroxisome-derived H_2_O_2_ can efficiently permeate across the peroxisomal membrane (Mueller et al., 2002; Lismont et al., 2019a; Laporte et al., 2020), 2) a small portion of po-IBD-SBP-YAP1C is mislocalized to the cytosol (Figure 1), and 3) the fraction of interactor bound to po-IBD-SBP-YAP1C at the peak of protein sulfenylation is on average less than 10% of the amount of interactor bound to c-IBD-SBP-YAP1C, it is safe to conclude that the majority of cytosolic (e.g., PSMA7), cytoskeletal (e.g., POF1B), and plasma membrane-associated (e.g., ANXA2) interactors were trapped by the residual cytosolic fraction of po-IBD-SBP-YAP1C (Supplementary Figure S6). However, for some cytosolic interactors (e.g., PRDX1, PRDX2, SKP1, and TXN), the amount of po-IBD-SBP-YAP1C-bound protein was much higher than what one would expect from the estimated percentage of mislocalized po-IBD-SBP-YAP1C (Supplementary Figure S7). Although it was not the scope of this work to dig into the subcellular localization of all these and other targets of peroxisome-derived H_2_O_2_, we decided to explore this intriguing observation in more detail for PRDX1. Upon immunoblot analysis of subcellular fractions derived from HEK-293 cells or rat liver, a small but significant portion of PRDX1 appears to be associated with peroxisomes (Figure 6), thereby strengthening our working hypothesis.

A careful examination of the 53 different mt-IBD-SBP-YAP1C interactors (Supplementary Table S5) revealed the presence of nine proteins (ATP5F1A, ATP5F1B, GSR, HSP90AA1, HSPD1, PC, PCCB, PRDX3, and VDAC1) with a *bona fide* (partial) mitochondrial localization (Rath et al., 2021), thereby providing direct molecular evidence for the previously established peroxisome-mitochondria redox connection (Lismont et al., 2015). As observed for po-IBD-SBP-YAP1C (Supplementary Table S4; Supplementary Figure S6), mt-IBD-SBP-YAP1C also captured some cytosolic (e.g., S100A14), cytoskeletal (e.g., POF1B), and plasma membrane-associated (e.g., ANXA2) interactors (Supplementary Figure S8). This observation strengthens our prior interpretation (Supplementary Table S2) that also a small portion of mt-IBD-SBP-YAP1C is not yet imported into mitochondria. For comparison, we also included PRDX3, a mitochondrial member of the PRDX family (Supplementary Figure S8).

We also identified a subset of 429 different c-IBD-SBP-YAP1C interactors (Supplementary Table S3). KEGG pathway analysis revealed enrichment for proteins implicated, among others, in ribosome biology, carbon metabolism, biosynthesis of amino acids, and proteasome functioning (the -log_10_ (p_adj_) values are 5.162 × 10^–27^, 3.512 × 10^–8^, 2.293 × 10^–6^, and 1.325 × 10^–6^, respectively). Surprisingly, despite the fact that it is well known that H_2_O_2_ can, directly or indirectly, oxidatively modify different classes of proteins involved in signal transduction (e.g., kinases, phosphatases, proteases, antioxidant enzymes, transcription factors, etc.), no transcription factors were found to interact with c-IBD-SBP-YAP1C. One potential explanation for this finding is that members belonging to this group of proteins are oxidatively modified via redox relay, and not through direct oxidation of redox-sensitive cysteines to sulfenic acid (see Discussion). Another interesting observation is that with the exception of proteins involved in the maintenance of cellular redox homeostasis (e.g., GSR, PRDX1, PRDX2, PRDX3, PRDX4, PRDX6, TXN, and ERP44), virtually all other interactors display a sulfenylation peak at 5 or 15 min (Figure 5C). In addition, proteins belonging to the same protein family share in general a common pattern (e.g., compare the peak time values in Figure 5 and Supplementary Figure S9). Protein families that, besides the PRDXs (Figure 5A) and 14-3-3 (Figure 5B), are abundantly modified by peroxisome-derived H_2_O_2_ include constituents of the cytoskeleton (Supplementary Figures S9A,B), annexins (Supplementary Figure S9C), protein chaperones (Supplementary Figure S9D), S100 proteins (Supplementary Figure S9E), and negative regulators of endopeptidase activity (Supplementary Figure S9F). Finally, it is worth noting that some c-IBD-SBP-YAP1C interactors (e.g., ANXA2, DSG1, DSP, FLG2, GAPDH, and JUP) already appear to be considerably sulfenylated under basal conditions (Supplementary Table S3). Once again, this observation strongly supports a role for these proteins in redox-regulated housekeeping signaling pathways. However, another potential explanation is that the high basal sulfenylation state of some of these proteins is a confounding effect of the hyperoxic *in vitro* cell culture environment (typically ∼18% O_2_), which differs from the *in vivo* situation (∼1–6% O_2_) (Stuart et al., 2018).

### The Cytosolic Sulfenome Responds to Exogenous H_2_O_2_ in a Dose-dependent Manner

In a laboratory setting (patho)physiological scenarios of how H_2_O_2_ can drive cellular signaling events are often mimicked by the external addition of this oxidant to cultured cells. In order to assess how H_2_O_2_ levels and sulfenylation responses in our DD-DAO-based approach compare to this often-used strategy, we treated cells with different concentrations of H_2_O_2_, ranging between 10 µM and 1 mM, for 10 min (Supplementary Figure S10). Using cells expressing c-IBD-SBP-YAP1C, we identified 326 proteins that were 2.5-fold or more enriched in at least one of the treated conditions (Supplementary Table S6). Note that, due to the increased sensitivity settings in this LC-MS/MS run, the number of hits greatly exceeded the number of targets identified in the initial validation experiment (Supplementary Table S1).

**FIGURE 7:**
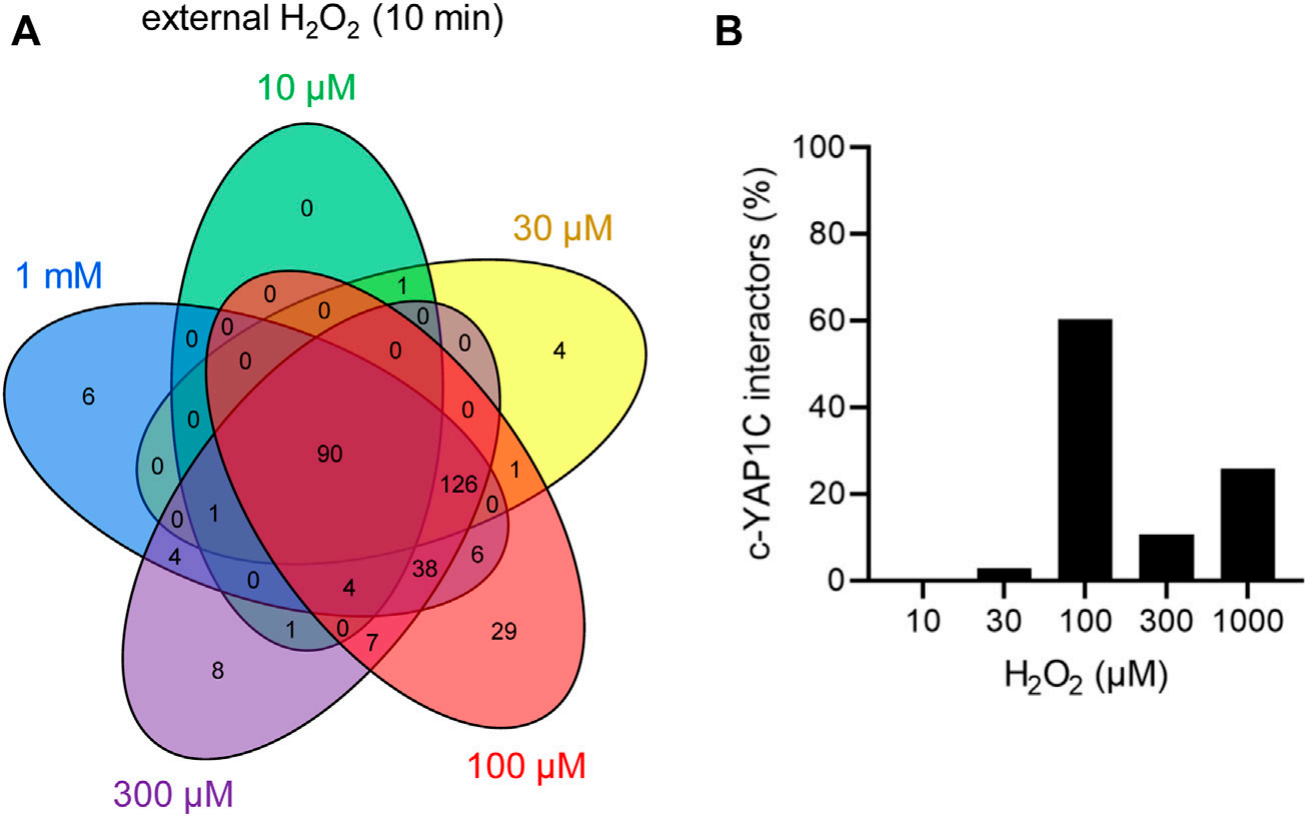
The cytosolic sulfenome in response to different concentrations of extracellular H_2_O_2_. **(A)** Venn diagram showing the number and overlap of significantly enriched interactors of c-IBD-SBP-YAP1C upon treatment of Flp-In T-REx 293 cells with different concentrations of external H_2_O_2_ for 10 min. **(B)** Percentage distribution of total c-IBD-SBP-YAP1C (c-YAP1C) interactors (*n*, 326) with a sulfenylation peak at the indicated H_2_O_2_ concentration.

**FIGURE 8:**
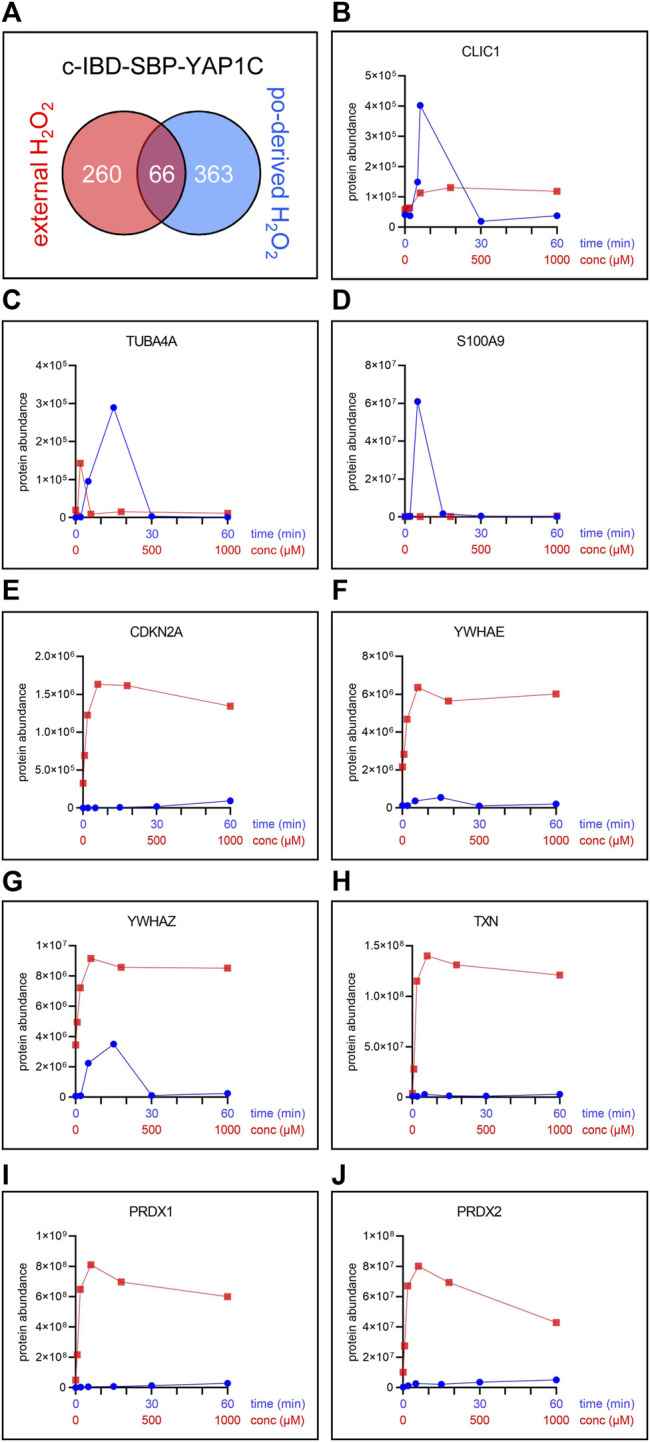
The pool and sulfenylation profiles of c-IBD-SBP-YAP1C interactors differ depending on the H_2_O_2_ source. **(A)** Venn diagram showing the number and overlap of significantly enriched interactors of c-IBD-SBP-YAP1C upon treatment of po-DD-DAO Flp-In T-REx 293 cells (i) for any time period (2, 5, 15, 30, or 60 min) with 10 mM D-Ala (Supplementary Table S3) or (ii) with any concentration (10, 30, 100, 300, or 1,000 µM) of external H_2_O_2_ for 10 min (Supplementary Table S6). **(B–J)** Sulfenylation profiles of a selected set of c-IBD-SBP-YAP1C interactors upon peroxisomal H_2_O_2_ production (time curve; in blue) or addition of external H_2_O_2_ for 10 min (concentration curve; in red). Protein abundances are based on peptides that were commonly retrieved in the experiments shown.

The number and overlap of interactors identified upon treatment of the cells with different H_2_O_2_ concentrations are visualized in Figure 7A. From these data, it can be deduced that treatment with 100 µM H_2_O_2_ yields the highest number of sulfenylated proteins. This can be explained by the fact that, at this concentration of H_2_O_2_, 60% of all targets reach their sulfenylation peak (Figure 7B), including the antioxidant defense enzymes (Supplementary Table S6) that did not even reach their peak within 1 h of peroxisomal H_2_O_2_ production (Supplementary Table S3). Interestingly, PRDX5 is the only PRDX that was exclusively sulfenylated by externally added H_2_O_2_ (Supplementary Table S6). This may be explained because 1) PRDX5 has the lowest expression level of all PRDXs in HEK-293 cells (Geiger et al., 2012) and 2) its preferred substrates are lipid peroxides instead of hydrogen peroxide (Knoops et al., 2011). Given that PRDX5 is already more than 2.5-fold enriched from 10 µM H_2_O_2_ onwards, peroxisomal H_2_O_2_ production by DD-DAO is far less stringent than the external addition of 10 µM H_2_O_2_. Taking into consideration that data in the literature suggest a 390- to 650-fold concentration difference between the extra- and intracellular H_2_O_2_ levels (Huang and Sikes, 2014; Lyublinskaya and Antunes, 2019), peroxisomal H_2_O_2_ production most likely results in intracellular concentrations of less than 15–26 nM. This claim is supported by the observation that within the time frame of the experiment (60 min), the equilibrium between peroxisomal H_2_O_2_ production by DD-DAO and the cell’s antioxidant defense mechanisms has not yet been reached (as demonstrated by the fact that antioxidant enzymes did not reach their sulfenylation peak). Here, it is also worth mentioning that there is relatively little overlap between the protein targets of peroxisome-derived and externally added H_2_O_2_ (Figure 8A). When comparing the sulfenylation profiles upon addition of external H_2_O_2_ (concentration curve) or peroxisomal H_2_O_2_ production (time curve), it is clear that depending on the H_2_O_2_ source, different proteins show distinct responses. A set of examples is shown in Figures 8B–J. Altogether, these findings highlight that data obtained with external H_2_O_2_ cannot simply be extrapolated to internally produced H_2_O_2_, which reflects a more physiological condition.

**FIGURE 9:**
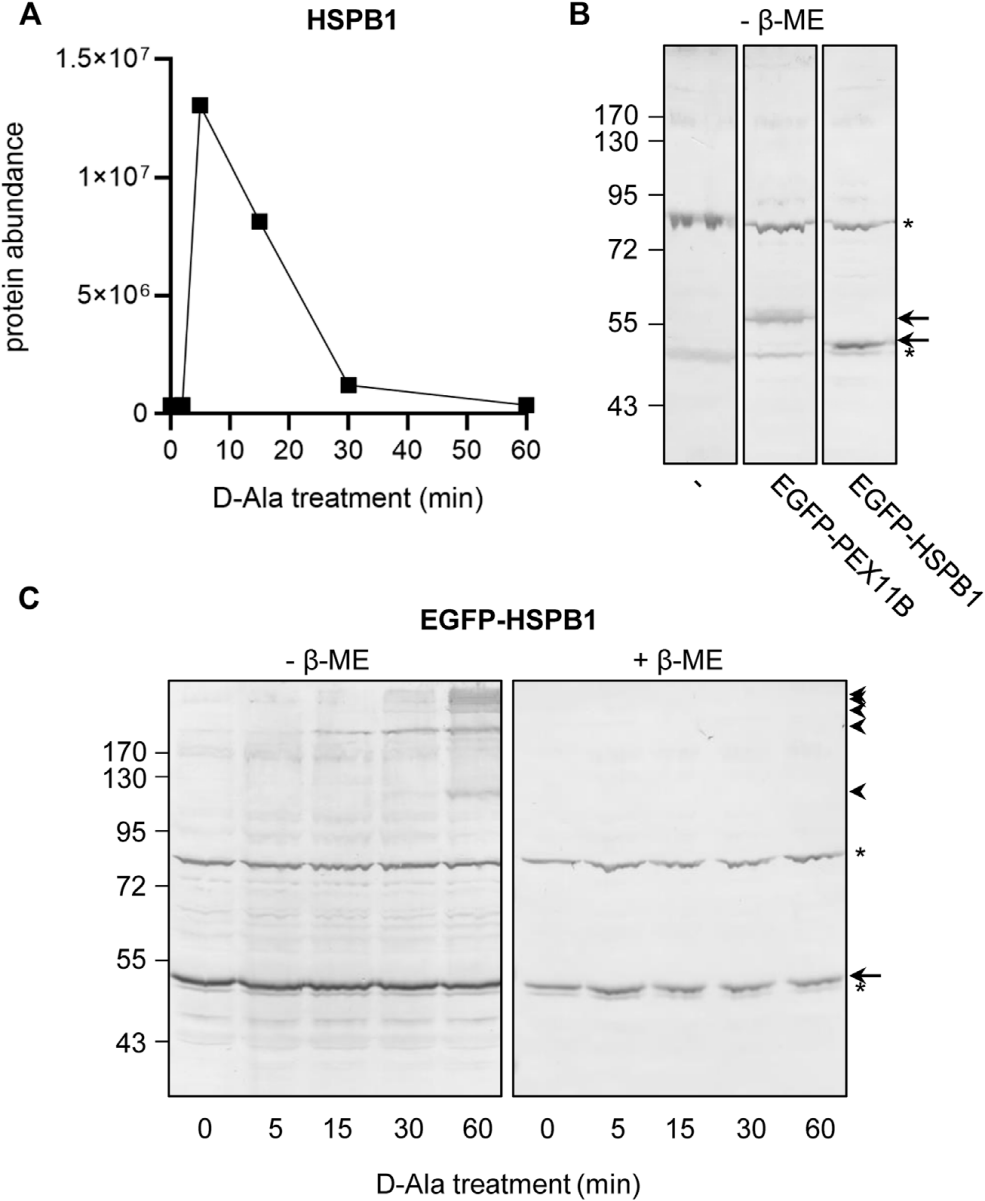
Sulfenylation and disulfide bond formation kinetics of HSPB1 in response to peroxisomal H_2_O_2_ production. Flp-In T-REx 293 cells expressing po-DD-DAO and containing c-IBD-SBP-YAP1C **(A)** or not **(B,C)** were transfected **(B,C)** or not **(A)** with a plasmid encoding no EGFP-fusion protein (−), EGFP-PEX11B, or EGFP-HSPB1. The cells were incubated in DPBS containing 10 mM 3-AT and 10 mM D-Ala. At the indicated time points, the cells were processed as detailed in the legend of **(A)** Figure 5, or **(B,C)** processed for SDS-PAGE under non-reducing (-β-ME) or reducing (+β-ME) conditions and subsequently subjected to immunoblot analysis with antibodies specific for EGFP. The migration points of relevant molecular mass markers (expressed in kDa) are shown on the left. The arrows and arrowheads mark the non-modified and oxidatively modified proteins, respectively. Note that panel B was included to document the non-specific immunoreactive bands (marked by asterisks) of the anti-EGFP antiserum.

Strikingly, whereas upon peroxisomal H_2_O_2_ production the sulfenylation peak rapidly decreases to baseline levels for most proteins, treatment with external H_2_O_2_ most often results in a slower and rather modest decrease (Figures 8B–J, compare the blue and red profiles). Given that we have previously shown that po-DD-DAO-mediated H_2_O_2_ production results in steadily increasing levels of cytosolic H_2_O_2_ and disulfide bond formation over time (Lismont et al., 2019a), a potential explanation is that the fast decrease in sulfenylation observed under this condition represents a combined effect of 1) a thioredoxin (TXN)-mediated reduction of the disulfide bond between IBD-SBP-YAP1C and its interactors (Izawa et al., 1999), and 2) a continuous depletion of freely available redox-sensitive cysteine thiols (e.g., due to overoxidation or disulfide bond formation with other proteins). Importantly, our observation that under conditions of oxidative stress transient disulfide bond formation between HSPB1 and c-IBD-SBP-YAP1C (Figure 9A) precedes previously reported (Zavialov et al., 1998) disulfide-mediated changes in the oligomeric state of HSPB1 (Figure 9C), is in line with this view. On the other hand, upon external H_2_O_2_ addition, the modest decrease in sulfenylation after peaking may be explained by reduced availability of TXN and other thiol-disulfide reductases as a consequence of their overoxidation, a phenomenon supported by the observation that also these enzymes themselves are less sulfenylated at these H_2_O_2_ concentrations (Figures 8H–J). Finally, the observation that proteins can be released from c-IBD-SBP-YAP1C underscores the transient nature of these disulfide bonds, even in an oxidizing environment.

### Assessment of Potential Pitfalls

**FIGURE 10:**
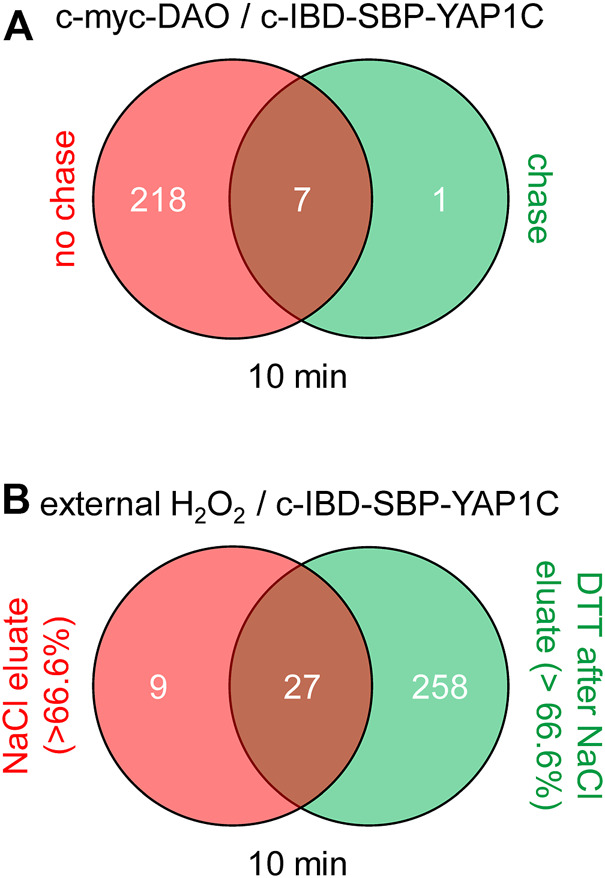
Venn diagrams showing the number and overlap of c-IBD-SBP-YAP1C interactors under different experimental conditions. **(A)** c-DD-DAO-driven H_2_O_2_ production in the absence or presence of a 24-h chase period. **(B)** External H_2_O_2_ addition and elution of the interactors first with 1 M NaCl and subsequently with 10 mM DTT. The threshold values to belong to a specific group reflect the percentage of protein recovered in each fraction relative to the total protein abundance (red: > 66.6% in the NaCl eluate; green: > 66.6% in the DTT after NaCl eluate; brown: less than 66.6% in both eluates).

The findings presented thus far clearly demonstrate that the YAP1C-based sulfenome mining approach is a very powerful and efficient tool to identify targets of peroxisome-derived H_2_O_2_. As mentioned above and described elsewhere (Lismont et al., 2019a), we used Flp-In T-REx 293-derived cell lines in which the expression levels and stability of po-DD-DAO can be strictly controlled to overcome possible interfering effects of H_2_O_2_ produced by newly synthesized DD-DAO that has not yet been imported into peroxisomes. To document the validity of this assumption at the proteome level, we also performed a sulfenome mining experiment with Flp-In T-REx 293 cells expressing c-DD-DAO and c-IBD-SBP-YAP1C (Supplementary Figure S11). Importantly, even in a condition where initially 100% of DD-DAO was located in the cytosol, only 8 out of the 226 identified targets were enriched in the chase condition (Figure 10A; Supplementary Table S7). In addition, to gain more insight into target proteins that may be indirectly retained on the affinity matrix through electrostatic interaction with truly sulfenylated proteins (Liebthal et al., 2020), Flp-In T-REx 293 cells expressing c-IBD-SBP-YAP1C were treated with 1 mM H_2_O_2_ for 10 min and, after enrichment of the corresponding c-IBD-SBP-YAP1C complexes and completing the normal washing procedure, the streptavidin column was first three times eluted with high salt (1 M NaCl) and subsequently three times with DTT (this experiment was done in parallel with the experiment shown in Supplementary Figure S10). LC-MS/MS analysis of the eluates revealed the presence of 294 distinct interactors, of which only 9 predominantly eluted in a NaCl-dependent manner (Figure 10B; Supplementary Table S8). These findings confirm that the vast majority of targets identified are *bona fide* sulfenylated proteins. Finally, one can argue that one biological replicate per condition may not suffice to draw reliable conclusions. However, the aim of this study was not to generate a full inventory of the responsiveness of redox-sensitive proteins to peroxisome-derived H_2_O_2_, but rather to provide an insight into the dynamics and localization of the major H_2_O_2_ targets. Therefore, we have adopted extremely stringent validation criteria for peptide identification (PEP of peptide spectral match (PSM) < 10^–3^ in at least one of the conditions of the experiment), resulting in proteins that are very confidently identified but that are only the tip of the iceberg in terms of protein abundance. In addition, in the following section, we discuss additional observations that support the reliability of our data.

### Evaluation of the Reliability of the YAP1C-Based Sulfenome Mining Approach

**FIGURE 11:**
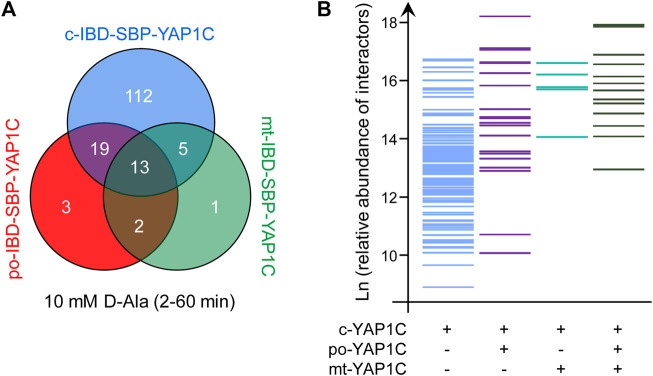
Reproducibility and sensitivity of the IBD-SBP-YAP1C-interactor enrichment workflow. **(A)** Venn diagram showing the number and overlap of all po-, c-, and mt-IBD-SBP-YAP1C interactors with a predominantly cytosolic distribution pattern after treatment of Flp-In T-REx 293 cells expressing po-DD-DAO with 10 mM D-Ala for 2, 5, 15, 30, and 60 min. Note that only the residual pools of po-IBD-SBP-YAP1C and mt-IBD-SBP-YAP1C that are not yet imported in peroxisomes and mitochondria, respectively, have the potential to trap cytosolically located sulfenylated proteins. **(B)** Bar plot showing the relative abundance (expressed as Ln (x)) of predominantly cytosolic c-(IBD-SBP-)YAP1C interactors that form disulfide bridges or not with residual cytosolic po- and/or mt-(IBD-SBP-)YAP1C.

To assess the reproducibility of our sulfenome mining approach, we exploited the fact that small but significant amounts of po- and mt-IBD-SBP-YAP1C are still located in the cytosol (Figure 1, Supplementary Table S2). Specifically, we selected all predominantly cytosolic targets of peroxisome-derived H_2_O_2_ that were trapped in independent experiments by c-IBD-SBP-YAP1C (Supplementary Table S3), po-IBD-SBP-YAP1C (Supplementary Table S4), or mt-IBD-SBP-YAP1C (Supplementary Table S5). Strikingly, upon comparison, only 5 out of the 37 cytosolic interactors of po-IBD-SBP-YAP1C and 3 out of the 21 cytosolic interactors of mt-IBD-SBP-YAP1C were not present in the pool of c-IBD-SBP-YAP1C interactors (Figure 11A). This clearly demonstrates that the interactors identified are specific and not merely a random result of a single experiment. The consistency of our data is further strengthened by the fact that most of the po- and mt-IBD-SBP-YAP1C cytosolic interactors identified correspond to the more abundantly captured interaction partners of c-IBD-SBP-YAP1C (Figure 11B). Note that the different thresholds of sensitivity in the trapping of sulfenylated proteins in the cytosol by c-, po-, and mt-IBD-SBP-YAP1C can be explained by their relative concentrations in this subcellular compartment.

**FIGURE 12:**
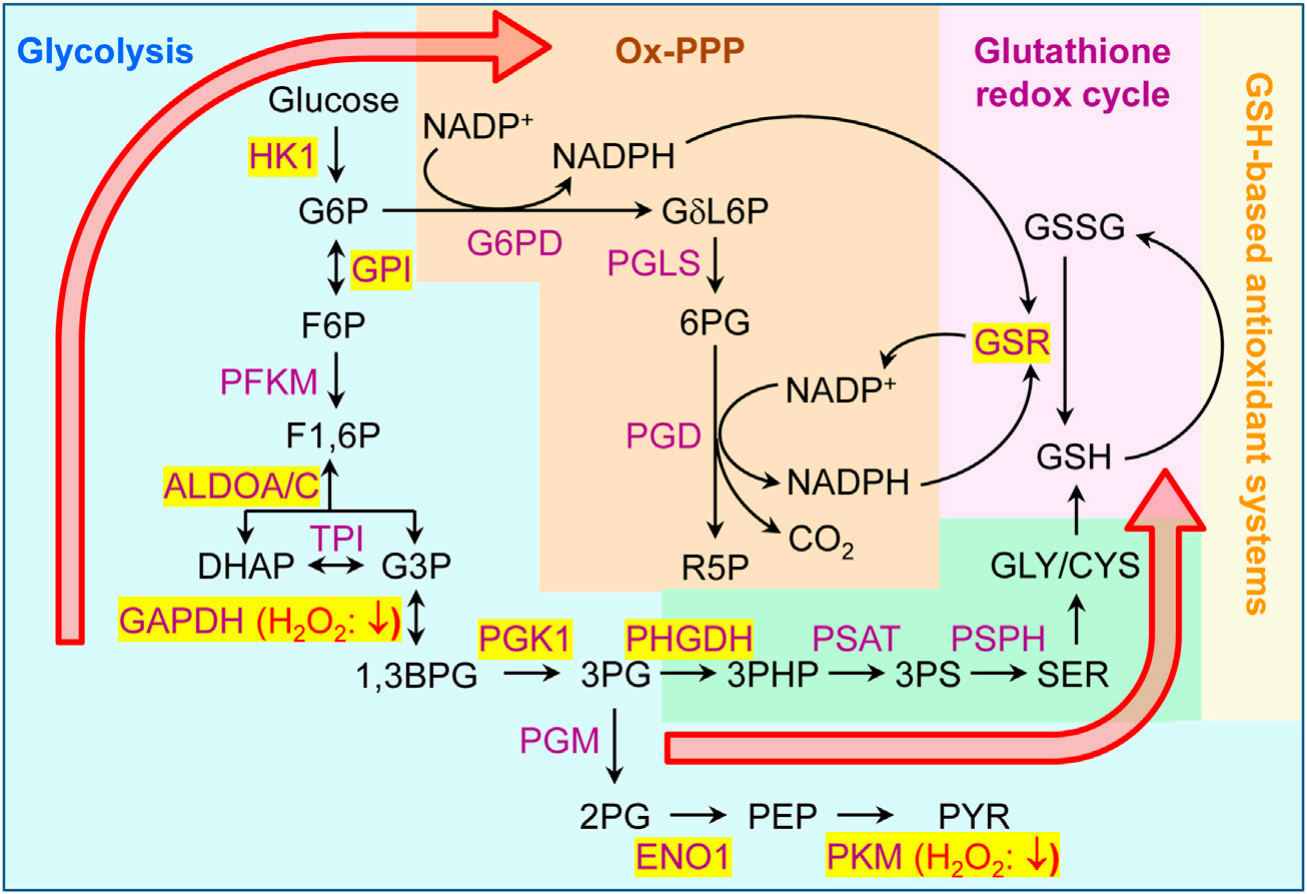
Peroxisome-derived H_2_O_2_ can oxidatively modify multiple glycolytic enzymes. Schematic outlining a simplified overview of glycolysis, the oxidative branch of the pentose phosphate pathway (Ox-PPP), and the glutathione redox cycle. Metabolic enzymes and metabolites are indicated in purple and black, respectively. Enzymes that become sulfenylated upon peroxisomal H_2_O_2_ production are shown on a yellow background. Based on evidence found in the literature (Mullarky and Cantley, 2015), oxidation of GAPDH and PKM respectively (i) redirects the glycolytic flux towards ox-PPP (through an increase in the metabolites upstream of GAPDH), and (ii) increases the synthesis of the glutathione precursors glycine and cysteine (through activation of serine synthesis by a buildup of 2PG). 1,3BPG, 1,3-bisphosphoglycerate; 2PG, 2-phosphoglycerate; 3PG, 3-phosphoglycerate; 3PHP, 3-phosphohydroxypyruvate; ALDO, aldolase, CYS, cysteine; DHAP, dihydroxyacetone phosphate; ENO, enolase; F1,6P, fructose-1,6-bisphosphate; F6P, fructose-6-phosphate; G3P, glyceraldehyde-3-phosphate; G6P, ribulose 5-phosphate; GAPDH, glyceraldehyde-3-phosphate dehydrogenase; GLY, glycine; GPI, glucose-6-phosphate isomerase; GSR, glutathione-disulfide reductase; GSH, glutathione (reduced); GSSG, glutathione (oxidized); HK, hexokinase; NADP+, nicotinamide adenine dinucleotide phosphate (oxidized); NADPH, nicotinamide adenine dinucleotide phosphate (reduced); PFKM, phosphofructokinase, muscle; G6PD, glucose-6-phosphate dehydrogenase; GδL6P, 6-phospho-D-glucono-1,5-lactone; PEP, phosphoenolpyruvate; PGD, 6-phosphogluconate; PGK, phosphoglycerate kinase; PGM, phosphoglucomutase; PHGDH, phosphoglycerate dehydrogenase; PKM, pyruvate kinase; PS, phosphoserine; PSAT, phosphoserine aminotransferase; PSPH, phosphoserine phosphatase; PYR, pyruvate; SER, serine.

To validate our findings on a more conceptual level, we connected our data to previous observations that oxidative stress rewires cellular carbohydrate metabolism (Grant, 2008; Mullarky and Cantley, 2015). In this context, it is important to highlight that 1) NADPH, generated during the metabolism of glucose via the oxidative arm of the pentose phosphate pathway (Ox-PPP), acts as an important redox cofactor that fuels most cellular antioxidant systems, and 2) oxidative stress can inhibit glycolytic enzymes as a controlled response that redirects the metabolic flux from glycolysis to PPP (Grant, 2008). Both carbohydrate fluxes are directly connected via glucose-6-phosphate, a common intermediate that, upon isomerization to fructose-6-phosphate, continues through glycolysis and, upon oxidation to 6-phospho-D-glucono-1,5-lactone, enters the Ox-PPP (Figure 12). While the Ox-PPP enzyme activities appear to be maintained during oxidant exposure, such treatment has been shown to inhibit multiple glycolytic enzymes, including GAPDH and PKM, by directly oxidizing cysteine residues (Mullarky and Cantley, 2015). Oxidative inhibition of GAPDH and PKM have respectively been shown 1) to redirect glycolytic flux towards Ox-PPP (through an accumulation of metabolites upstream of GAPDH), and 2) to increase the biosynthesis of glycine and cysteine, two glutathione precursors (through the build-up of 2-phosphoglycerate and subsequent activation of the serine synthesis pathway), thereby conferring cells resistant to oxidative stress (Mullarky and Cantley, 2015). Interestingly, a careful analysis of our sulfenome mining data indicated that multiple glycolytic enzymes, including GAPDH and PKM, are also targets of peroxisome-derived H_2_O_2_ (see Figure 12).

Lastly, also the conserved sulfenylation profiles between targets belonging to the same protein family (Supplementary Figure S9) as well as the observation that a decrease in the sulfenylation peak of HSPB1 coincides with an increase in disulfide bond formation (Figure 9) support the idea that our findings are solid.

### Identification of Oxidatively Modified Cysteines

**FIGURE 13:**
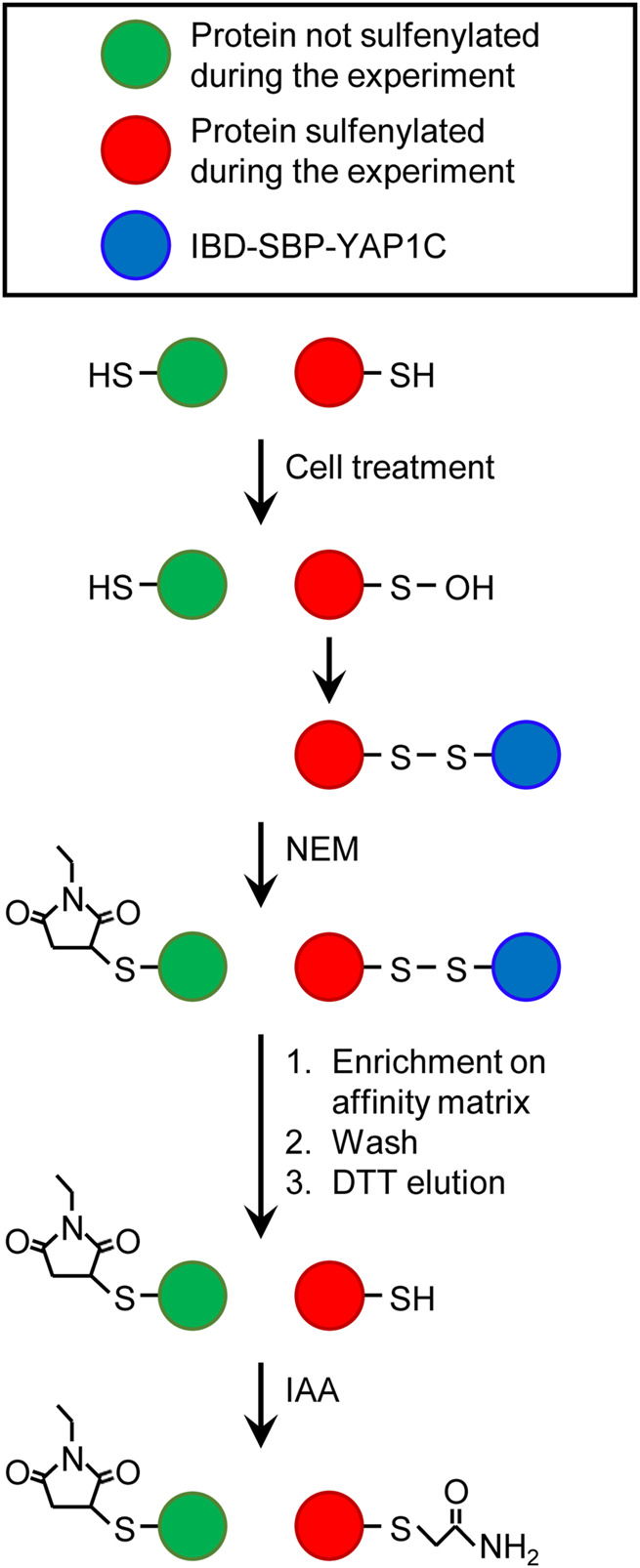
Differential alkylation of cysteine thiols and sulfenic acids. After cell treatment, free thiols are immediately blocked with N-ethyl maleimide (NEM). Proteins containing at least one sulfenylated cysteine that forms a disulfide bridge with IBD-SBP-YAP1C are enriched on the streptavidin affinity matrix, extensively washed, eluted with dithiothreitol (DTT), and alkylated with iodoacetamide (IAA). This results in a differential alkylation of originally reduced or sulfenylated cysteines within the affinity-purified proteins. For more details about the experimental procedure, see Materials and Methods.

In our protocol, free and oxidatively modified cysteines are differentially alkylated (Figure 13), providing more information on which cysteines are oxidized. In short, after cell treatment, free thiols are irreversibly blocked with NEM, cells are lysed, and IBD-SBP-YAP1C-containing protein complexes are enriched on the streptavidin affinity matrix. Upon DTT elution, the originally sulfenylated cysteines captured by IBD-SBP-YAP1C are reduced and become available for iodoacetamide alkylation, thereby resulting in the formation of an S-carbamidomethyl cysteine. As LC-MS/MS analysis can distinguish between different cysteine modifications, it is possible to determine at what stage in the protocol the cysteines of the identified peptides were alkylated. Here, it is essential to point out that S-carbamidomethylation does not necessarily indicate that the cysteine was indeed sulfenylated, since S-carbamidomethylated cysteines can also result from pre-existing disulfide bridges or cysteines that are inaccessible to NEM. In addition, as LC-MS/MS analysis does not detect every peptide of a protein, the detection of redox-sensitive cysteine-containing peptides is no absolute requirement to categorize a hit as an authentically sulfenylated protein. In the 784 IBD-SBP-YAP1C-interactors that were identified during the time course of our experiments, we could detect 150 proteins containing at least one S-carbamidomethyl cysteine (Supplementary Table S9). As could be expected, the number of detected S-carbamidomethylated cysteines is determined by the sensitivity settings of the LC-MS/MS run as well as by the stringency of the treatment which intrinsically correlates with peptide abundance (e.g., treatment of cells with external H_2_O_2_ resulted in more S-carbamidomethyl cysteines than H_2_O_2_ production inside peroxisomes). A comparative analysis of our data with the list of proteins in the iCysMod database revealed that, of the 337 redox-sensitive cysteines identified, 91 cysteines have already been reported to be sulfenylated by others, 172 cysteines were already shown to undergo other oxidative thiol modifications, and 74 cysteines represent novel redox-regulated cysteines in humans (Supplementary Table S9).

## Discussion

Currently, it is widely accepted that peroxisomes can act as H_2_O_2_ signaling platforms, thereby conveying metabolic information into redox signaling events (Fransen and Lismont, 2019; He et al., 2021). However, little is known about the molecular targets of peroxisomal H_2_O_2_ and, to address this gap, we designed an efficient and unique sulfenome mining approach to capture and identify such targets in a dynamic manner. From our results, it is clear that peroxisome-derived H_2_O_2_ can trigger cysteine oxidation in multiple members of various protein families, including but not limited to antioxidant enzymes, constituents of the cytoskeleton, protein chaperones, annexins, and 14-3-3 and S100 proteins (Supplementary Tables S3-S5). Given that many of these proteins are at the crossroads of key cellular processes (Rubio et al., 2004; Méndez-Barbero et al., 2021; Wu et al., 2021), such as carbon metabolism, protein synthesis and folding, proteasome functioning, and calcium signaling, it can be expected that genetic-, age-, and environment-related changes in peroxisomal H_2_O_2_ metabolism also contribute to disease pathogenesis. This is perhaps best exemplified by the observations that 1) inherited catalase deficiency is associated with oxidative stress-related disorders, such as neoplasms, atherosclerosis, and diabetes (Góth and Nagy, 2013), and 2) a gain-of-function mutation (N237S) in acyl-CoA oxidase 1 (ACOX1), a peroxisomal enzyme that oxidizes very-long-chain fatty acids and produces H_2_O_2_ as a byproduct, causes oxidative damage associated with severe Schwann cell loss and myelination defects in humans (Chung et al., 2020). Here, it is also relevant to note that cells expressing very low levels of catalase (e.g., insulin-producing β-cells) are exceptionally vulnerable to excessive amounts of peroxisome-derived H_2_O_2_ that can be produced, for example, upon β-oxidation of long-chain saturated non-esterified fatty acids under lipotoxic conditions (Gehrmann et al., 2010; Elsner et al., 2011). However, when peroxisomes are not metabolically challenged, such cells are likely to cope with peroxisome-derived H_2_O_2_ through the activity of other H_2_O_2_-metabolizing enzymes (e.g., PRDXs) that display a much higher affinity for H_2_O_2_ than catalase, which displays a rather low affinity for H_2_O_2_ and mainly comes into play to limit excessive H_2_O_2_ accumulation (Rhee et al., 2018).

Our data also show that the IBD-SBP-YAP1C interactome differs considerably depending on the subcellular location of the YAP1C fusion protein (Figure 3), the duration of the oxidative insult (Figure 4), the H_2_O_2_ concentration (Figure 7A), and the source of the oxidant (Figure 8A). In addition, it is important to keep in mind that the capturing rate of sulfenylated proteins by specific YAP1C-fusion proteins will be influenced by other factors, such as 1) the local concentrations of the bait and target proteins, 2) the import efficiencies of these proteins into their organelle of destination, 3) the local H_2_O_2_ levels, and 4) the basal oxidation state of the redox-sensitive cysteines within the target protein. Notably, given that IBD-SBP-YAP1C captures only S-sulfenylated proteins, our findings do only shed light on the sulfenome, but not on the full sulfur redoxome. That is, proteins that are oxidatively modified via redox relay, and not through direct oxidation by H_2_O_2_, will remain undetected. Whether or not this underlies the absence of transcription factors in our target list, remains to be investigated. Another limitation of our approach is that the molecular mass of IBD-SBP-YAP1C is relatively high (30 kDa) (Supplementary Figure S1). Indeed, given that most cysteines are buried inside proteins or protein complexes (Poole, 2015), the corresponding sulfenic acids may not be accessible for IBD-SBP-YAP1C due to steric hindrance, thereby resulting in an underestimation of targets. Finally, another consideration is that IBD-SBP-YAP1C complexes can be reduced *in cellulo* (Figure 5C). Although this may seem surprising at first sight, it makes sense given that otherwise, IBD-SBP-YAP1C expression would cause severe toxicity due to irreversible trapping of thiol redox signaling proteins (e.g., TXN and PRDX1) that are already significantly sulfenylated under basal conditions. Nevertheless, our study platform hampers the possibility to determine potential effects induced by gene manipulation. In addition, it remains to be established how well this study model mimics the physiological situation.

To provide a deeper understanding of how intracellular cysteine redox networks are regulated in response to various H_2_O_2_ insults, it is crucial to monitor the sulfenylation dynamics at the level of individual proteins. From our experiments, it is evident that the cytosolic and mitochondrial sulfenome undergo time-dependent changes upon peroxisomal H_2_O_2_ production (Figure 4). Importantly, the latter observation provides direct molecular evidence for the previously established peroxisome-mitochondria redox connection (Lismont et al., 2019b). In addition, it is clear that the sulfenylation profiles can vary considerably between proteins (Supplementary Figure S7) and the type of H_2_O_2_ treatment (Figure 8). However, in general, members of the same protein family exhibit a similar response behavior (Figure 5 and Supplementary Figure S9). Also, the sulfenylation profiles of (partially) peroxisomal po-IBD-SBP-YAP1C interactors exhibited a bimodal pattern (Supplementary Figure S5). This heterogeneous character likely reflects a combination of factors, including 1) sulfenylation of multiple cysteine residues with distinct redox sensitivity, 2) the bimodal localization of po-IBD-SBP-YAP1C, and/or 3) time-dependent changes in local H_2_O_2_ levels. Finally, from the multiple lists of IBD-SBP-YAP1C interactors (Supplementary Table S1, Supplementary Tables S3-S7) and the sulfenylation profiles of individual targets (Figure 8), it can be concluded that findings obtained with external H_2_O_2_, even at concentrations as low as 10 μM, cannot simply be extrapolated to conditions in which this oxidant is produced endogenously (e.g., inside peroxisomes or the cytosol). Here, it is important to mention that we routinely included 10 mM 3-AT in our assay buffer to inhibit catalase activity. However, during the course of our experiments, we obtained immunoblot (Supplementary Figure S12A) and proteomics (Supplementary Table S3) data documenting that a 15 min D-Ala treatment of po-DD-DAO expressing Flp-In T-REx 293 cells in the absence of 3-AT yields a comparable number and subset of targets as the 2 min D-Ala treatment in the presence of 3-AT (Supplementary Figure S12B).

Another intriguing aspect of this study is that the employed sulfenome mining approach can apparently also provide more insight into the potential subcellular localization of the IBD-SBP-YAP1C interactors. One example that we studied in more detail is PRDX1, a predominantly cytosolic and nuclear protein that functions as an antioxidant enzyme and protein chaperone under oxidative distress conditions (Rhee and Woo, 2020). Given that the amount of PRDX1 captured by po-IBD-SBP-YAP1C was much higher than what one would expect from the estimated amount of the mislocalized bait protein (Supplementary Figure S7), our data suggested that this protein was also present in peroxisomes, a finding strengthened by subcellular fractionation studies on HEK-293 cells and rat liver (Supplementary Figure S6). Note that, in contrast to mitochondria, typical 2-Cys PRDXs and TXNs have not yet been identified in mammalian peroxisomes (Lismont et al., 2015). In this context, it is worth noting that, at least according to our sulfenome mining data (Supplementary Figure S7), also PRDX2 and TXN are partially located inside peroxisomes. However, it is not the scope of this work to dig into the subcellular localization of all these and other targets of peroxisome-derived H_2_O_2_, but this information may stimulate research efforts on how peroxisomes regulate their intraorganellar redox state.

Finally, some interesting open questions that arise from this work but need to be addressed in more detail in future studies include: How do sulfenylation (and subsequent oxidative modifications) of specific cysteine residues within target proteins of peroxisome-derived H_2_O_2_ modulate their function and signaling properties? Why are some peroxisomal proteins (e.g., HSD17B4) more efficiently enriched by c-IBD-SBP-YAP1C than by po-IBD-SBP-YAP1C, and what is the molecular explanation for this observation and the finding that only very few *bona fide* peroxisomal proteins are captured by po-IBD-SBP-YAP1C? Why are transcription factors lacking in our sulfenome lists? Are non-peroxisomal proteins that are captured by po-IBD-SBP-YAP1C in aberrantly high levels partially localized in peroxisomes, and if so how are they targeted to this organelle? Are the mitochondrial proteins captured by mt-IBD-SBP-YAP1C directly sulfenylated by H_2_O_2_ that diffuses out of peroxisomes or does peroxisome-derived H_2_O_2_ function as a second messenger that activates ROS-induced ROS release in neighboring mitochondria? What are the key players and molecular mechanisms that facilitate H_2_O_2_ permeation across the peroxisomal membrane?

In conclusion, we provide a first snapshot view of the HEK-293 sulfenome in response to peroxisome-derived or externally added H_2_O_2_. Our approach distinguishes between targets in different cellular locations and allows, under ideal conditions, to identify the transiently sulfenylated cysteines within target proteins. The outcome of these experiments revealed a previously unexplored potential role for peroxisomes in diverse redox-regulated processes, including but not limited to cytoskeletal remodeling, calcium signaling, and protein synthesis and turnover. As such, this study opens new perspectives for research on how perturbations in peroxisomal H_2_O_2_ metabolism may contribute to the initiation and development of redox stress-related diseases.

## Data Availability

The datasets presented in this study can be found in the PRoteomics IDEntifications (PRIDE) database (https://www.ebi.ac.uk/pride/) under the PX identifier PXD030782.

## Abbreviations

3-AT, 3-amino-1,2,4-triazole; c-, cytosolic; DAO, D-amino acid oxidase; DD, destabilization domain; DTT, dithiothreitol; DOX, doxycycline; DPBS, Dulbecco’s phosphate-buffered saline; EMSA, electrophoretic mobility shift assay; IBD, IgG-binding domain; MS, mass spectrometry; mt-, mitochondrial; NEM, N-ethylmaleimide; Ox-PPP, oxidative branch of the pentose phosphate pathway; PEP, posterior error probability; po-, peroxisomal; PRDX, peroxiredoxin; PSM, peptide spectral match; PTS1, C-terminal peroxisomal targeting signal; SBP, streptavidin-binding protein; YAP1C, C-terminal region of yeast AP-1-like transcription factor. Given the extensive list of proteins identified in the sulfenome mining experiments, we refer the reader to the UniProtKB database for details on the protein acronyms used in the manuscript.
