# Supplementary material for: Peroxisome-Derived Hydrogen Peroxide Modulates the Sulfenylation Profiles of Key Redox Signaling Proteins in Flp-In T-REx 293 Cells
Source: Front Cell Dev Biol. 2022 Apr 26;10:888873. doi: 10.3389/fcell.2022.888873 (PMC9086853; doi:10.3389/fcell.2022.888873)
Supplement: Supplementary file 1 [file DataSheet2.PDF]

TABLE S1. Heat map summarizing the raw abundances of proteins trapped by po-, mt-, or c-IBD-SBP-YAP1C in response to treatment of Flp-In T-REx 293 cells with 1 mM H_2O_2 for 10 min. DTT eluates of the experiment shown in Fig. S2 were processed for LC-MS/MS analysis. After validation, proteins enriched 2.5-fold or more in at least one of the H_2O_2-treated conditions were retrieved, named according to their UniProtKB gene name, and sorted in alphabetical order. The primary subcellular localizations of the proteins (according to the same database) are indicated between brackets. The values represent raw abundances and are color-coded (with white and blue being low and high, respectively). C, cytosol; CS, cytoskeleton; EC, extracellular; EE, early endosome; ER, endoplasmic reticulum; GA, Golgi apparatus; LY, lysosome; MT, mitochondria; NU, nucleus; PM, plasma membrane; PO, peroxisome.

Table S1

Raw abundance by protein and 1 mM H_2O_2 treatment (10 min); colour scale 0 to 1×10^{7} (0, 2×10^{6}, 4×10^{6}, 6×10^{6}, 8×10^{6}, 1×10^{7}).

Protein	po-IBD-SBP-YAP 0	po-IBD-SBP-YAP 1	mt-IBD-SBP-YAP 0	mt-IBD-SBP-YAP 1	c-IBD-SBP-YAP 0	c-IBD-SBP-YAP 1
ACOX1 (PO)	1.37×10^{5}	2.00×10^{6}	1.48×10^{5}	1.18×10^{5}	1.30×10^{5}	2.74×10^{5}
BOLA2B (C/NU)	8.38×10^{3}	1.24×10^{5}	2.74×10^{4}	9.07×10^{4}	3.05×10^{4}	2.11×10^{5}
CDK4 (C/NU)	5.41×10^{3}	5.80×10^{4}	2.86×10^{3}	2.87×10^{4}	1.02×10^{4}	3.12×10^{5}
COL1A2 (ER)	8.64×10^{3}	6.19×10^{3}	5.52×10^{3}	1.75×10^{4}	3.73×10^{3}	4.35×10^{4}
COX17 (C/MT)	0	1.16×10^{5}	0	6.42×10^{4}	0	8.56×10^{4}
CSTB (C/NU)	1.93×10^{5}	1.24×10^{6}	2.46×10^{5}	9.35×10^{5}	4.31×10^{5}	2.02×10^{6}
DCTPP1 (C/NU/MT)	3.62×10^{3}	5.95×10^{4}	1.34×10^{3}	2.94×10^{4}	1.19×10^{4}	1.56×10^{5}
DNPEP (C)	1.61×10^{4}	9.01×10^{4}	2.83×10^{4}	5.81×10^{4}	3.30×10^{4}	1.16×10^{6}
ECI1 (MT)	2.08×10^{3}	1.86×10^{4}	8.69×10^{3}	1.89×10^{5}	1.99×10^{4}	2.13×10^{4}
FLNA (CS)	0	4.09×10^{3}	1.83×10^{4}	1.19×10^{4}	4.38×10^{3}	3.12×10^{4}
GSR (C/MT)	1.17×10^{5}	1.16×10^{6}	1.56×10^{5}	9.75×10^{5}	1.44×10^{5}	1.35×10^{6}
HBA2 (C/EC)	1.07×10^{5}	1.26×10^{5}	5.70×10^{4}	1.24×10^{6}	5.36×10^{4}	7.18×10^{6}
HBB (C/EC)	2.54×10^{5}	3.05×10^{5}	4.17×10^{5}	1.88×10^{6}	3.20×10^{5}	6.68×10^{6}
HPRT1 (C)	1.87×10^{4}	7.30×10^{5}	8.86×10^{4}	6.85×10^{5}	1.30×10^{5}	1.89×10^{6}
HSD17B10 (MT)	2.05×10^{4}	2.41×10^{5}	8.80×10^{4}	1.97×10^{6}	5.51×10^{4}	1.78×10^{5}
HSPA9 (MT)	6.12×10^{5}	1.21×10^{6}	7.99×10^{5}	2.01×10^{6}	1.19×10^{6}	1.03×10^{6}
ISYNA1 (C)	1.19×10^{3}	4.38×10^{4}	4.78×10^{2}	8.95×10^{4}	2.11×10^{3}	1.42×10^{5}
LDHB (C/MT)	1.05×10^{5}	2.97×10^{5}	1.83×10^{5}	1.01×10^{5}	6.13×10^{5}	8.96×10^{4}
LMNA (NU)	3.32×10^{5}	1.21×10^{5}	8.93×10^{5}	2.34×10^{5}	1.46×10^{5}	4.10×10^{5}
LY6D (PM)	2.99×10^{4}	3.62×10^{4}	4.99×10^{4}	4.79×10^{4}	2.66×10^{4}	7.30×10^{4}
MARCKSL1(CS/PM)	0	2.21×10^{4}	1.60×10^{3}	3.83×10^{3}	2.68×10^{4}	7.52×10^{3}
MDH1 (C)	2.31×10^{4}	1.19×10^{5}	3.58×10^{4}	9.55×10^{3}	3.48×10^{4}	1.15×10^{4}
ME2 (MT)	0	6.01×10^{2}	0	1.09×10^{5}	9.16×10^{2}	0
NPEPPS (C/NU)	6.47×10^{4}	1.27×10^{5}	1.55×10^{5}	2.46×10^{5}	9.84×10^{4}	5.65×10^{5}
NUDC (CS/NU)	2.85×10^{4}	9.51×10^{4}	2.58×10^{4}	1.05×10^{5}	7.38×10^{4}	4.91×10^{5}
PKP1 (NU)	1.23×10^{6}	5.69×10^{5}	1.26×10^{6}	1.23×10^{6}	6.18×10^{5}	1.67×10^{6}
PLS3 (C)	3.57×10^{4}	1.11×10^{5}	3.82×10^{4}	8.61×10^{4}	4.92×10^{4}	3.08×10^{5}
PRDX1 (C)	2.60×10^{6}	8.75×10^{6}	3.82×10^{6}	9.32×10^{6}	2.80×10^{6}	2.58×10^{7}
PRDX2 (C)	1.28×10^{6}	1.44×10^{6}	1.85×10^{6}	2.36×10^{6}	1.30×10^{6}	4.28×10^{6}
PRDX3 (MT/C/EE)	3.09×10^{4}	3.42×10^{5}	1.33×10^{5}	1.52×10^{6}	7.22×10^{4}	9.35×10^{4}
PRDX4 (C/ER)	1.79×10^{4}	6.47×10^{4}	3.19×10^{4}	5.49×10^{4}	5.70×10^{4}	8.75×10^{4}
PRDX5 (C/NU/MT/PO)	2.19×10^{3}	4.48×10^{4}	4.33×10^{3}	5.38×10^{4}	5.15×10^{3}	3.34×10^{4}
PRDX6 (C/LY)	3.15×10^{4}	4.41×10^{5}	6.49×10^{4}	4.79×10^{5}	1.93×10^{5}	1.22×10^{6}
PRMT5 (C/NU/GA)	1.07×10^{4}	9.49×10^{4}	1.03×10^{4}	7.96×10^{4}	8.98×10^{3}	2.27×10^{5}
RPL13 (C)	1.37×10^{4}	3.52×10^{4}	1.52×10^{4}	4.31×10^{3}	8.62×10^{4}	1.44×10^{4}
SERPINB1 (C/ES/LY/EC)	5.24×10^{2}	3.13×10^{4}	0	1.14×10^{4}	3.39×10^{3}	3.85×10^{4}
SET (C/NU)	3.72×10^{3}	1.63×10^{4}	2.51×10^{4}	8.70×10^{3}	5.27×10^{4}	2.12×10^{3}
SKP1 (C/NU)	4.08×10^{4}	1.84×10^{6}	2.53×10^{4}	1.01×10^{6}	2.85×10^{4}	2.83×10^{6}
TPM3 (CS)	7.75×10^{3}	3.72×10^{4}	1.34×10^{4}	6.36×10^{4}	1.58×10^{4}	2.31×10^{5}
TPM4 (CS)	0	2.67×10^{4}	0	0	0	3.76×10^{4}
TUBB1 (CS)	2.71×10^{3}	3.02×10^{3}	3.07×10^{3}	3.56×10^{3}	2.30×10^{4}	2.60×10^{5}
TXN (C/NU/EC)	2.14×10^{6}	3.38×10^{6}	3.65×10^{6}	3.83×10^{6}	1.85×10^{6}	5.88×10^{6}
TXNRD2 (MT)	3.65×10^{4}	7.55×10^{4}	4.08×10^{4}	3.16×10^{6}	3.72×10^{4}	4.39×10^{4}
UCHL3 (C)	6.47×10^{3}	3.39×10^{4}	1.14×10^{4}	4.26×10^{4}	1.46×10^{4}	3.94×10^{5}
WDR77 (C/NU)	2.57×10^{4}	1.24×10^{5}	4.11×10^{4}	1.67×10^{5}	2.98×10^{4}	4.02×10^{5}
YWHAB (C)	7.68×10^{3}	2.75×10^{4}	2.49×10^{4}	2.36×10^{4}	4.65×10^{4}	4.02×10^{4}
YWHAE (C/NU)	2.04×10^{4}	8.53×10^{4}	3.95×10^{4}	6.55×10^{4}	8.74×10^{4}	1.76×10^{5}
YWHAQ (C)	1.49×10^{4}	2.53×10^{5}	3.05×10^{4}	1.26×10^{5}	7.34×10^{4}	2.76×10^{5}

Column labels: 0 / 1 = 1 mM H_2O_2 (10 min); colour scale: raw abundance

TABLE S2. Heat map summarizing the percentage distribution of proteins trapped by po-, mt-, or c-IBD-SBP-YAP1C upon treatment of Flp-In T-REx 293 cells with 1 mM H_2O_2 for 10 min. The percentage distributions of the raw abundances of proteins trapped by po-, mt, or c-IBD-SBP-YAP1C (denoted as po-YAP, mt-YAP, and c-YAP, respectively) after H_2O_2 treatment were calculated and presented in a heat map (with white and blue intensities representing low and high percentages of complex formation). The UniProtKB gene name was used to name the interactors, and their primary subcellular localizations (according to the same database) are indicated between brackets. C, cytosol; CS, cytoskeleton; EC, extracellular; EE, early endosome; ER, endoplasmic reticulum; GA, Golgi apparatus; LY, lysosome; MT, mitochondria; NU, nucleus; PM, plasma membrane; PO, peroxisome.

Table S2

Protein	po-YAP	mt-YAP	c-YAP
ACOX1 (PO)	84	5	11
BOLA2B (C/NU)	29	21	50
CDK4 (C/NU)	15	7	78
COL1A2 (ER)	9	26	65
COX17 (C/MT)	44	24	32
CSTB (C/NU)	30	22	48
DCTPP1 (C/NU/MT)	24	12	64
DNPEP (C)	7	4	89
ECI1 (MT)	8	83	9
FLNA (CS)	9	25	66
GSR (C/MT)	33	28	39
HBA2 (C/EC)	1	14	84
HBB (C/EC)	3	21	75
HPRT1 (C)	22	21	57
HSD17B10 (MT)	10	83	7
HSPA9 (MT)	28	47	24
ISYNA1 (C)	16	33	52
LDHB (C/MT)	61	21	18
LMNA (NU)	16	31	54
LY6D (PM)	23	30	46
MARCKSL1(CS/PM)	66	11	23
MDH1 (C)	85	7	8
ME2 (MT)	1	99	0
NPEPPS (C/NU)	14	26	60
NUDC (CS/NU)	14	15	71
PKP1 (NU)	16	35	48
PLS3 (C)	22	17	61
PRDX1 (C)	20	21	59
PRDX2 (C)	18	29	53
PRDX3 (MT/C/EE)	17	78	5
PRDX4 (C/ER)	31	27	42
PRDX5 (C/NU/MT/PO)	34	41	25
PRDX6 (C/LY)	21	22	57
PRMT5 (C/NU/GA)	24	20	57
RPL13 (C)	65	8	27
SERPINB1 (C/ES/LY/EC)	39	14	47
SET (C/NU)	60	32	8
SKP1 (C/NU)	32	18	50
TPM3 (CS)	11	19	70
TPM4 (CS)	42	0	58
TUBB1 (CS)	1	1	98
TXN (C/NU/EC)	26	29	45
TXNRD2 (MT)	2	97	1
UCHL3 (C)	7	9	84
WDR77 (C/NU)	18	24	58
YWHAB (C)	30	26	44
YWHAE (C/NU)	26	20	54
YWHAQ (C)	39	19	42

percentage distribution (0–100)

1 mM H_2O_2 (10 min)

TABLE S3. Heat map summarizing the raw abundances of proteins trapped by c-IBD-SBP-YAP1C in response to treatment of po-DD-DAO-expressing Flp-In T-REx 293 cells with D-Ala. DTT eluates of the experiment shown in Fig. S4A were processed for LC-MS/MS analysis. After validation, proteins enriched 2.5-fold or more in at least one of the time points (0, 2, 5, 15, 30, and 60 min) after H_2O_2 exposure were retrieved, named according to their UniProtKB gene name, and sorted in alphabetical order. The primary subcellular localizations of the proteins (according to the same database) are indicated between brackets. The values represent raw abundances and are color-coded (with white and blue being low and high, respectively). C, cytosol; CS, cytoskeleton; EC, extracellular; EE, early endosome; ER, endoplasmic reticulum; GA, Golgi apparatus; LY, lysosome; MT, mitochondria; NU, nucleus; PM, plasma membrane; PO, peroxisome.

Table S3 (page 1)

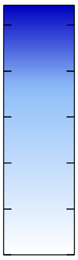

	po-H_2O_2 / c-YAP1C 0	2	5	15	30	60	- 3-AT 15
ACADVL (MT)	6.81×10^{2}	1.02×10^{3}	5.81×10^{4}	5.93×10^{4}	3.68×10^{2}	1.70×10^{3}	1.11×10^{3}
ACP3 (NU/C/PM/LY/EC)	0	1.63×10^{2}	3.95×10^{4}	2.15×10^{3}	1.52×10^{2}	0	0
ACTB (NU/CS)	1.08×10^{6}	1.36×10^{6}	1.08×10^{7}	1.45×10^{7}	2.79×10^{6}	2.03×10^{6}	7.66×10^{5}
ACTC1 (CS)	1.24×10^{4}	1.61×10^{4}	1.27×10^{5}	2.15×10^{5}	4.79×10^{4}	1.00×10^{4}	1.53×10^{4}
ACTN1 (C/PM)	2.86×10^{2}	3.14×10^{2}	1.75×10^{4}	2.17×10^{4}	5.69×10^{2}	3.82×10^{3}	3.40×10^{2}
ACTN4 (C/NU/CS)	2.79×10^{4}	6.59×10^{4}	1.74×10^{6}	1.71×10^{6}	3.52×10^{4}	6.10×10^{4}	3.50×10^{4}
ACTR1A (CS)	0	1.84×10^{3}	1.58×10^{5}	9.38×10^{4}	1.23×10^{4}	1.82×10^{3}	9.75×10^{2}
ACTR2 (NU/CS)	2.49×10^{4}	9.61×10^{3}	3.51×10^{5}	3.20×10^{5}	2.52×10^{4}	1.34×10^{4}	6.28×10^{3}
ACTR3 (NU/C/CS)	1.62×10^{4}	2.37×10^{4}	3.66×10^{5}	2.70×10^{5}	1.30×10^{4}	3.83×10^{4}	8.62×10^{3}
AHCY (C)	2.48×10^{4}	3.14×10^{4}	2.09×10^{5}	7.38×10^{4}	4.42×10^{4}	7.06×10^{4}	2.27×10^{4}
AHNAK (NU)	2.43×10^{5}	2.04×10^{5}	2.18×10^{6}	5.02×10^{6}	3.15×10^{5}	1.00×10^{5}	2.15×10^{5}
AHNAK2 (NU)	1.67×10^{3}	3.74×10^{3}	4.34×10^{4}	2.20×10^{5}	2.48×10^{3}	2.06×10^{3}	1.68×10^{3}
AIMP1 (C/NU/ER/GA/PM)	6.08×10^{2}	1.68×10^{2}	2.13×10^{4}	1.77×10^{4}	5.07×10^{2}	1.32×10^{2}	6.98×10^{1}
AKR1B10 (LY/EC)	1.26×10^{3}	4.68×10^{3}	1.16×10^{5}	1.13×10^{4}	2.63×10^{3}	5.56×10^{3}	6.14×10^{3}
ALDH2 (MT)	3.63×10^{3}	1.48×10^{4}	1.95×10^{5}	3.52×10^{5}	8.88×10^{3}	4.63×10^{3}	4.95×10^{3}
ALDH3A1 (C)	1.23×10^{2}	7.65×10^{2}	6.08×10^{4}	1.78×10^{5}	2.89×10^{3}	3.70×10^{2}	3.17×10^{2}
ALDH3A2 (ER)	5.32×10^{3}	1.49×10^{4}	1.52×10^{5}	1.65×10^{5}	5.87×10^{3}	2.07×10^{4}	6.67×10^{3}
ALDH9A1 (C)	3.58×10^{4}	3.46×10^{4}	3.39×10^{5}	1.68×10^{5}	4.00×10^{4}	3.30×10^{4}	1.34×10^{4}
ALDOA (C/NU)	1.63×10^{5}	2.82×10^{5}	2.77×10^{6}	2.33×10^{6}	2.53×10^{5}	2.90×10^{5}	1.51×10^{5}
ALDOC (C/CS/EC)	6.40×10^{3}	1.94×10^{4}	5.20×10^{5}	2.64×10^{5}	1.10×10^{4}	2.03×10^{4}	9.17×10^{3}
ALOX12B (C)	2.43×10^{5}	1.45×10^{5}	1.41×10^{6}	3.91×10^{5}	1.62×10^{5}	6.05×10^{4}	1.71×10^{5}
ALOXE3 (C)	9.33×10^{3}	1.27×10^{4}	9.55×10^{4}	2.91×10^{4}	1.78×10^{4}	6.32×10^{3}	9.18×10^{3}
ALYREF (C/NU)	8.37×10^{4}	1.74×10^{5}	9.43×10^{4}	1.54×10^{5}	1.76×10^{5}	2.47×10^{5}	2.14×10^{5}
ANXA1 (C/NU/PM/EC)	3.41×10^{5}	1.36×10^{5}	9.10×10^{5}	4.02×10^{6}	6.44×10^{5}	5.03×10^{4}	1.68×10^{5}
ANXA2 (PM/EC)	2.17×10^{6}	1.49×10^{6}	1.15×10^{7}	9.98×10^{6}	2.25×10^{6}	2.15×10^{6}	1.23×10^{6}
ANXA4 (C/NU/PM/EC)	2.24×10^{3}	5.62×10^{3}	3.29×10^{5}	2.45×10^{5}	4.75×10^{3}	4.74×10^{3}	8.19×10^{3}
ANXA5 (C/EC)	1.61×10^{4}	1.52×10^{4}	1.01×10^{5}	1.30×10^{5}	3.52×10^{4}	7.02×10^{4}	3.91×10^{4}
ANXA8 (PM, ES)	2.85×10^{2}	4.01×10^{2}	2.65×10^{5}	2.86×10^{5}	1.90×10^{3}	1.15×10^{3}	1.44×10^{3}
ARF3 (GA/C)	1.20×10^{4}	4.30×10^{4}	4.43×10^{4}	7.65×10^{4}	5.61×10^{3}	1.59×10^{4}	8.06×10^{3}
ARF6 (C/PM/GA/ES)	1.53×10^{3}	5.10×10^{2}	2.18×10^{4}	4.64×10^{4}	0	7.06×10^{2}	8.17×10^{2}
ARG1 (C)	1.76×10^{6}	1.23×10^{6}	8.02×10^{6}	7.51×10^{5}	1.79×10^{6}	5.27×10^{5}	1.22×10^{6}
ARHGDIA (C)	6.48×10^{3}	1.63×10^{4}	1.62×10^{5}	1.49×10^{5}	7.18×10^{3}	1.43×10^{5}	1.66×10^{4}
ARPC3 (NU/CS)	2.06×10^{3}	9.92×10^{2}	1.28×10^{5}	1.28×10^{5}	6.54×10^{2}	9.61×10^{2}	3.61×10^{2}
ASAH1 (C/LY/EC)	1.80×10^{4}	1.48×10^{4}	9.49×10^{4}	3.93×10^{4}	2.80×10^{4}	5.34×10^{3}	2.78×10^{4}
ASPRV1 (C/NU)	5.03×10^{0}	5.20×10^{2}	1.67×10^{5}	1.24×10^{4}	8.28×10^{2}	4.48×10^{2}	1.68×10^{2}
ATP2A2 (ER)	1.07×10^{3}	3.88×10^{2}	5.03×10^{3}	4.27×10^{4}	1.37×10^{2}	1.70×10^{4}	3.61×10^{2}
ATP5F1A (MT)	8.85×10^{4}	1.15×10^{5}	4.24×10^{5}	1.15×10^{6}	1.42×10^{5}	1.37×10^{5}	1.36×10^{5}
ATP5F1B (MT)	2.13×10^{5}	3.00×10^{5}	5.90×10^{5}	1.63×10^{6}	2.78×10^{5}	2.83×10^{5}	2.71×10^{5}
ATP5F1C (MT)	1.05×10^{3}	8.45×10^{3}	1.15×10^{4}	5.26×10^{4}	7.16×10^{3}	8.41×10^{3}	3.30×10^{3}
ATP5PB (MT)	1.11×10^{3}	4.76×10^{2}	7.05×10^{3}	9.63×10^{4}	7.56×10^{2}	4.35×10^{3}	1.02×10^{3}
ATP5PD (MT)	0	0	3.64×10^{2}	7.72×10^{3}	0	0	0
ATP5PO (MT)	7.67×10^{3}	2.33×10^{4}	1.22×10^{4}	1.98×10^{5}	1.64×10^{4}	3.48×10^{4}	2.80×10^{4}
ATP6V1A (C)	2.80×10^{3}	1.96×10^{3}	1.99×10^{5}	1.68×10^{5}	1.89×10^{3}	5.09×10^{3}	2.79×10^{3}
ATP6V1G1 (PM)	3.75×10^{3}	9.53×10^{2}	2.70×10^{4}	2.23×10^{4}	8.53×10^{3}	1.47×10^{3}	1.84×10^{2}
BLMH (C)	3.19×10^{5}	3.71×10^{5}	3.91×10^{6}	5.93×10^{5}	7.88×10^{5}	1.86×10^{5}	3.41×10^{5}
CA2 (C/PM)	2.14×10^{3}	1.47×10^{3}	3.80×10^{4}	1.58×10^{5}	6.14×10^{2}	5.86×10^{2}	3.27×10^{2}
CALM2 (C)	2.81×10^{4}	7.90×10^{4}	7.40×10^{5}	2.08×10^{5}	2.29×10^{4}	2.54×10^{4}	1.65×10^{4}
CALR (C/ER/LY/EC)	4.56×10^{3}	1.71×10^{4}	1.11×10^{5}	8.34×10^{4}	2.65×10^{4}	2.61×10^{4}	1.68×10^{4}
CAND1 (C/NU)	4.24×10^{2}	1.66×10^{2}	3.33×10^{3}	3.53×10^{4}	6.82×10^{2}	1.50×10^{2}	1.55×10^{2}
CANX (ER)	3.72×10^{3}	1.05×10^{4}	5.26×10^{4}	1.26×10^{5}	1.66×10^{4}	3.32×10^{4}	1.38×10^{4}

Table S3
(page 2)

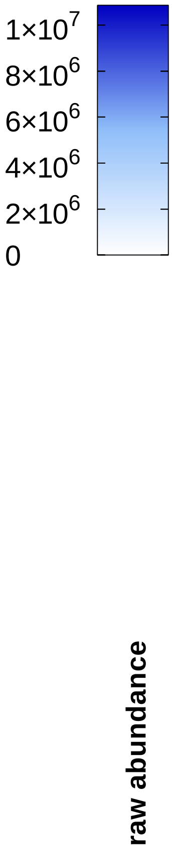

	po-H_2O_2 / c-YAP1C						
	0	2	5	15	30	60	- 3-AT 15
CAP1 (PM)	4.51×10^{3}	2.42×10^{3}	1.70×10^{5}	3.56×10^{5}	1.12×10^{3}	5.58×10^{3}	1.31×10^{4}
CAPG (C/NU)	2.95×10^{3}	3.71×10^{3}	2.93×10^{5}	3.25×10^{5}	8.72×10^{2}	2.94×10^{4}	6.08×10^{2}
CAPN1 (C/PM)	6.55×10^{4}	5.73×10^{4}	5.54×10^{5}	3.03×10^{5}	5.37×10^{4}	3.62×10^{4}	3.94×10^{4}
CAPZA1 (CS)	2.30×10^{4}	2.62×10^{4}	7.35×10^{5}	3.61×10^{5}	2.51×10^{4}	2.80×10^{4}	9.02×10^{3}
CAPZB (CS)	6.74×10^{4}	5.01×10^{4}	5.04×10^{5}	2.81×10^{5}	3.85×10^{4}	6.45×10^{4}	2.90×10^{4}
CARD18 (???)	6.04×10^{2}	3.25×10^{2}	4.03×10^{4}	6.92×10^{3}	0	0	0
CASP14 (C/NU)	8.11×10^{5}	7.98×10^{5}	7.17×10^{6}	1.23×10^{6}	1.11×10^{6}	2.46×10^{5}	7.82×10^{5}
CAT (PO)	8.51×10^{5}	5.04×10^{5}	3.49×10^{6}	4.66×10^{5}	1.26×10^{6}	2.65×10^{5}	6.15×10^{5}
CBR1 (C)	1.65×10^{3}	5.15×10^{3}	1.89×10^{5}	2.38×10^{5}	1.12×10^{4}	1.62×10^{4}	4.07×10^{3}
CBX1 (NU)	9.35×10^{3}	2.10×10^{4}	3.41×10^{4}	3.54×10^{4}	1.79×10^{4}	1.86×10^{4}	7.72×10^{3}
CCT2 (C)	6.28×10^{3}	5.20×10^{3}	2.60×10^{4}	1.19×10^{5}	5.52×10^{3}	8.19×10^{3}	5.35×10^{3}
CCT4 (C/CS)	1.46×10^{3}	9.07×10^{4}	6.46×10^{4}	1.66×10^{5}	3.12×10^{4}	2.50×10^{4}	7.66×10^{3}
CCT5 (C/CS)	3.66×10^{3}	3.54×10^{3}	3.49×10^{4}	3.71×10^{4}	3.14×10^{3}	3.72×10^{3}	1.83×10^{3}
CCT6A (C)	1.09×10^{2}	9.23×10^{2}	2.84×10^{4}	1.00×10^{5}	0	1.74×10^{3}	1.09×10^{2}
CCT8 (C/CS)	6.78×10^{3}	4.40×10^{3}	1.27×10^{5}	2.06×10^{5}	2.62×10^{3}	9.72×10^{3}	2.26×10^{3}
CD44 (PM)	0	0	0	4.88×10^{4}	0	0	0
CDK4 (C/NU)	6.53×10^{3}	1.28×10^{3}	2.85×10^{4}	9.11×10^{4}	4.26×10^{4}	2.47×10^{5}	4.85×10^{3}
CDKN2A (C/NU)	4.87×10^{2}	9.10×10^{2}	3.03×10^{3}	1.73×10^{4}	4.67×10^{4}	2.48×10^{5}	9.55×10^{3}
CFL1 (C/NU/CS)	9.27×10^{4}	2.98×10^{5}	5.77×10^{5}	1.24×10^{6}	1.69×10^{5}	4.07×10^{5}	2.22×10^{5}
CHMP4B (C/NU/ES)	0	6.73×10^{2}	2.03×10^{4}	2.89×10^{4}	0	2.00×10^{2}	1.75×10^{2}
CHTOP (NU)	3.18×10^{4}	5.16×10^{4}	8.04×10^{4}	9.06×10^{4}	1.30×10^{5}	1.08×10^{5}	1.54×10^{5}
CKB (C)	8.22×10^{4}	8.94×10^{4}	1.48×10^{5}	2.68×10^{5}	3.21×10^{5}	3.66×10^{5}	3.62×10^{5}
CKMT1A (MT)	8.28×10^{3}	7.21×10^{3}	7.90×10^{4}	1.22×10^{5}	1.55×10^{4}	6.21×10^{3}	9.85×10^{3}
CLIC1 (C/NU/PM)	4.10×10^{4}	3.78×10^{4}	1.50×10^{5}	4.40×10^{5}	1.92×10^{4}	3.78×10^{4}	1.58×10^{4}
CLTC (CS/VS)	3.64×10^{4}	2.32×10^{4}	4.40×10^{5}	1.06×10^{6}	1.74×10^{4}	3.00×10^{4}	2.23×10^{4}
CMPK1 (C/NU)	3.25×10^{2}	1.94×10^{3}	7.58×10^{4}	2.89×10^{4}	7.59×10^{3}	7.55×10^{2}	1.81×10^{3}
COX7A2 (MT)	1.03×10^{4}	1.82×10^{4}	3.00×10^{4}	6.15×10^{4}	2.22×10^{4}	1.56×10^{4}	1.70×10^{4}
CRYZL1 (C)	1.72×10^{2}	0	2.61×10^{3}	6.10×10^{2}	7.77×10^{1}	2.01×10^{4}	7.73×10^{0}
CS (MT)	1.58×10^{4}	4.27×10^{3}	1.71×10^{4}	1.54×10^{5}	1.02×10^{4}	8.86×10^{3}	9.17×10^{3}
CSNK1A1 (C/NU/CS)	5.46×10^{2}	1.81×10^{3}	2.32×10^{4}	1.09×10^{5}	7.43×10^{3}	4.19×10^{3}	1.61×10^{3}
CSTA (C)	1.17×10^{6}	2.92×10^{5}	3.26×10^{6}	3.80×10^{5}	1.19×10^{6}	2.51×10^{5}	1.20×10^{6}
CSTB (C/NU)	1.14×10^{5}	9.89×10^{4}	4.32×10^{5}	9.56×10^{5}	4.05×10^{5}	8.33×10^{5}	1.35×10^{5}
CTNNA1 (CS/PM)	2.22×10^{3}	1.98×10^{3}	2.48×10^{4}	6.40×10^{4}	1.03×10^{3}	6.92×10^{2}	1.02×10^{3}
CTNND1 (C/NU/PM)	8.14×10^{1}	0	8.11×10^{2}	1.74×10^{4}	0	0	7.17×10^{1}
CTSA (LY)	3.24×10^{4}	2.01×10^{4}	2.79×10^{5}	2.15×10^{4}	3.76×10^{4}	7.44×10^{3}	1.62×10^{4}
CTSD (LY/EC)	1.77×10^{5}	2.99×10^{5}	4.07×10^{6}	5.01×10^{5}	3.80×10^{5}	8.51×10^{4}	2.18×10^{5}
CYB5R3 (C/MT/ER)	1.16×10^{4}	3.85×10^{3}	1.32×10^{4}	6.85×10^{4}	6.11×10^{3}	1.98×10^{4}	3.43×10^{3}
CYCS (MT)	5.57×10^{3}	1.22×10^{4}	1.96×10^{4}	2.42×10^{4}	3.32×10^{2}	4.55×10^{2}	5.60×10^{2}
CYFIP2 (C/NU)	7.98×10^{2}	8.28×10^{2}	1.80×10^{4}	2.01×10^{4}	1.39×10^{2}	1.95×10^{2}	2.19×10^{2}
DCTN1 (C/NU/CS)	0	6.59×10^{2}	3.79×10^{3}	1.58×10^{4}	0	1.66×10^{2}	2.04×10^{1}
DCTN2 (CS)	0	0	0	1.61×10^{4}	5.12×10^{2}	1.89×10^{2}	0
DCTPP1 (C/NU/MT)	2.07×10^{2}	2.00×10^{3}	1.60×10^{3}	3.64×10^{3}	3.81×10^{3}	2.88×10^{4}	7.49×10^{2}
DDOST (ER)	9.14×10^{3}	7.99×10^{3}	9.11×10^{4}	6.61×10^{4}	9.83×10^{3}	1.23×10^{4}	1.42×10^{4}
DDX39B (C/NU)	3.84×10^{3}	1.06×10^{4}	5.73×10^{4}	1.21×10^{5}	1.11×10^{4}	5.69×10^{4}	9.38×10^{3}
DDX3X (C/CS/NU/PM)	3.64×10^{3}	2.34×10^{3}	1.31×10^{5}	2.41×10^{5}	5.39×10^{3}	4.81×10^{3}	1.83×10^{3}
DHX9 (C/NU/CS)	7.35×10^{2}	1.02×10^{3}	3.17×10^{4}	4.27×10^{4}	1.09×10^{4}	5.66×10^{1}	5.23×10^{3}
DLD (NU/MT)	4.57×10^{4}	7.42×10^{4}	2.99×10^{5}	1.09×10^{5}	1.21×10^{5}	7.76×10^{4}	3.48×10^{4}
DNAJB1 (C/NU)	0	0	0	1.61×10^{4}	0	0	0
DSC1 (PM)	2.69×10^{6}	1.78×10^{6}	1.58×10^{7}	2.62×10^{6}	3.24×10^{6}	7.79×10^{5}	1.48×10^{6}
DSC3 (PM)	4.35×10^{5}	2.36×10^{5}	1.70×10^{6}	1.20×10^{6}	3.38×10^{5}	1.22×10^{5}	1.94×10^{5}

Table S3 (page 3)

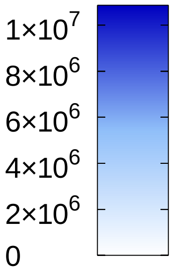

	po-H_2O_2 / c-YAP1C						- 3-AT
	0	2	5	15	30	60	15
DSG1 (PM)	1.01×10^{7}	6.25×10^{6}	5.12×10^{7}	9.68×10^{6}	1.04×10^{7}	3.45×10^{6}	5.75×10^{6}
DSG3 (PM)	3.52×10^{2}	7.61×10^{2}	2.15×10^{3}	1.73×10^{5}	4.16×10^{2}	0	6.60×10^{1}
DSP (CS/PM)	1.66×10^{7}	8.00×10^{6}	7.84×10^{7}	3.56×10^{7}	9.59×10^{6}	4.57×10^{6}	7.12×10^{6}
DSTN (C/CS/EC)	1.24×10^{4}	1.50×10^{4}	8.16×10^{4}	1.57×10^{5}	2.21×10^{4}	1.21×10^{4}	6.13×10^{2}
DYNC1H1 (CS)	5.56×10^{3}	4.56×10^{3}	4.33×10^{4}	1.88×10^{5}	5.70×10^{3}	1.57×10^{3}	2.51×10^{3}
DYNLL1 (NU/CS/MT)	3.23×10^{2}	1.88×10^{3}	3.47×10^{4}	6.23×10^{4}	7.74×10^{2}	7.69×10^{2}	1.01×10^{3}
EEF1A1 (C/NU/PM)	3.13×10^{5}	4.12×10^{5}	4.01×10^{6}	7.69×10^{6}	4.14×10^{5}	1.56×10^{6}	2.64×10^{5}
EEF1B2 (C)	1.46×10^{4}	2.45×10^{4}	8.07×10^{4}	1.38×10^{5}	1.34×10^{4}	5.63×10^{4}	2.87×10^{4}
EEF1G (C/NU/EC)	2.83×10^{4}	1.61×10^{4}	5.32×10^{5}	7.27×10^{5}	2.22×10^{4}	7.26×10^{4}	8.15×10^{3}
EEF2 (C/NU)	1.61×10^{5}	1.25×10^{5}	1.97×10^{6}	1.57×10^{6}	1.46×10^{5}	3.93×10^{5}	1.34×10^{5}
EIF3L (C)	1.67×10^{3}	1.34×10^{3}	1.88×10^{3}	1.74×10^{4}	8.15×10^{2}	3.89×10^{3}	7.72×10^{2}
EIF4A1 (C/EC)	3.68×10^{4}	2.66×10^{4}	7.53×10^{5}	7.50×10^{5}	2.18×10^{4}	6.61×10^{4}	1.31×10^{4}
EIF5A (C/NU/ER)	2.31×10^{3}	3.77×10^{3}	6.57×10^{4}	1.37×10^{5}	1.24×10^{3}	1.42×10^{4}	3.05×10^{3}
EIF6 (C/NU)	3.34×10^{4}	7.12×10^{4}	2.62×10^{5}	1.57×10^{5}	6.49×10^{4}	2.11×10^{4}	2.60×10^{4}
ENO1 (C/NU)	8.95×10^{5}	1.53×10^{6}	9.07×10^{6}	3.74×10^{6}	8.45×10^{5}	1.41×10^{6}	9.57×10^{5}
EPPK1 (CS)	1.71×10^{5}	1.26×10^{5}	4.07×10^{6}	5.11×10^{6}	1.81×10^{5}	6.66×10^{4}	1.12×10^{5}
EPRS1 (C/PM)	3.94×10^{3}	1.24×10^{3}	8.37×10^{4}	3.86×10^{4}	1.86×10^{3}	8.58×10^{2}	1.07×10^{3}
ERH (NU)	2.17×10^{5}	6.43×10^{5}	4.24×10^{5}	4.10×10^{5}	2.68×10^{5}	3.28×10^{5}	4.04×10^{5}
ERP29 (ER)	7.84×10^{1}	8.17×10^{0}	0	1.74×10^{4}	0	2.17×10^{1}	0
ERP44 (ER)	2.89×10^{4}	3.89×10^{4}	3.69×10^{4}	2.25×10^{4}	5.42×10^{4}	7.44×10^{4}	4.66×10^{4}
ETF1 (C)	0	0	0	9.51×10^{3}	0	0	0
ETFA (MT)	0	9.27×10^{2}	0	1.54×10^{4}	0	2.64×10^{3}	0
EVPL (CS)	4.69×10^{4}	7.47×10^{4}	7.67×10^{5}	1.71×10^{6}	2.78×10^{4}	3.61×10^{4}	3.16×10^{4}
EWSR1 (C/NU/PM)	9.50×10^{3}	4.14×10^{4}	3.70×10^{4}	8.79×10^{4}	3.33×10^{4}	2.63×10^{4}	3.22×10^{4}
EZR (PM/CS)	6.54×10^{4}	3.83×10^{4}	2.57×10^{5}	5.89×10^{5}	2.10×10^{4}	7.57×10^{4}	2.83×10^{4}
FABP5 (C/NU/EC)	2.53×10^{5}	2.57×10^{5}	2.93×10^{6}	5.32×10^{5}	9.93×10^{4}	1.03×10^{5}	1.08×10^{5}
FASN (C)	1.61×10^{3}	2.35×10^{3}	1.69×10^{5}	1.26×10^{5}	4.20×10^{3}	1.41×10^{3}	1.91×10^{3}
FLG (C/PM)	7.98×10^{5}	1.29×10^{6}	1.33×10^{7}	2.32×10^{6}	7.69×10^{5}	4.13×10^{5}	8.14×10^{5}
FLG2 (C)	6.49×10^{6}	5.40×10^{6}	5.63×10^{7}	3.03×10^{6}	6.81×10^{6}	2.46×10^{6}	7.71×10^{6}
FLNA (CS)	8.74×10^{3}	9.83×10^{3}	2.44×10^{5}	3.74×10^{5}	9.12×10^{3}	2.69×10^{4}	1.99×10^{4}
FLNB (CS)	1.49×10^{5}	9.72×10^{4}	7.52×10^{5}	1.07×10^{6}	1.21×10^{5}	8.30×10^{4}	6.08×10^{4}
FUS (NU)	4.42×10^{4}	1.04×10^{5}	1.21×10^{5}	2.17×10^{5}	3.11×10^{5}	5.81×10^{4}	2.08×10^{5}
GANAB (GA/ER)	5.89×10^{3}	9.88×10^{3}	6.01×10^{3}	4.38×10^{4}	1.02×10^{4}	3.99×10^{4}	9.69×10^{3}
GAPDH (C/NU/CS)	1.94×10^{6}	1.64×10^{6}	9.92×10^{6}	4.30×10^{6}	2.12×10^{6}	1.73×10^{6}	2.32×10^{6}
GARS1 (C/EC)	1.04×10^{2}	1.30×10^{3}	9.72×10^{2}	2.37×10^{4}	4.45×10^{0}	2.04×10^{3}	8.37×10^{0}
GDI1 (C/GA)	8.17×10^{4}	9.61×10^{4}	5.10×10^{5}	2.98×10^{5}	6.04×10^{4}	7.78×10^{4}	1.14×10^{5}
GFUS (C/EC)	0	4.61×10^{2}	1.43×10^{4}	6.37×10^{4}	9.60×10^{1}	1.68×10^{3}	6.47×10^{2}
GGCT(C/EC)	4.35×10^{5}	3.20×10^{5}	2.19×10^{6}	3.32×10^{5}	5.29×10^{5}	1.40×10^{5}	3.46×10^{5}
GGH (LY /EC)	6.72×10^{4}	1.27×10^{5}	3.05×10^{5}	1.03×10^{5}	9.46×10^{4}	1.71×10^{4}	2.35×10^{4}
GLOD4 (MT)	0	2.16×10^{3}	5.34×10^{4}	3.64×10^{4}	3.88×10^{3}	1.38×10^{4}	1.53×10^{3}
GLUD1 (ER/MT)	0	3.01×10^{2}	1.83×10^{3}	2.43×10^{4}	7.61×10^{2}	8.21×10^{2}	7.73×10^{2}
GLUL(C/MT/PM/ER)	8.31×10^{3}	1.61×10^{4}	7.93×10^{5}	5.15×10^{4}	8.13×10^{3}	1.66×10^{4}	1.59×10^{4}
GM2A (LY)	1.61×10^{4}	1.36×10^{4}	3.24×10^{5}	6.19×10^{4}	1.16×10^{3}	1.89×10^{3}	4.22×10^{3}
GOT2 (MT/PM)	9.28×10^{3}	1.51×10^{4}	2.58×10^{4}	7.67×10^{4}	3.83×10^{3}	4.28×10^{4}	9.89×10^{2}
GPHN (C/PM)	7.43×10^{3}	3.78×10^{3}	0	4.33×10^{3}	5.49×10^{3}	2.60×10^{4}	3.19×10^{3}
GPI (C/ER)	1.42×10^{3}	6.15×10^{3}	5.86×10^{4}	8.74×10^{4}	3.84×10^{3}	3.92×10^{3}	4.49×10^{2}
GSDMA (C/PM)	2.60×10^{5}	1.88×10^{5}	2.21×10^{6}	4.22×10^{5}	1.95×10^{5}	6.86×10^{4}	1.66×10^{5}
GSN (CS/EC)	1.10×10^{5}	5.34×10^{4}	8.28×10^{5}	4.14×10^{6}	6.04×10^{4}	2.18×10^{5}	5.29×10^{4}
GSR (C/MT)	1.43×10^{3}	2.39×10^{4}	1.57×10^{4}	6.12×10^{4}	1.52×10^{5}	4.53×10^{5}	3.77×10^{4}
GSTP1 (C/NU/MT)	2.45×10^{4}	8.27×10^{4}	2.14×10^{6}	2.04×10^{6}	5.67×10^{4}	4.91×10^{4}	2.76×10^{4}

Table S3
(page 4)

raw abundance

- 1×10^{7}
- 8×10^{6}
- 6×10^{6}
- 4×10^{6}
- 2×10^{6}
- 0

po-H_2O_2 / c-YAP1C

0	2	5	15	30	60	- 3-AT 15	
1.21×10^{4}	1.10×10^{4}	5.46×10^{4}	1.80×10^{5}	1.08×10^{4}	3.89×10^{3}	9.07×10^{3}	H1-0 (NU)
1.76×10^{5}	5.00×10^{5}	1.97×10^{5}	1.87×10^{6}	3.32×10^{5}	2.25×10^{5}	5.12×10^{5}	H1-2 (NU)
9.26×10^{3}	1.11×10^{4}	6.64×10^{4}	3.88×10^{5}	1.03×10^{4}	9.51×10^{2}	6.82×10^{3}	H1-5 (NU)
7.37×10^{2}	4.65×10^{3}	2.55×10^{3}	1.36×10^{5}	0	6.30×10^{2}	3.49×10^{3}	H2AC4 (NU)
7.33×10^{3}	1.47×10^{4}	2.22×10^{4}	7.07×10^{5}	1.26×10^{4}	2.84×10^{3}	4.22×10^{3}	H2AC6 (NU)
1.05×10^{4}	2.84×10^{4}	4.36×10^{4}	3.96×10^{5}	5.62×10^{3}	3.40×10^{3}	9.15×10^{3}	H2BC11 (NU)
6.94×10^{4}	1.38×10^{4}	1.06×10^{5}	7.81×10^{5}	8.89×10^{3}	7.18×10^{3}	1.44×10^{4}	H3-3A (NU)
9.29×10^{5}	3.67×10^{5}	3.15×10^{6}	9.81×10^{6}	1.68×10^{5}	1.05×10^{5}	2.14×10^{5}	H4C1 (NU)
7.94×10^{3}	4.20×10^{3}	5.86×10^{4}	1.61×10^{5}	1.18×10^{4}	1.79×10^{3}	2.80×10^{3}	HADHA (MT)
1.26×10^{5}	1.31×10^{5}	1.16×10^{6}	2.84×10^{5}	1.32×10^{5}	4.83×10^{4}	6.00×10^{4}	HAL (C)
1.04×10^{4}	3.51×10^{3}	6.38×10^{4}	1.51×10^{4}	3.05×10^{3}	2.05×10^{3}	2.19×10^{3}	HARS1 (C)
6.18×10^{3}	5.56×10^{2}	4.56×10^{3}	2.38×10^{4}	7.38×10^{2}	1.09×10^{4}	7.05×10^{2}	HK1 (C/MT)
2.31×10^{2}	1.35×10^{2}	1.20×10^{3}	4.38×10^{4}	5.31×10^{2}	0	1.90×10^{2}	HLA-DRA (ER/LY/ES/PM))
3.39×10^{4}	7.11×10^{4}	1.01×10^{5}	3.53×10^{5}	7.25×10^{4}	1.54×10^{5}	1.29×10^{5}	HNRNPA1 (NU/C)
7.59×10^{3}	1.10×10^{4}	4.81×10^{4}	8.86×10^{4}	8.97×10^{3}	7.72×10^{3}	1.45×10^{4}	HNRNPA2B1 (C/NU/EC)
8.17×10^{3}	1.08×10^{4}	7.30×10^{4}	1.89×10^{5}	1.31×10^{4}	9.60×10^{3}	9.04×10^{3}	HNRNPA3 (NU)
1.24×10^{4}	4.70×10^{4}	3.80×10^{4}	1.90×10^{5}	9.34×10^{2}	1.71×10^{4}	9.46×10^{2}	HNRNPC (NU)
4.09×10^{3}	3.45×10^{3}	1.13×10^{4}	8.81×10^{4}	4.08×10^{2}	8.52×10^{3}	1.98×10^{2}	HNRNPD (C/NU)
5.78×10^{2}	0	5.87×10^{3}	2.53×10^{4}	5.74×10^{2}	4.30×10^{1}	0	HNRNPF (NU)
2.31×10^{2}	1.72×10^{3}	1.23×10^{5}	1.08×10^{5}	8.85×10^{2}	1.59×10^{3}	0	HNRNPH2 (NU)
2.80×10^{3}	1.01×10^{4}	1.13×10^{5}	5.02×10^{5}	1.71×10^{3}	1.80×10^{4}	3.08×10^{2}	HNRNPK (C/NU)
7.22×10^{3}	9.81×10^{3}	1.00×10^{5}	2.18×10^{5}	1.72×10^{3}	2.24×10^{3}	1.74×10^{3}	HNRNPM (NU)
1.06×10^{4}	4.26×10^{4}	3.83×10^{4}	1.61×10^{5}	5.67×10^{3}	1.55×10^{3}	2.23×10^{2}	HNRNPU (C/NU/CS)
8.61×10^{3}	2.35×10^{4}	1.09×10^{5}	1.74×10^{5}	1.27×10^{5}	7.44×10^{5}	4.00×10^{4}	HPRT1 (C)
0	0	0	1.53×10^{4}	0	2.00×10^{3}	4.08×10^{2}	HSD17B10 (MT)
1.57×10^{4}	1.01×10^{4}	4.18×10^{5}	1.82×10^{5}	6.86×10^{3}	1.22×10^{4}	1.25×10^{4}	HSD17B4 (PO)
7.14×10^{4}	1.06×10^{5}	9.27×10^{5}	1.20×10^{6}	1.35×10^{5}	5.30×10^{5}	8.44×10^{4}	HSP90AA1 (C/NU/MT/PM)
1.37×10^{4}	3.07×10^{4}	4.02×10^{5}	9.35×10^{5}	5.31×10^{4}	3.12×10^{5}	3.72×10^{4}	HSP90AB1 (C/NU/PM/EC)
4.98×10^{4}	9.53×10^{4}	2.77×10^{5}	6.18×10^{5}	1.99×10^{5}	2.92×10^{5}	2.09×10^{5}	HSP90B1 (ER)
6.35×10^{5}	9.54×10^{5}	2.16×10^{6}	3.32×10^{6}	7.88×10^{5}	1.13×10^{6}	8.62×10^{5}	HSPA1B (C/CS)
1.23×10^{6}	1.53×10^{6}	4.19×10^{6}	2.85×10^{6}	1.28×10^{6}	1.38×10^{6}	1.19×10^{6}	HSPA5 (C/ER)
7.39×10^{5}	1.48×10^{6}	2.43×10^{6}	3.87×10^{6}	5.00×10^{5}	4.43×10^{5}	4.17×10^{5}	HSPA8 (C/PM/NU)
1.69×10^{5}	4.26×10^{5}	1.53×10^{5}	3.05×10^{5}	1.13×10^{5}	2.23×10^{5}	1.37×10^{5}	HSPA9 (MT)
3.48×10^{5}	3.64×10^{5}	1.30×10^{7}	8.13×10^{6}	1.20×10^{6}	3.65×10^{5}	3.98×10^{5}	HSPB1 (C/NU/CS)
1.07×10^{5}	1.35×10^{5}	4.05×10^{6}	5.29×10^{5}	7.47×10^{4}	6.39×10^{4}	5.57×10^{4}	IDE (C/PM/EC)
7.46×10^{3}	4.08×10^{3}	1.09×10^{5}	8.29×10^{4}	5.61×10^{3}	2.75×10^{2}	2.28×10^{3}	IL1RN (C/EC)
2.02×10^{3}	1.82×10^{3}	7.59×10^{4}	1.65×10^{5}	2.89×10^{2}	3.90×10^{2}	8.46×10^{2}	IL36RN (C/EC)
1.20×10^{4}	1.78×10^{4}	1.60×10^{5}	4.11×10^{5}	3.54×10^{4}	1.36×10^{4}	1.46×10^{4}	IQGAP1 (C/NU/PM)
1.65×10^{4}	6.63×10^{3}	2.70×10^{4}	1.21×10^{4}	7.56×10^{4}	2.54×10^{5}	9.69×10^{3}	ISYNA1 (C)
1.03×10^{5}	7.96×10^{4}	3.06×10^{6}	5.44×10^{6}	1.23×10^{5}	5.16×10^{4}	9.18×10^{4}	IVL (C)
6.99×10^{6}	4.57×10^{6}	3.55×10^{7}	1.05×10^{7}	5.57×10^{6}	2.63×10^{6}	3.43×10^{6}	JUP (CS/PM)
4.87×10^{4}	3.27×10^{4}	3.54×10^{5}	1.82×10^{5}	3.72×10^{4}	2.25×10^{4}	3.22×10^{4}	LAMP1 (LY)
8.93×10^{2}	1.37×10^{3}	4.45×10^{5}	7.98×10^{3}	6.62×10^{3}	3.31×10^{2}	2.73×10^{3}	LCN2 (EC/VE)
3.70×10^{2}	3.55×10^{3}	2.57×10^{2}	5.03×10^{4}	2.53×10^{3}	2.21×10^{4}	2.79×10^{3}	LCP1 (PM/CS)
1.19×10^{5}	1.68×10^{5}	1.44×10^{6}	2.21×10^{6}	1.35×10^{5}	1.88×10^{5}	1.28×10^{5}	LDHA (C)
4.09×10^{4}	9.07×10^{4}	4.50×10^{4}	8.67×10^{4}	1.05×10^{5}	1.05×10^{5}	8.33×10^{4}	LDHB (C/MT)
5.09×10^{4}	9.35×10^{4}	5.17×10^{5}	1.37×10^{6}	3.49×10^{4}	7.65×10^{4}	3.98×10^{4}	LGALS3 (C/NU/EC)
2.55×10^{5}	4.60×10^{5}	2.38×10^{7}	2.51×10^{7}	3.72×10^{5}	2.91×10^{5}	3.51×10^{5}	LGALS7 (C/NU/EC)
1.72×10^{4}	1.03×10^{4}	1.96×10^{5}	3.97×10^{4}	4.13×10^{3}	2.38×10^{3}	3.89×10^{3}	LGALSL (?)
9.58×10^{4}	1.05×10^{5}	4.52×10^{6}	4.23×10^{6}	6.20×10^{4}	4.92×10^{4}	5.72×10^{4}	LMNA (NU)

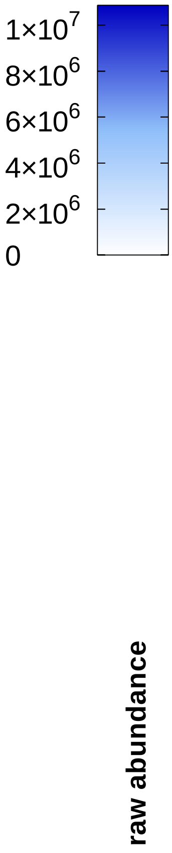

po-H_2O_2 / c-YAP1C

Protein	0	2	5	15	30	60	- 3-AT 15
LMNB1 (NU)	2.80×10^{3}	1.01×10^{4}	3.16×10^{4}	3.30×10^{4}	2.07×10^{2}	3.09×10^{3}	8.48×10^{2}
LMNB2 (NU)	5.58×10^{2}	3.32×10^{3}	1.76×10^{4}	1.66×10^{4}	0	0	0
LTA4H (C)	2.41×10^{2}	6.19×10^{2}	8.14×10^{4}	4.77×10^{4}	1.08×10^{3}	1.35×10^{4}	8.74×10^{0}
LY6D (PM)	5.01×10^{3}	3.88×10^{3}	1.64×10^{5}	1.73×10^{5}	3.30×10^{3}	3.77×10^{3}	2.90×10^{3}
MACROH2A1 (NU)	0	1.51×10^{3}	1.54×10^{3}	1.59×10^{5}	1.16×10^{1}	9.21×10^{1}	7.07×10^{3}
MAP4 (CS)	1.52×10^{3}	4.57×10^{3}	3.45×10^{3}	3.92×10^{4}	2.83×10^{3}	6.59×10^{3}	1.06×10^{4}
MDH1 (C)	2.17×10^{4}	2.31×10^{4}	2.82×10^{5}	2.11×10^{5}	8.61×10^{3}	1.95×10^{4}	6.54×10^{3}
MDH2 (MT)	3.81×10^{4}	7.68×10^{4}	3.06×10^{5}	4.62×10^{5}	8.40×10^{4}	6.18×10^{4}	5.37×10^{4}
MSN (CS/PM)	3.28×10^{3}	7.53×10^{3}	7.12×10^{4}	1.37×10^{5}	5.77×10^{3}	2.02×10^{4}	3.17×10^{3}
MTAP (C/NU)	1.02×10^{3}	2.76×10^{3}	1.96×10^{4}	3.74×10^{3}	4.60×10^{3}	2.69×10^{3}	1.85×10^{3}
MTHFD1 (C)	0	1.21×10^{0}	0	1.14×10^{4}	1.58×10^{3}	1.51×10^{4}	0
MTPN (C/NU)	2.61×10^{3}	6.61×10^{3}	2.42×10^{2}	6.13×10^{4}	3.22×10^{2}	1.67×10^{4}	7.46×10^{2}
MYH14 (C/CS/EC)	1.34×10^{3}	2.24×10^{3}	2.63×10^{5}	2.29×10^{5}	2.79×10^{3}	5.67×10^{3}	1.62×10^{4}
MYH9 (CS)	9.98×10^{4}	9.28×10^{4}	2.17×10^{6}	2.53×10^{6}	1.28×10^{5}	1.06×10^{5}	7.13×10^{4}
MYL12A (C/CS/EC)	1.28×10^{3}	4.88×10^{2}	3.60×10^{4}	1.39×10^{5}	6.43×10^{1}	6.28×10^{3}	9.26×10^{2}
MYL6 (C/CS/EC)	1.95×10^{4}	3.23×10^{4}	2.25×10^{5}	6.62×10^{5}	1.14×10^{4}	3.07×10^{4}	1.61×10^{4}
NAGK (C/EC)	2.60×10^{4}	1.64×10^{4}	2.73×10^{5}	1.78×10^{5}	2.98×10^{4}	3.12×10^{4}	7.55×10^{3}
NAMPT (C/NU/EC)	1.63×10^{3}	5.78×10^{2}	2.29×10^{5}	1.14×10^{5}	1.30×10^{3}	7.68×10^{2}	6.09×10^{2}
NAPA (PM)	7.12×10^{0}	1.34×10^{2}	3.63×10^{4}	3.08×10^{4}	1.16×10^{2}	6.00×10^{2}	1.29×10^{2}
NCCRP1 (C/NU/MT)	1.12×10^{5}	7.11×10^{4}	1.87×10^{6}	4.10×10^{5}	4.57×10^{5}	1.52×10^{5}	9.73×10^{4}
NCL (C/NU)	1.11×10^{3}	4.55×10^{3}	1.60×10^{2}	7.49×10^{4}	0	3.28×10^{3}	1.69×10^{3}
NDRG1 (C/NU/PM/CS)	0	0	1.59×10^{3}	2.98×10^{4}	0	0	0
NDRG2 (C)	3.96×10^{2}	0	9.41×10^{3}	2.49×10^{4}	0	3.79×10^{2}	0
NEU2 (C)	1.99×10^{4}	1.22×10^{4}	2.02×10^{5}	1.43×10^{5}	1.39×10^{4}	4.14×10^{3}	8.39×10^{3}
NONO (NU)	4.18×10^{3}	2.93×10^{2}	5.47×10^{3}	2.57×10^{4}	8.44×10^{2}	1.20×10^{4}	5.06×10^{2}
NPEPPS (C/NU)	3.56×10^{4}	3.53×10^{4}	4.23×10^{5}	1.51×10^{5}	7.66×10^{4}	1.38×10^{5}	2.57×10^{4}
NT5C3A (C/ER)	1.95×10^{0}	0	7.67×10^{4}	9.17×10^{3}	0	2.19×10^{2}	0
NUDC (CS/NU)	4.61×10^{3}	8.62×10^{3}	3.58×10^{3}	1.76×10^{4}	1.45×10^{4}	1.49×10^{5}	3.77×10^{3}
OTUB1 (C)	1.72×10^{3}	7.88×10^{2}	7.43×10^{3}	4.63×10^{4}	0	1.29×10^{2}	4.52×10^{2}
P4HB (PM/ER)	8.42×10^{4}	1.46×10^{5}	6.32×10^{5}	9.03×10^{5}	2.01×10^{5}	1.78×10^{5}	1.29×10^{5}
PABPC1 (C/NU)	1.97×10^{4}	1.68×10^{4}	2.63×10^{5}	2.02×10^{5}	2.14×10^{4}	1.80×10^{4}	7.87×10^{3}
PAFAH1B1 (NU/CS/PM)	3.96×10^{3}	5.30×10^{2}	6.25×10^{4}	2.78×10^{4}	1.71×10^{3}	4.04×10^{2}	9.27×10^{2}
PAICS (C/EC)	1.28×10^{2}	3.70×10^{3}	3.37×10^{4}	1.79×10^{4}	1.32×10^{4}	1.87×10^{4}	1.09×10^{3}
PC (MT)	4.34×10^{3}	1.05×10^{3}	1.09×10^{4}	3.65×10^{4}	1.25×10^{3}	8.39×10^{4}	1.01×10^{3}
PCCA (MT)	3.36×10^{5}	6.75×10^{5}	1.27×10^{5}	2.87×10^{5}	4.10×10^{5}	1.27×10^{6}	3.54×10^{5}
PCYOX1 (LY)	0	2.06×10^{2}	2.21×10^{4}	2.76×10^{3}	1.03×10^{3}	0	0
PDCD6IP (C/CS)	6.02×10^{2}	5.82×10^{2}	6.60×10^{4}	1.28×10^{5}	9.70×10^{2}	4.20×10^{2}	8.08×10^{2}
PDIA3 (ER)	4.71×10^{4}	8.27×10^{4}	3.79×10^{5}	9.71×10^{5}	9.38×10^{4}	1.43×10^{5}	9.14×10^{4}
PDIA4 (ER)	1.85×10^{3}	7.93×10^{3}	4.77×10^{4}	5.94×10^{4}	3.44×10^{3}	1.51×10^{4}	2.06×10^{3}
PEBP1 (C)	1.23×10^{5}	7.05×10^{4}	5.97×10^{5}	2.87×10^{5}	6.50×10^{4}	3.65×10^{4}	4.47×10^{4}
PFN1 (CS)	6.77×10^{4}	6.90×10^{4}	3.53×10^{5}	1.00×10^{6}	3.64×10^{4}	2.20×10^{5}	9.45×10^{4}
PGK1 (C)	7.19×10^{4}	5.85×10^{4}	1.30×10^{6}	1.03×10^{6}	3.05×10^{4}	1.64×10^{5}	1.74×10^{4}
PHB2 (C/NU/MT/PM)	4.36×10^{3}	6.64×10^{3}	3.02×10^{4}	1.31×10^{5}	2.58×10^{3}	2.93×10^{3}	4.54×10^{3}
PHGDH (C/EC)	3.21×10^{4}	1.19×10^{4}	1.60×10^{5}	6.10×10^{5}	9.84×10^{3}	1.62×10^{4}	1.28×10^{4}
PKM (C/NU)	1.41×10^{5}	1.99×10^{5}	3.54×10^{6}	2.94×10^{6}	2.70×10^{5}	2.75×10^{5}	1.20×10^{5}
PKP1 (NU)	9.63×10^{5}	3.12×10^{5}	3.99×10^{6}	3.91×10^{6}	1.01×10^{6}	2.44×10^{5}	3.63×10^{5}
PKP3 (NU)	2.82×10^{3}	3.64×10^{3}	7.10×10^{4}	5.40×10^{5}	6.74×10^{3}	9.28×10^{2}	1.98×10^{3}
PLA2G4E (C/PM/LY/ES)	9.89×10^{2}	4.65×10^{2}	2.89×10^{4}	7.12×10^{3}	8.26×10^{2}	2.34×10^{3}	9.16×10^{2}
PLEC (CS)	8.45×10^{4}	6.18×10^{4}	6.54×10^{5}	1.42×10^{6}	4.07×10^{4}	3.94×10^{4}	5.16×10^{4}
PLP2 (ER/PM)	5.24×10^{3}	9.22×10^{3}	4.32×10^{4}	2.52×10^{5}	1.60×10^{4}	8.07×10^{2}	4.45×10^{3}

Table S3
(page 6)

raw abundance (colour scale): 1×10^{7}, 8×10^{6}, 6×10^{6}, 4×10^{6}, 2×10^{6}, 0

po-H_2O_2 / c-YAP1C						- 3-AT	
0	2	5	15	30	60	15	
1.01×10^{4}	1.45×10^{4}	5.10×10^{5}	5.24×10^{5}	4.03×10^{4}	2.31×10^{5}	1.24×10^{4}	PLS3 (C)
4.50×10^{4}	2.44×10^{4}	3.17×10^{5}	1.07×10^{5}	7.71×10^{4}	3.32×10^{4}	4.01×10^{4}	PNP (C)
3.33×10^{5}	2.80×10^{5}	6.14×10^{6}	1.59×10^{6}	2.58×10^{5}	1.21×10^{5}	2.01×10^{5}	POF1B (C/CS)
6.42×10^{3}	8.59×10^{3}	1.07×10^{5}	7.89×10^{4}	1.79×10^{4}	2.25×10^{4}	9.34×10^{3}	PPA1 (C)
1.73×10^{5}	3.91×10^{5}	7.33×10^{5}	2.65×10^{6}	4.42×10^{5}	8.90×10^{5}	3.51×10^{5}	PPIA (C/NU/EC)
5.94×10^{3}	7.18×10^{3}	1.29×10^{5}	1.80×10^{5}	3.98×10^{3}	4.11×10^{3}	5.66×10^{3}	PPIB (ER)
6.17×10^{4}	3.45×10^{4}	1.40×10^{6}	1.63×10^{6}	3.31×10^{4}	1.28×10^{4}	1.71×10^{4}	PPL (C/CS/PM)
7.17×10^{5}	3.24×10^{6}	5.69×10^{6}	8.14×10^{6}	1.34×10^{7}	2.86×10^{7}	4.29×10^{6}	PRDX1 (C)
3.35×10^{5}	1.20×10^{6}	2.67×10^{6}	2.21×10^{6}	3.62×10^{6}	5.10×10^{6}	1.28×10^{6}	PRDX2 (C)
2.72×10^{5}	4.77×10^{5}	3.09×10^{5}	7.22×10^{5}	9.18×10^{5}	2.27×10^{6}	8.21×10^{5}	PRDX3 (MT/C/EE)
1.38×10^{4}	6.61×10^{4}	5.82×10^{4}	5.65×10^{4}	8.56×10^{4}	1.30×10^{5}	5.79×10^{4}	PRDX4 (C/ER)
4.81×10^{4}	4.47×10^{4}	4.24×10^{5}	6.98×10^{5}	6.86×10^{5}	2.12×10^{6}	9.53×10^{4}	PRDX6 (C/LY)
2.53×10^{2}	2.96×10^{2}	4.99×10^{4}	1.13×10^{4}	0	9.96×10^{2}	0	PREP (C)
2.44×10^{3}	5.08×10^{3}	1.14×10^{4}	3.37×10^{4}	3.14×10^{5}	1.17×10^{4}	1.12×10^{3}	PRKD2 (C/NU/GA/PM)
1.95×10^{3}	3.52×10^{3}	8.92×10^{3}	1.95×10^{4}	1.89×10^{4}	1.14×10^{5}	7.27×10^{3}	PRMT5 (C/NU/GA)
4.44×10^{2}	9.81×10^{2}	1.34×10^{4}	1.45×10^{4}	1.93×10^{3}	6.17×10^{2}	1.12×10^{2}	PRXL2A (C/EC)
4.28×10^{3}	1.11×10^{4}	1.27×10^{5}	2.29×10^{4}	2.80×10^{4}	8.95×10^{3}	1.05×10^{4}	PSMA2 (C/NU)
2.05×10^{4}	1.77×10^{4}	2.12×10^{5}	7.28×10^{5}	3.14×10^{4}	9.69×10^{3}	1.47×10^{4}	PSMA3 (C/NU)
5.04×10^{4}	5.96×10^{4}	5.38×10^{5}	2.02×10^{5}	2.48×10^{5}	2.61×10^{4}	5.82×10^{4}	PSMA5 (C/NU)
3.72×10^{4}	2.52×10^{4}	2.48×10^{5}	8.77×10^{4}	3.76×10^{4}	9.28×10^{3}	2.32×10^{4}	PSMA6 (C/NU)
7.14×10^{4}	3.79×10^{4}	4.55×10^{5}	1.06×10^{5}	8.19×10^{4}	2.29×10^{4}	4.86×10^{4}	PSMA7 (C/NU)
1.99×10^{4}	3.24×10^{4}	5.77×10^{4}	1.59×10^{4}	4.72×10^{4}	9.62×10^{3}	6.75×10^{3}	PSMB2 (C/NU)
1.92×10^{4}	1.63×10^{4}	3.25×10^{5}	8.74×10^{4}	3.90×10^{4}	1.04×10^{4}	1.49×10^{4}	PSMB4 (C/NU)
7.24×10^{3}	3.95×10^{3}	4.97×10^{4}	9.50×10^{3}	6.75×10^{3}	2.07×10^{3}	3.71×10^{3}	PSMB5 (C/NU)
2.05×10^{4}	2.37×10^{4}	1.71×10^{5}	9.27×10^{4}	2.76×10^{4}	1.32×10^{4}	1.96×10^{4}	PSMB6 (C/NU)
3.57×10^{3}	8.09×10^{2}	3.15×10^{4}	1.20×10^{4}	2.21×10^{3}	1.78×10^{3}	1.18×10^{3}	PSMB8 (C/NU)
0	3.28×10^{2}	5.11×10^{3}	1.67×10^{4}	0	1.69×10^{2}	0	PSMC3 (C/NU)
0	1.36×10^{3}	5.67×10^{3}	2.39×10^{4}	0	2.38×10^{3}	9.34×10^{-1}	PSMC5 (C/NU)
0	1.22×10^{2}	2.30×10^{4}	3.60×10^{4}	0	1.40×10^{4}	0	PSMD14 (C/NU/EC)
4.71×10^{3}	1.34×10^{4}	1.11×10^{5}	1.01×10^{5}	1.89×10^{4}	1.48×10^{4}	4.72×10^{4}	PTBP1 (NU)
2.65×10^{3}	6.13×10^{3}	7.46×10^{2}	1.99×10^{4}	3.63×10^{3}	3.35×10^{2}	3.95×10^{3}	PTCD1 (MT)
5.85×10^{3}	4.09×10^{3}	2.80×10^{3}	3.86×10^{4}	2.98×10^{3}	2.93×10^{3}	2.10×10^{3}	PTMA (NU)
2.59×10^{3}	3.91×10^{2}	5.22×10^{2}	3.02×10^{4}	2.90×10^{3}	3.32×10^{2}	0	PYCARD (C/NU/MT/ER)
1.07×10^{4}	1.13×10^{4}	8.74×10^{4}	9.00×10^{4}	1.09×10^{4}	5.63×10^{3}	6.34×10^{3}	RAB10 (N/CS/ER/GA/ES
2.99×10^{3}	4.83×10^{3}	7.69×10^{4}	1.82×10^{5}	5.52×10^{3}	7.94×10^{2}	1.83×10^{3}	RAB11A (PM/ES)
1.38×10^{4}	1.19×10^{4}	1.34×10^{5}	1.74×10^{5}	4.70×10^{3}	4.97×10^{3}	4.67×10^{3}	RAB14 (GA/ES)
4.60×10^{3}	2.98×10^{4}	8.36×10^{4}	9.78×10^{4}	1.75×10^{4}	1.12×10^{4}	4.92×10^{3}	RAB1A (C/ES/GA/PM)
5.29×10^{3}	3.33×10^{3}	6.56×10^{4}	5.03×10^{4}	1.14×10^{3}	0	8.90×10^{2}	RAB2A (GA/ER)
4.27×10^{4}	2.30×10^{4}	2.77×10^{5}	2.10×10^{5}	2.40×10^{4}	1.85×10^{4}	1.42×10^{4}	RAB7A (LY/ES)
5.72×10^{4}	1.06×10^{5}	3.62×10^{5}	4.06×10^{5}	6.09×10^{4}	1.03×10^{5}	8.19×10^{4}	RACK1 (C/NU/PM)
1.72×10^{2}	8.98×10^{1}	1.69×10^{4}	1.11×10^{4}	9.44×10^{1}	4.10×10^{2}	3.21×10^{2}	RAD23B (C/NU)
1.15×10^{5}	2.11×10^{5}	3.55×10^{5}	3.59×10^{5}	1.32×10^{5}	2.20×10^{5}	1.87×10^{5}	RAN (C/NU)
3.71×10^{2}	6.52×10^{3}	2.44×10^{4}	3.78×10^{4}	2.34×10^{2}	6.20×10^{2}	1.65×10^{-1}	RARS1 (C)
5.00×10^{3}	1.72×10^{3}	2.14×10^{5}	2.94×10^{5}	3.27×10^{3}	3.58×10^{3}	1.65×10^{3}	RNH1 (C)
1.77×10^{3}	3.42×10^{3}	7.26×10^{3}	7.22×10^{4}	5.45×10^{3}	9.61×10^{3}	3.60×10^{3}	RPL10 (C/NU/ER)
3.27×10^{4}	8.36×10^{4}	1.04×10^{5}	3.33×10^{5}	1.90×10^{4}	6.08×10^{4}	2.36×10^{4}	RPL11 (C/NU)
6.79×10^{3}	9.70×10^{3}	4.78×10^{4}	1.79×10^{5}	4.42×10^{3}	5.27×10^{3}	4.72×10^{3}	RPL12 (C/EC)
2.44×10^{3}	1.88×10^{4}	6.54×10^{3}	1.36×10^{5}	7.47×10^{3}	1.57×10^{4}	1.33×10^{4}	RPL13 (C)
4.26×10^{3}	9.15×10^{3}	2.20×10^{4}	1.62×10^{5}	8.92×10^{3}	1.77×10^{4}	1.06×10^{4}	RPL13A (C)
3.53×10^{3}	9.33×10^{3}	1.02×10^{4}	1.91×10^{5}	9.00×10^{3}	1.46×10^{4}	1.61×10^{4}	RPL14 (C/EC)

Table S3
(page 7)

raw abundance (colour scale): 1×10^{7}, 8×10^{6}, 6×10^{6}, 4×10^{6}, 2×10^{6}, 0

po-H_2O_2 / c-YAP1C

						- 3-AT	
**0**	**2**	**5**	**15**	**30**	**60**	**15**	
8.50×10^{3}	3.29×10^{4}	3.89×10^{4}	2.13×10^{5}	4.14×10^{4}	2.28×10^{4}	5.90×10^{4}	RPL15 (PM)
1.84×10^{3}	1.30×10^{4}	2.92×10^{3}	9.17×10^{4}	8.22×10^{3}	1.54×10^{4}	6.13×10^{3}	RPL17-C18orf32 (C)
1.09×10^{4}	2.37×10^{4}	7.64×10^{4}	4.85×10^{5}	6.12×10^{4}	5.47×10^{4}	3.95×10^{4}	RPL18A (C/PM)
3.08×10^{4}	3.18×10^{4}	2.71×10^{4}	1.11×10^{5}	2.82×10^{4}	2.70×10^{4}	3.77×10^{4}	RPL19 (CC/NU)
1.27×10^{3}	3.35×10^{3}	1.04×10^{2}	8.29×10^{4}	5.22×10^{3}	7.40×10^{3}	3.76×10^{3}	RPL21 (C/ER)
1.51×10^{4}	6.67×10^{4}	2.22×10^{5}	2.72×10^{5}	7.93×10^{3}	4.63×10^{4}	2.14×10^{4}	RPL22 (C/NU/EC)
1.44×10^{4}	2.78×10^{4}	4.21×10^{4}	1.54×10^{5}	1.00×10^{4}	1.30×10^{4}	1.84×10^{4}	RPL23 (C/NU/EC)
1.43×10^{5}	1.65×10^{5}	1.62×10^{5}	4.25×10^{5}	1.74×10^{5}	1.51×10^{5}	2.74×10^{5}	RPL23A (C/NU/EC)
4.85×10^{3}	1.55×10^{4}	3.16×10^{3}	1.73×10^{5}	1.06×10^{4}	1.48×10^{4}	1.42×10^{4}	RPL27A (C/ER)
1.82×10^{3}	7.95×10^{3}	2.23×10^{3}	1.04×10^{5}	1.77×10^{3}	9.55×10^{3}	4.49×10^{3}	RPL28 5C/EC)
0	2.60×10^{4}	7.00×10^{3}	1.68×10^{5}	4.40×10^{4}	2.72×10^{4}	7.53×10^{4}	RPL29 (C)
3.83×10^{4}	1.64×10^{4}	2.24×10^{4}	1.77×10^{5}	9.20×10^{3}	1.55×10^{4}	9.27×10^{3}	RPL3 (C/NU)
1.20×10^{2}	5.97×10^{2}	0	5.77×10^{3}	3.94×10^{1}	7.01×10^{2}	2.05×10^{2}	RPL36A (C)
1.94×10^{4}	2.43×10^{4}	3.66×10^{4}	2.72×10^{5}	2.44×10^{4}	3.49×10^{4}	3.28×10^{4}	RPL4 (C/NU/ER/EC)
8.38×10^{3}	9.87×10^{3}	5.51×10^{3}	1.40×10^{5}	5.25×10^{3}	1.67×10^{4}	9.10×10^{3}	RPL5 (C/NU)
1.65×10^{4}	5.15×10^{4}	1.76×10^{5}	6.23×10^{5}	7.06×10^{4}	7.05×10^{4}	1.02×10^{5}	RPL6 (C/ER)
2.13×10^{4}	3.70×10^{4}	4.50×10^{4}	3.76×10^{5}	3.00×10^{4}	6.30×10^{4}	3.96×10^{4}	RPL7 (C/NU)
1.57×10^{4}	8.18×10^{4}	1.09×10^{5}	6.07×10^{5}	1.19×10^{5}	7.41×10^{4}	1.88×10^{5}	RPL7A (C/NU)
2.23×10^{3}	1.18×10^{4}	1.74×10^{4}	1.28×10^{5}	8.66×10^{3}	1.51×10^{4}	1.23×10^{4}	RPL8 (C)
6.88×10^{3}	2.07×10^{4}	6.95×10^{4}	5.32×10^{5}	4.62×10^{4}	3.52×10^{4}	1.30×10^{4}	RPLP0 (C/NU)
6.42×10^{4}	7.65×10^{4}	6.68×10^{5}	1.60×10^{6}	2.85×10^{4}	6.77×10^{4}	4.74×10^{4}	RPLP2 (C/EC)
2.02×10^{4}	2.71×10^{4}	1.56×10^{5}	8.79×10^{4}	3.40×10^{4}	4.31×10^{4}	3.08×10^{4}	RPN1 (ER)
3.68×10^{3}	1.48×10^{4}	4.49×10^{4}	2.56×10^{4}	5.52×10^{3}	4.63×10^{3}	3.07×10^{3}	RPN2 (ER)
2.38×10^{4}	1.55×10^{5}	3.41×10^{4}	1.82×10^{5}	2.49×10^{4}	4.96×10^{4}	3.84×10^{4}	RPS10 (C/NU)
1.26×10^{4}	2.63×10^{4}	4.48×10^{4}	2.21×10^{5}	1.47×10^{4}	2.88×10^{4}	2.75×10^{4}	RPS13 (C/NU/EC)
1.37×10^{4}	2.22×10^{4}	6.38×10^{3}	1.22×10^{5}	1.16×10^{4}	2.04×10^{4}	1.49×10^{4}	RPS14 (C/NU/EC)
4.94×10^{4}	4.60×10^{4}	1.48×10^{5}	2.21×10^{5}	3.91×10^{4}	2.93×10^{4}	3.17×10^{4}	RPS15A (C/NU/EC)
3.81×10^{4}	8.11×10^{4}	3.16×10^{4}	1.96×10^{5}	2.04×10^{4}	4.39×10^{4}	3.26×10^{4}	RPS18 (C)
3.70×10^{4}	1.50×10^{5}	1.55×10^{4}	2.47×10^{5}	4.39×10^{4}	7.34×10^{4}	7.20×10^{4}	RPS19 (NU)
5.27×10^{3}	5.59×10^{3}	5.53×10^{4}	1.01×10^{5}	1.57×10^{4}	1.10×10^{4}	1.00×10^{4}	RPS21 (C/ER)
2.97×10^{3}	8.07×10^{3}	1.79×10^{3}	6.39×10^{4}	6.21×10^{2}	6.34×10^{3}	5.43×10^{3}	RPS23 (C/ER)
4.26×10^{4}	1.29×10^{5}	1.17×10^{5}	3.83×10^{5}	6.40×10^{4}	9.44×10^{4}	6.68×10^{4}	RPS25 (C/NU/EC)
1.84×10^{6}	1.82×10^{6}	1.04×10^{7}	5.58×10^{6}	2.37×10^{6}	1.43×10^{6}	1.58×10^{6}	RPS27A (C/NU)
4.20×10^{3}	3.77×10^{3}	5.11×10^{3}	5.01×10^{4}	1.72×10^{3}	3.48×10^{3}	2.17×10^{3}	RPS28 (C/ER)
1.05×10^{5}	1.87×10^{5}	5.64×10^{5}	9.09×10^{5}	9.54×10^{4}	1.74×10^{5}	7.54×10^{4}	RPS3 (C/NU/MT/CS)
1.25×10^{3}	3.95×10^{3}	1.32×10^{4}	1.16×10^{5}	1.08×10^{3}	5.78×10^{3}	2.01×10^{3}	RPS3A (C/NU)
4.49×10^{3}	1.83×10^{4}	9.06×10^{3}	2.18×10^{5}	8.98×10^{3}	2.09×10^{4}	7.23×10^{3}	RPS4X (C)
1.16×10^{4}	4.84×10^{4}	2.38×10^{4}	5.99×10^{4}	8.53×10^{3}	1.18×10^{4}	1.52×10^{4}	RPS5 (C/NU/EC)
2.07×10^{3}	8.57×10^{3}	1.89×10^{3}	3.60×10^{4}	4.27×10^{3}	8.59×10^{3}	6.83×10^{3}	RPS6 (C/NU/ER)
4.81×10^{3}	2.11×10^{4}	3.43×10^{4}	1.18×10^{5}	6.40×10^{3}	2.17×10^{4}	2.17×10^{4}	RPS7 (C)
3.41×10^{4}	1.30×10^{5}	3.83×10^{5}	6.90×10^{5}	7.32×10^{4}	1.26×10^{5}	2.77×10^{5}	RPS8 (C/PM)
6.20×10^{3}	1.93×10^{4}	4.37×10^{5}	6.73×10^{5}	4.07×10^{4}	9.49×10^{4}	6.31×10^{4}	RPSA (NU/C/PM)
6.53×10^{3}	5.62×10^{4}	4.99×10^{4}	2.02×10^{4}	7.23×10^{2}	2.48×10^{3}	1.81×10^{2}	RTCB (C/NU)
4.43×10^{2}	2.26×10^{2}	3.03×10^{4}	9.60×10^{4}	5.38×10^{2}	2.73×10^{1}	7.61×10^{2}	RTN4 (PM/ER)
1.60×10^{5}	8.34×10^{4}	8.82×10^{5}	4.13×10^{5}	2.50×10^{5}	6.63×10^{4}	1.05×10^{5}	S100A14 (C)
2.74×10^{4}	1.82×10^{4}	2.55×10^{5}	1.09×10^{5}	2.96×10^{4}	5.41×10^{3}	1.84×10^{4}	S100A16 (C/NU)
1.08×10^{5}	9.53×10^{4}	3.62×10^{6}	1.66×10^{5}	1.23×10^{5}	8.65×10^{4}	1.00×10^{5}	S100A7 (C/EC)
0	3.03×10^{1}	4.06×10^{5}	1.51×10^{4}	9.79×10^{1}	5.82×10^{1}	2.65×10^{1}	S100A7A (C)
3.72×10^{4}	7.12×10^{4}	2.75×10^{7}	9.38×10^{5}	1.68×10^{5}	5.39×10^{4}	3.96×10^{5}	S100A8 (C/PM/EC/CS)
4.43×10^{5}	5.93×10^{5}	8.20×10^{7}	3.38×10^{6}	8.22×10^{5}	5.12×10^{5}	1.26×10^{6}	S100A9 (C/CS/PM/EC)

Table S3 (page 8)

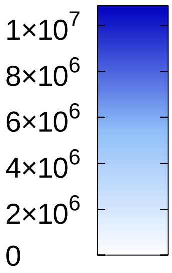

raw abundance

po-H_2O_2 / c-YAP1C

0	2	5	15	30	60	- 3-AT 15	
6.69×10^{4}	6.67×10^{4}	1.16×10^{6}	9.71×10^{5}	2.92×10^{4}	2.28×10^{4}	3.55×10^{4}	S100B (C/NU)
1.97×10^{3}	1.39×10^{4}	3.75×10^{5}	4.47×10^{4}	1.24×10^{4}	6.34×10^{2}	1.53×10^{4}	S100P (C/NU/PM)
2.85×10^{2}	8.29×10^{2}	5.37×10^{1}	4.40×10^{4}	5.38×10^{2}	1.10×10^{3}	6.60×10^{2}	SCEL (C/PM)
5.10×10^{2}	0	4.27×10^{4}	3.78×10^{4}	0	9.51×10^{2}	2.42×10^{2}	SDCBP2 (C/NU/PM)
2.16×10^{2}	2.48×10^{2}	2.29×10^{3}	1.50×10^{4}	4.33×10^{1}	8.79×10^{2}	2.75×10^{1}	SEPTIN7 (C/CS)
6.13×10^{3}	3.41×10^{4}	6.42×10^{3}	6.69×10^{4}	1.34×10^{4}	2.70×10^{4}	1.61×10^{4}	SERBP1 (C/NU)
1.53×10^{4}	8.88×10^{3}	1.59×10^{5}	4.71×10^{5}	6.30×10^{4}	1.10×10^{4}	6.14×10^{4}	SERPINA1 (ER/EC)
0	0	0	1.42×10^{4}	1.40×10^{1}	2.33×10^{3}	0	SERPINB1 (C/ES/LY/EC)
1.65×10^{6}	7.44×10^{5}	6.50×10^{6}	5.22×10^{5}	1.78×10^{6}	3.52×10^{5}	9.41×10^{5}	SERPINB12 (C)
2.11×10^{4}	6.61×10^{3}	1.36×10^{5}	6.69×10^{4}	7.10×10^{3}	3.38×10^{3}	4.64×10^{3}	SERPINB13 (C)
8.95×10^{4}	4.01×10^{4}	2.87×10^{5}	3.21×10^{5}	7.75×10^{4}	1.37×10^{4}	2.59×10^{4}	SERPINB2 (C/EC)
8.80×10^{2}	3.13×10^{3}	3.46×10^{4}	3.54×10^{3}	1.82×10^{3}	3.07×10^{2}	2.41×10^{3}	SERPINB7 (C)
3.42×10^{4}	8.79×10^{3}	1.15×10^{5}	4.72×10^{4}	1.33×10^{4}	2.72×10^{3}	4.27×10^{3}	SERPINB8 (C)
1.21×10^{5}	8.04×10^{4}	5.86×10^{6}	9.99×10^{6}	9.15×10^{4}	5.44×10^{4}	6.89×10^{4}	SFN (C/NU/EC)
5.85×10^{3}	1.11×10^{4}	5.41×10^{4}	1.63×10^{5}	1.39×10^{4}	3.17×10^{4}	7.78×10^{3}	SFPQ (C/NU)
7.06×10^{3}	2.43×10^{4}	0	6.39×10^{3}	2.54×10^{3}	2.63×10^{4}	3.26×10^{3}	SHMT2(C/NU/MT)
2.95×10^{3}	7.29×10^{3}	3.75×10^{4}	3.65×10^{4}	9.82×10^{4}	1.12×10^{6}	1.37×10^{4}	SKP1 (C/NU)
4.94×10^{4}	9.14×10^{4}	4.14×10^{4}	2.77×10^{5}	1.39×10^{4}	5.80×10^{4}	3.07×10^{4}	SLC25A5 (MT)
4.80×10^{3}	7.67×10^{3}	5.97×10^{3}	4.33×10^{4}	2.37×10^{3}	6.75×10^{3}	3.66×10^{3}	SLC25A6 (MT)
0	4.14×10^{2}	1.60×10^{4}	5.09×10^{4}	0	4.44×10^{3}	0	SND1 (C/NU)
9.49×10^{1}	1.53×10^{3}	0	5.21×10^{3}	1.48×10^{2}	5.75×10^{2}	1.54×10^{2}	SNRPB2 (NU)
1.13×10^{4}	2.59×10^{4}	6.03×10^{4}	5.14×10^{4}	1.21×10^{4}	1.19×10^{4}	1.54×10^{4}	SNRPD3 (C/NU)
1.60×10^{3}	9.59×10^{3}	1.42×10^{4}	2.16×10^{4}	3.56×10^{3}	9.24×10^{3}	4.38×10^{3}	SNRPF (C/NU)
1.30×10^{3}	2.52×10^{3}	5.07×10^{4}	6.39×10^{4}	1.19×10^{3}	5.20×10^{2}	0	SOD2 (MT)
3.94×10^{1}	1.44×10^{2}	3.28×10^{4}	1.05×10^{5}	3.29×10^{4}	9.90×10^{1}	6.38×10^{1}	SPTAN1 (CS/PM)
2.22×10^{4}	6.38×10^{4}	2.74×10^{4}	1.55×10^{5}	4.77×10^{4}	3.72×10^{4}	3.46×10^{4}	SRSF2 (NU)
1.65×10^{4}	6.52×10^{4}	9.64×10^{4}	8.57×10^{4}	7.30×10^{4}	5.53×10^{4}	3.56×10^{4}	SRSF7 (C/NU)
0	2.70×10^{3}	3.73×10^{3}	2.95×10^{4}	1.30×10^{3}	4.93×10^{3}	1.73×10^{3}	SSB (NU)
4.59×10^{3}	6.08×10^{3}	8.55×10^{4}	2.94×10^{4}	1.28×10^{4}	2.00×10^{3}	2.23×10^{3}	SSR4 (ER)
1.16×10^{3}	1.51×10^{3}	1.51×10^{3}	8.01×10^{4}	1.14×10^{3}	8.71×10^{3}	5.55×10^{2}	ST13 (C)
2.63×10^{4}	7.85×10^{3}	1.29×10^{5}	3.10×10^{5}	4.03×10^{3}	6.30×10^{3}	3.63×10^{3}	SULT2B1 (C/NU/ER)
1.48×10^{3}	7.12×10^{3}	8.18×10^{3}	6.28×10^{4}	2.84×10^{3}	1.60×10^{4}	2.43×10^{3}	SYNCRIP (C/NU/ER)
3.27×10^{4}	1.85×10^{4}	1.60×10^{5}	6.89×10^{4}	2.73×10^{4}	1.05×10^{4}	2.65×10^{4}	SYPL1 (VE)
0	0	3.47×10^{3}	6.59×10^{4}	0	4.02×10^{2}	0	TACSTD2 (PM)
4.77×10^{3}	2.70×10^{4}	3.93×10^{4}	1.18×10^{5}	2.77×10^{5}	3.37×10^{3}	2.11×10^{5}	TAF15 (C/NU)
1.96×10^{4}	6.78×10^{3}	4.51×10^{4}	4.97×10^{5}	2.54×10^{3}	6.73×10^{3}	3.99×10^{3}	TAGLN2 (C/EC)
1.42×10^{4}	1.84×10^{4}	2.73×10^{5}	1.75×10^{5}	2.87×10^{4}	2.87×10^{4}	2.82×10^{4}	TALDO1 (C)
3.42×10^{2}	6.82×10^{2}	5.48×10^{3}	5.20×10^{4}	5.51×10^{2}	1.70×10^{3}	0	TCP1 (C/CS)
4.21×10^{5}	3.51×10^{5}	3.38×10^{6}	7.06×10^{5}	6.75×10^{5}	3.59×10^{5}	4.14×10^{5}	TGM1 (C/PM)
8.67×10^{5}	9.07×10^{5}	6.40×10^{6}	1.25×10^{6}	1.10×10^{6}	3.50×10^{5}	5.55×10^{5}	TGM3 (C)
6.80×10^{3}	4.69×10^{3}	1.65×10^{5}	1.74×10^{4}	1.19×10^{4}	7.22×10^{2}	9.51×10^{2}	TGM5 (C)
1.24×10^{5}	6.94×10^{4}	7.45×10^{5}	3.22×10^{5}	4.60×10^{4}	3.09×10^{4}	3.97×10^{4}	TKT (C/NU/ER/PO/EC)
1.10×10^{4}	1.24×10^{4}	9.88×10^{4}	9.20×10^{4}	1.35×10^{4}	8.88×10^{3}	1.15×10^{4}	TMED10 (PM/ER/GA)
0	0	4.83×10^{4}	0	0	0	7.76×10^{2}	TMEM40 (PM)
1.38×10^{4}	1.89×10^{4}	8.98×10^{4}	6.12×10^{4}	1.49×10^{4}	6.88×10^{3}	8.58×10^{3}	TOLLIP (C)
1.75×10^{5}	1.58×10^{5}	2.01×10^{6}	7.92×10^{5}	9.31×10^{4}	7.89×10^{4}	6.96×10^{4}	TPI1 (C)
7.62×10^{4}	8.88×10^{4}	6.28×10^{5}	5.82×10^{5}	2.66×10^{5}	7.66×10^{5}	1.14×10^{5}	TPM3 (CS)
3.39×10^{3}	4.26×10^{3}	1.26×10^{4}	4.22×10^{4}	1.42×10^{4}	5.14×10^{4}	6.64×10^{3}	TPM4 (CS)
2.98×10^{3}	0	9.83×10^{3}	1.30×10^{5}	0	7.30×10^{2}	0	TPPP3 (C/CS)
4.00×10^{3}	2.76×10^{3}	1.60×10^{5}	1.99×10^{5}	2.20×10^{3}	5.45×10^{3}	1.21×10^{2}	TPT1 (C)

Table S3
(page 9)

raw abundance: 1×10^{7}, 8×10^{6}, 6×10^{6}, 4×10^{6}, 2×10^{6}, 0

po-H_2O_2 / c-YAP1C

	0	2	5	15	30	60	- 3-AT 15
TRIM29 (C/LY)	2.14×10^{4}	8.26×10^{4}	3.59×10^{6}	6.37×10^{5}	3.87×10^{5}	8.03×10^{4}	6.24×10^{4}
TUBA1C (CS)	3.05×10^{4}	5.67×10^{4}	4.33×10^{5}	1.14×10^{6}	5.27×10^{4}	1.06×10^{5}	4.04×10^{4}
TUBA4A (CS)	4.97×10^{3}	2.14×10^{3}	2.50×10^{5}	5.09×10^{5}	3.82×10^{3}	2.29×10^{3}	1.83×10^{3}
TUBB (CS)	2.05×10^{4}	1.72×10^{4}	1.27×10^{5}	4.48×10^{5}	1.70×10^{4}	4.62×10^{4}	1.51×10^{4}
TUBB4B (CS)	4.82×10^{3}	4.71×10^{3}	3.70×10^{5}	1.67×10^{6}	8.33×10^{3}	1.96×10^{4}	7.65×10^{3}
TUFM (MT)	2.67×10^{3}	1.07×10^{5}	2.37×10^{4}	8.94×10^{4}	1.43×10^{4}	3.96×10^{4}	1.11×10^{3}
TXN (C/NU/EC)	9.81×10^{5}	8.24×10^{5}	2.77×10^{6}	1.48×10^{6}	1.21×10^{6}	2.87×10^{6}	1.40×10^{6}
TYMP (C)	9.36×10^{3}	1.30×10^{4}	1.36×10^{6}	2.71×10^{5}	1.10×10^{4}	2.35×10^{4}	7.78×10^{3}
UBA1 (C/NU/MT)	1.04×10^{4}	1.25×10^{4}	1.51×10^{5}	3.96×10^{5}	3.24×10^{4}	1.15×10^{5}	2.16×10^{4}
UBE2N (C/NU)	1.03×10^{3}	2.36×10^{3}	4.18×10^{4}	9.10×10^{4}	2.43×10^{2}	4.97×10^{3}	6.77×10^{2}
UCHL1 (C/ER)	0	0	0	1.21×10^{3}	0	1.59×10^{4}	0
UCHL3 (C)	8.51×10^{1}	9.46×10^{2}	7.17×10^{3}	3.19×10^{4}	4.41×10^{0}	3.03×10^{4}	0
UGP2 (C)	1.48×10^{3}	4.15×10^{2}	2.69×10^{3}	9.83×10^{3}	0	9.22×10^{2}	4.22×10^{2}
USP5 (C/NU/LY)	2.16×10^{3}	1.31×10^{3}	4.36×10^{4}	4.77×10^{4}	1.55×10^{3}	8.46×10^{3}	1.81×10^{3}
VAT1 (C/MT)	1.50×10^{3}	6.19×10^{2}	2.25×10^{4}	1.32×10^{5}	2.65×10^{3}	1.29×10^{2}	3.53×10^{1}
VCL (PM/CS)	5.54×10^{4}	3.28×10^{4}	4.34×10^{5}	1.62×10^{5}	5.19×10^{4}	2.22×10^{4}	1.28×10^{4}
VCP (C/NU/ER)	3.54×10^{4}	3.39×10^{4}	1.05×10^{6}	7.19×10^{5}	3.96×10^{4}	5.23×10^{4}	8.39×10^{4}
VDAC1 (MT/PM)	3.69×10^{4}	1.98×10^{4}	5.67×10^{5}	3.90×10^{5}	2.17×10^{4}	1.08×10^{4}	1.76×10^{4}
VDAC2 (MT)	1.73×10^{4}	7.98×10^{3}	8.22×10^{4}	8.27×10^{4}	3.98×10^{3}	5.64×10^{3}	9.43×10^{3}
VIM (C/NU/PM)	5.20×10^{5}	1.42×10^{6}	2.47×10^{5}	1.97×10^{6}	5.68×10^{5}	3.79×10^{5}	6.37×10^{5}
WDR1 (C/CS)	4.94×10^{2}	4.27×10^{2}	2.06×10^{4}	4.97×10^{4}	9.09×10^{3}	2.36×10^{3}	2.45×10^{3}
WDR77 (C/NU)	1.17×10^{4}	8.76×10^{3}	2.44×10^{4}	1.76×10^{4}	2.59×10^{4}	1.16×10^{5}	3.53×10^{3}
XRCC6 (NU)	1.97×10^{3}	1.43×10^{3}	2.87×10^{4}	6.38×10^{4}	2.99×10^{3}	6.70×10^{3}	1.78×10^{3}
YWHAB (C)	2.03×10^{4}	1.34×10^{4}	1.83×10^{5}	4.37×10^{5}	1.73×10^{4}	4.52×10^{4}	1.62×10^{4}
YWHAE (C/NU)	1.29×10^{5}	1.21×10^{5}	3.65×10^{5}	5.46×10^{5}	1.06×10^{5}	2.07×10^{5}	1.71×10^{5}
YWHAG (C)	0	2.12×10^{2}	1.24×10^{4}	9.07×10^{3}	1.68×10^{2}	1.57×10^{3}	3.70×10^{2}
YWHAQ (C)	1.54×10^{3}	6.86×10^{3}	7.51×10^{4}	1.64×10^{5}	1.36×10^{4}	8.01×10^{4}	9.41×10^{3}
YWHAZ (C)	7.31×10^{4}	9.86×10^{4}	2.24×10^{6}	3.50×10^{6}	1.13×10^{5}	2.52×10^{5}	8.10×10^{4}
ZNF185 (CS)	9.94×10^{2}	2.55×10^{2}	3.71×10^{4}	8.87×10^{4}	1.03×10^{3}	4.80×10^{2}	5.44×10^{2}

TABLE S4. Heat map summarizing the raw abundances of proteins trapped by po-IBD-SBP-YAP1C in response to treatment of po-DD-DAO-expressing Flp-In T-REx 293 cells with D-Ala. DTT eluates of the experiment shown in Fig. S4C were processed for LC-MS/MS analysis. After validation, proteins enriched 2.5-fold or more in at least one of the time points (0, 2, 5, 15, 30, and 60 min) after H_2O_2 exposure were retrieved, named according to their UniProtKB gene name, and sorted in alphabetical order. The primary subcellular localizations of the proteins (according to the same database) are indicated between brackets. The values represent raw abundances and are color-coded (with white and blue being low and high, respectively). C, cytosol; CS, cytoskeleton; EC, extracellular; EE, early endosome; GA, Golgi apparatus; LY, lysosome; MT, mitochondria; NU, nucleus; PM, plasma membrane; PO, peroxisome.

Table S4 (page 1)

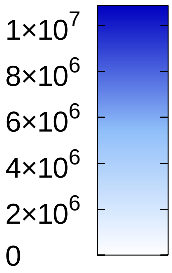

po-H_2O_2 / po-YAP1C

	0	2	5	15	30	60
ALOX12B (C)	2.97×10^{4}	6.40×10^{4}	6.59×10^{4}	1.84×10^{4}	1.72×10^{5}	4.60×10^{4}
ALOXE3 (C)	3.01×10^{3}	1.74×10^{4}	9.83×10^{3}	1.60×10^{3}	6.16×10^{4}	6.34×10^{3}
ANXA1 (C/NU/PM/EC)	5.26×10^{3}	6.43×10^{4}	4.57×10^{3}	1.37×10^{4}	1.37×10^{4}	4.89×10^{3}
ANXA2 (PM/EC)	5.23×10^{5}	8.14×10^{5}	8.38×10^{5}	5.45×10^{5}	2.48×10^{6}	1.96×10^{6}
ARF5 (NU/GA)	8.36×10^{3}	9.99×10^{3}	1.87×10^{4}	1.97×10^{4}	1.03×10^{4}	2.56×10^{4}
ARG1 (C)	3.74×10^{5}	4.96×10^{5}	5.02×10^{5}	1.97×10^{5}	2.78×10^{6}	3.86×10^{5}
ASAH1 (C/LY/EC)	3.36×10^{3}	3.02×10^{4}	2.05×10^{4}	5.39×10^{3}	3.37×10^{4}	7.84×10^{3}
ASPRV1 (C/NU)	7.54×10^{3}	1.75×10^{4}	4.60×10^{4}	1.75×10^{4}	8.24×10^{4}	4.60×10^{4}
BLMH (C)	1.52×10^{5}	7.29×10^{5}	3.96×10^{5}	2.05×10^{5}	2.31×10^{6}	2.36×10^{5}
CAPN1 (C/PM)	1.27×10^{4}	4.37×10^{4}	2.61×10^{4}	1.08×10^{4}	8.57×10^{4}	2.96×10^{4}
CASP14 (C/NU)	3.63×10^{5}	6.16×10^{5}	5.24×10^{5}	1.98×10^{5}	1.75×10^{6}	2.89×10^{5}
CAT (PO)	4.28×10^{5}	4.73×10^{5}	7.15×10^{5}	2.72×10^{5}	1.92×10^{6}	7.67×10^{5}
CDK4 (C/NU)	2.02×10^{3}	3.82×10^{3}	2.67×10^{3}	8.33×10^{2}	5.03×10^{4}	3.14×10^{4}
CDKN2A (C/NU)	2.38×10^{3}	2.97×10^{3}	2.49×10^{3}	1.87×10^{4}	3.72×10^{4}	1.62×10^{5}
CLU (C)	8.53×10^{4}	8.44×10^{4}	5.23×10^{6}	1.51×10^{5}	7.54×10^{5}	4.56×10^{4}
CRKL (C/NU/PM)	3.18×10^{1}	0	7.92×10^{3}	2.51×10^{2}	2.76×10^{3}	1.62×10^{4}
CRNN (C)	0	5.54×10^{4}	0	0	0	0
CSTB (C/NU)	5.21×10^{3}	3.95×10^{4}	2.01×10^{4}	7.00×10^{4}	9.24×10^{4}	5.23×10^{5}
CTSA (LY)	7.28×10^{3}	1.40×10^{4}	1.92×10^{4}	7.16×10^{3}	8.23×10^{4}	1.56×10^{4}
CTSD (LY/EC)	1.38×10^{5}	1.65×10^{5}	3.72×10^{5}	1.44×10^{5}	7.12×10^{5}	1.72×10^{5}
DSC1 (PM)	1.47×10^{6}	1.48×10^{6}	3.34×10^{6}	1.16×10^{6}	7.63×10^{6}	2.51×10^{6}
DSC3 (PM)	1.54×10^{5}	1.95×10^{5}	3.62×10^{5}	1.03×10^{5}	9.35×10^{5}	2.63×10^{5}
DSG1 (PM)	4.14×10^{6}	4.86×10^{6}	7.85×10^{6}	3.06×10^{6}	1.85×10^{7}	6.81×10^{6}
EEF1A1 (C/NU/PM)	6.33×10^{4}	1.28×10^{5}	1.26×10^{5}	9.69×10^{4}	6.24×10^{4}	2.86×10^{5}
FABP5 (C/NU/EC)	2.00×10^{5}	2.76×10^{5}	7.78×10^{5}	4.19×10^{5}	4.32×10^{5}	2.45×10^{6}
FLG (C/PM)	3.04×10^{5}	2.41×10^{5}	8.91×10^{5}	2.09×10^{5}	1.63×10^{6}	4.75×10^{5}
FLG2 (C)	1.54×10^{6}	2.42×10^{6}	2.89×10^{6}	1.14×10^{6}	8.77×10^{6}	2.13×10^{6}
GDA (C)	2.07×10^{3}	1.00×10^{3}	1.97×10^{3}	1.39×10^{3}	1.27×10^{4}	2.46×10^{3}
GGCT(C/EC)	1.26×10^{5}	1.56×10^{5}	2.41×10^{5}	7.24×10^{4}	5.43×10^{5}	1.46×10^{5}
GSDMA (C/PM)	9.22×10^{4}	2.48×10^{5}	2.76×10^{5}	1.22×10^{5}	8.37×10^{5}	1.72×10^{5}
GSR (C/MT)	1.18×10^{3}	2.13×10^{3}	2.36×10^{3}	2.59×10^{4}	3.72×10^{4}	1.88×10^{5}
GSTP1 (C/NU/MT)	5.09×10^{3}	1.91×10^{4}	3.60×10^{4}	3.36×10^{4}	2.34×10^{4}	5.85×10^{4}
HAL (C)	5.72×10^{4}	2.16×10^{5}	9.71×10^{4}	7.65×10^{4}	7.70×10^{5}	1.05×10^{5}
HPRT1 (C)	2.38×10^{3}	1.31×10^{3}	6.44×10^{3}	2.26×10^{4}	5.75×10^{4}	2.03×10^{5}
HSD17B4 (PO)	1.96×10^{2}	6.13×10^{3}	4.00×10^{3}	8.80×10^{2}	3.38×10^{3}	1.06×10^{4}
HSP90AA1 (C/NU/MT/PM)	1.23×10^{4}	4.52×10^{3}	7.91×10^{3}	1.55×10^{4}	1.56×10^{4}	3.17×10^{4}
HSPA9 (MT)	4.22×10^{3}	6.16×10^{2}	6.60×10^{3}	1.69×10^{3}	3.12×10^{3}	3.38×10^{4}
HSPB1 (C/NU/CS)	1.34×10^{5}	2.59×10^{5}	1.22×10^{5}	1.58×10^{5}	1.37×10^{5}	8.24×10^{5}
HSPD1 (MT)	2.53×10^{4}	1.29×10^{4}	3.67×10^{4}	2.39×10^{4}	1.33×10^{4}	8.05×10^{4}
LAMP1 (LY)	1.64×10^{4}	2.75×10^{4}	5.84×10^{4}	2.03×10^{4}	1.01×10^{5}	4.42×10^{4}
LGALS7 (C/NU/EC)	1.20×10^{5}	1.90×10^{5}	1.71×10^{5}	2.16×10^{5}	1.36×10^{5}	1.17×10^{6}
NCCRP1 (C/NU/MT)	7.92×10^{4}	1.75×10^{5}	1.94×10^{5}	8.07×10^{4}	4.11×10^{5}	2.14×10^{5}
NPM1 (NU)	2.30×10^{4}	2.95×10^{4}	3.92×10^{4}	4.41×10^{4}	9.01×10^{4}	2.70×10^{5}
PCCB (MT)	1.09×10^{5}	3.57×10^{4}	2.62×10^{5}	5.69×10^{4}	3.96×10^{4}	1.17×10^{6}
PCMT1 (C)	1.81×10^{3}	1.24×10^{3}	2.69×10^{3}	5.67×10^{2}	1.37×10^{4}	2.09×10^{4}
PEBP1 (C)	1.29×10^{4}	5.27×10^{3}	1.95×10^{4}	1.26×10^{4}	4.73×10^{4}	3.79×10^{4}
PKM (C/NU)	9.59×10^{3}	1.17×10^{4}	2.70×10^{4}	1.58×10^{4}	4.61×10^{4}	6.51×10^{4}
PLEC (CS)	3.80×10^{3}	1.45×10^{4}	9.41×10^{3}	2.39×10^{3}	2.72×10^{4}	6.19×10^{3}
PLS3 (C)	3.33×10^{3}	5.51×10^{3}	8.99×10^{2}	7.61×10^{3}	1.42×10^{4}	6.66×10^{4}
PNP (C)	4.33×10^{3}	9.15×10^{3}	9.29×10^{3}	1.89×10^{3}	2.21×10^{4}	7.78×10^{3}

Table S4
(page 2)

po-H_2O_2 / po-YAP1C

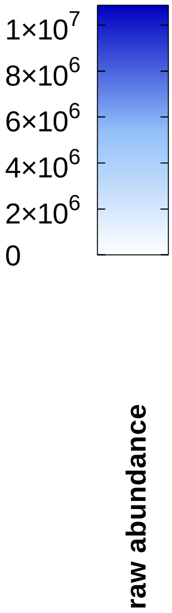

	0	2	5	15	30	60
POF1B (C/CS)	6.65×10^{4}	8.84×10^{4}	1.50×10^{5}	4.06×10^{4}	1.14×10^{5}	1.75×10^{5}
PPP2R2A (C/NU)	7.63×10^{3}	5.71×10^{3}	1.78×10^{4}	1.97×10^{4}	6.71×10^{4}	2.33×10^{5}
PRDX1 (C)	3.34×10^{5}	3.75×10^{5}	1.02×10^{6}	2.03×10^{6}	3.95×10^{6}	1.04×10^{7}
PRDX2 (C)	3.03×10^{5}	1.92×10^{5}	4.30×10^{5}	5.43×10^{5}	1.35×10^{6}	1.62×10^{6}
PRDX3 (MT/C/EE)	5.21×10^{3}	4.61×10^{3}	6.55×10^{3}	2.05×10^{4}	2.52×10^{4}	7.11×10^{4}
PRDX6 (C/LY)	3.27×10^{4}	2.71×10^{4}	1.23×10^{4}	3.05×10^{4}	1.22×10^{5}	5.38×10^{5}
PRMT5 (C/NU/GA)	2.04×10^{3}	2.84×10^{3}	4.74×10^{3}	2.19×10^{3}	8.83×10^{3}	4.73×10^{4}
PSMA7 (C/NU)	8.78×10^{3}	1.16×10^{4}	1.25×10^{4}	4.63×10^{3}	3.08×10^{4}	1.17×10^{4}
PSMB5 (C/NU)	2.18×10^{3}	3.71×10^{3}	6.93×10^{3}	1.87×10^{3}	1.55×10^{4}	6.53×10^{3}
RPS27A (C/NU)	1.61×10^{5}	1.73×10^{5}	2.84×10^{5}	1.35×10^{5}	4.33×10^{5}	3.21×10^{5}
S100A14 (C)	5.04×10^{4}	7.87×10^{4}	1.90×10^{5}	4.05×10^{4}	2.09×10^{5}	1.21×10^{5}
S100A16 (C/NU)	1.17×10^{4}	4.17×10^{4}	4.13×10^{4}	1.46×10^{4}	1.00×10^{5}	2.57×10^{4}
SERPINB7 (C)	5.78×10^{3}	1.15×10^{4}	2.63×10^{4}	7.39×10^{3}	3.04×10^{4}	1.56×10^{4}
SFN (C/NU/EC)	2.91×10^{3}	2.81×10^{3}	4.12×10^{2}	2.26×10^{4}	1.76×10^{3}	1.72×10^{4}
SKP1 (C/NU)	8.16×10^{4}	1.65×10^{4}	2.15×10^{4}	1.49×10^{5}	1.16×10^{6}	5.56×10^{6}
SYPL1 (VE)	1.13×10^{4}	5.75×10^{4}	3.03×10^{4}	7.06×10^{3}	5.11×10^{4}	4.51×10^{4}
TGM1 (C/PM)	1.80×10^{5}	3.45×10^{5}	3.27×10^{5}	1.18×10^{5}	7.39×10^{5}	2.48×10^{5}
TPI1 (C)	4.02×10^{4}	4.01×10^{4}	9.24×10^{4}	3.99×10^{4}	7.99×10^{4}	1.05×10^{5}
TRAP1 (MT)	2.35×10^{4}	1.36×10^{4}	1.54×10^{4}	4.04×10^{4}	1.64×10^{4}	6.98×10^{4}
TTR (C/EC)	7.50×10^{2}	1.54×10^{3}	1.02×10^{3}	3.15×10^{3}	9.30×10^{4}	8.75×10^{2}
TXN (C/NU/EC)	3.24×10^{5}	4.42×10^{5}	5.54×10^{5}	4.59×10^{5}	1.35×10^{6}	1.92×10^{6}
YWHAB (C)	1.68×10^{3}	1.18×10^{3}	1.59×10^{2}	8.15×10^{3}	4.98×10^{3}	4.16×10^{4}
YWHAG (C)	2.82×10^{2}	1.99×10^{0}	2.05×10^{2}	1.23×10^{3}	0	7.10×10^{3}
YWHAQ (C)	3.16×10^{3}	9.14×10^{2}	5.14×10^{2}	9.87×10^{3}	1.12×10^{4}	6.63×10^{4}
YWHAZ (C)	8.69×10^{3}	6.15×10^{3}	6.54×10^{3}	3.85×10^{4}	1.78×10^{4}	1.52×10^{5}

TABLE S5. Heat map summarizing the raw abundances of proteins trapped by mt-IBD-SBP-YAP1C in response to treatment of po-DD-DAO-expressing Flp-In T-REx 293 cells with D-Ala. DTT eluates of the experiment shown in Fig. S4B were processed for LC-MS/MS analysis. After validation, proteins enriched 2.5-fold or more in at least one of the time points (0, 2, 5, 15, 30, and 60 min) after H_2O_2 exposure were retrieved, named according to their UniProtKB gene name, and sorted in alphabetical order. The primary subcellular localizations of the proteins (according to the same database) are indicated between brackets. The values represent raw abundances and are color-coded (with white and blue being low and high, respectively). C, cytosol; CS, cytoskeleton; EC, extracellular; EE, early endosome; ER, endoplasmic reticulum; GA, Golgi apparatus; LY, lysosome; MT, mitochondria; NU, nucleus; PM, plasma membrane.

Table S5

po-H_2O_2 / mt-YAP1C

Protein	0	2	5	15	30	60
ACTG1 (CS)	1.52×10^{6}	2.31×10^{6}	3.31×10^{6}	2.39×10^{6}	4.07×10^{6}	3.30×10^{6}
ANXA1 (C/NU/PM/EC)	5.82×10^{3}	1.63×10^{4}	1.35×10^{4}	9.57×10^{4}	2.19×10^{5}	8.09×10^{3}
ANXA2 (PM/EC)	4.44×10^{5}	7.15×10^{5}	1.29×10^{6}	8.44×10^{5}	1.40×10^{6}	1.30×10^{6}
ARHGDIA (C)	8.24×10^{3}	1.08×10^{4}	2.74×10^{4}	3.53×10^{3}	2.42×10^{4}	2.60×10^{5}
ATP5F1A (MT)	3.69×10^{3}	3.63×10^{4}	2.68×10^{4}	1.80×10^{4}	8.58×10^{4}	3.29×10^{4}
ATP5F1B (MT)	8.57×10^{4}	2.00×10^{5}	1.86×10^{5}	1.45×10^{5}	2.66×10^{5}	2.82×10^{5}
BLMH (C)	1.20×10^{5}	6.50×10^{4}	1.81×10^{5}	1.92×10^{5}	2.45×10^{5}	3.79×10^{5}
CDK4 (C/NU)	1.86×10^{2}	5.82×10^{3}	3.02×10^{3}	5.70×10^{3}	8.55×10^{4}	1.41×10^{5}
CKB (C)	6.99×10^{4}	1.07×10^{5}	9.52×10^{4}	1.09×10^{5}	2.61×10^{5}	1.77×10^{5}
CSTA (C)	2.24×10^{5}	4.64×10^{5}	4.20×10^{5}	6.56×10^{5}	4.21×10^{5}	2.05×10^{5}
CSTB (C/NU)	8.50×10^{2}	1.87×10^{4}	1.72×10^{4}	1.07×10^{5}	2.66×10^{5}	7.06×10^{4}
CTSA (LY)	4.49×10^{3}	6.95×10^{3}	1.53×10^{4}	5.52×10^{3}	6.28×10^{3}	3.56×10^{3}
CTSD (LY/EC)	4.36×10^{3}	4.86×10^{4}	1.04×10^{5}	3.54×10^{4}	6.01×10^{4}	5.79×10^{4}
DSC3 (PM)	1.47×10^{4}	5.69×10^{4}	1.97×10^{5}	2.07×10^{4}	7.29×10^{4}	1.04×10^{5}
DSG1 (PM)	2.87×10^{6}	5.54×10^{6}	8.09×10^{6}	4.86×10^{6}	5.84×10^{6}	7.18×10^{6}
EEF1A1 (C/NU/PM)	1.08×10^{5}	2.12×10^{5}	4.12×10^{5}	1.59×10^{5}	2.03×10^{5}	7.41×10^{5}
ERP44 (ER)	3.45×10^{4}	3.69×10^{4}	3.29×10^{4}	3.77×10^{4}	5.77×10^{4}	9.88×10^{4}
FABP5 (C/NU/EC)	4.44×10^{4}	1.80×10^{5}	3.87×10^{5}	3.78×10^{5}	1.75×10^{5}	1.19×10^{5}
GGCT(C/EC)	7.44×10^{4}	1.12×10^{5}	1.79×10^{5}	1.54×10^{5}	1.74×10^{5}	1.95×10^{5}
GSR (C/MT)	2.63×10^{4}	6.27×10^{4}	9.57×10^{4}	2.52×10^{5}	7.38×10^{5}	1.05×10^{6}
HNRNPA1 (NU/C)	3.97×10^{2}	6.94×10^{3}	6.21×10^{3}	2.50×10^{3}	7.01×10^{3}	6.48×10^{3}
HPRT1 (C)	6.23×10^{2}	1.52×10^{4}	2.53×10^{4}	5.23×10^{4}	1.50×10^{5}	2.27×10^{5}
HSD17B10 (MT)	2.04×10^{4}	5.49×10^{4}	1.05×10^{5}	2.70×10^{5}	7.21×10^{5}	1.72×10^{6}
HSP90AA1 (C/NU/MT/PM)	1.79×10^{4}	1.63×10^{4}	1.16×10^{4}	9.09×10^{3}	4.23×10^{4}	1.33×10^{5}
HSP90AB1 (C/NU/PM/EC)	3.79×10^{3}	5.24×10^{3}	9.77×10^{3}	3.84×10^{3}	3.13×10^{4}	8.71×10^{4}
HSPB1 (C/NU/CS)	1.89×10^{4}	1.19×10^{5}	5.26×10^{5}	1.92×10^{5}	2.11×10^{5}	1.62×10^{5}
HSPD1 (MT)	2.21×10^{5}	2.04×10^{5}	2.58×10^{5}	2.22×10^{5}	3.86×10^{5}	6.23×10^{5}
JUP (CS/PM)	1.58×10^{6}	4.01×10^{6}	4.04×10^{6}	3.45×10^{6}	2.94×10^{6}	2.91×10^{6}
LGALS7 (C/NU/EC)	1.08×10^{5}	2.50×10^{5}	3.19×10^{6}	2.90×10^{5}	2.82×10^{5}	2.01×10^{5}
PC (MT)	0	0	1.73×10^{2}	0	5.86×10^{2}	2.19×10^{5}
PCCB (MT)	1.58×10^{5}	3.49×10^{5}	3.13×10^{5}	3.82×10^{5}	7.76×10^{5}	1.96×10^{6}
PCMT1 (C)	0	2.08×10^{4}	3.40×10^{4}	1.87×10^{4}	4.49×10^{4}	6.65×10^{4}
PKP1 (NU)	2.00×10^{5}	4.28×10^{5}	6.72×10^{5}	4.27×10^{5}	2.52×10^{5}	1.79×10^{5}
POF1B (C/CS)	8.02×10^{4}	1.13×10^{5}	3.14×10^{5}	1.28×10^{5}	1.19×10^{5}	8.58×10^{4}
PPIA (C/NU/EC)	2.15×10^{4}	1.40×10^{5}	1.51×10^{5}	7.30×10^{4}	2.89×10^{5}	4.99×10^{5}
PPP2R2A (C/NU)	4.38×10^{2}	1.03×10^{4}	1.97×10^{4}	4.93×10^{4}	1.04×10^{5}	1.57×10^{5}
PRDX1 (C)	7.59×10^{5}	1.56×10^{6}	4.36×10^{6}	6.22×10^{6}	1.26×10^{7}	1.83×10^{7}
PRDX2 (C)	2.49×10^{5}	4.18×10^{5}	9.18×10^{5}	1.05×10^{6}	1.61×10^{6}	1.64×10^{6}
PRDX3 (MT/C/EE)	3.47×10^{5}	2.64×10^{6}	9.53×10^{6}	8.45×10^{6}	1.43×10^{7}	2.15×10^{7}
PRDX6 (C/LY)	3.18×10^{3}	1.28×10^{4}	3.65×10^{4}	8.26×10^{4}	4.37×10^{5}	5.70×10^{5}
PRMT5 (C/NU/GA)	3.43×10^{3}	3.72×10^{3}	9.76×10^{3}	1.80×10^{4}	4.61×10^{4}	8.25×10^{4}
RPSA (NU/C/PM)	1.09×10^{4}	4.56×10^{4}	4.10×10^{4}	2.33×10^{4}	5.12×10^{4}	3.27×10^{4}
S100A14 (C)	5.42×10^{4}	5.95×10^{4}	1.43×10^{5}	9.71×10^{4}	7.93×10^{4}	6.20×10^{4}
S100A7 (C/EC)	4.19×10^{3}	1.74×10^{5}	2.67×10^{5}	7.93×10^{4}	1.63×10^{5}	1.45×10^{5}
SERPINB3 (C)	9.84×10^{4}	2.21×10^{5}	4.57×10^{5}	2.88×10^{5}	4.57×10^{5}	4.37×10^{5}
SKP1 (C/NU)	7.69×10^{2}	2.26×10^{3}	1.40×10^{4}	5.14×10^{4}	4.20×10^{5}	1.09×10^{6}
TGM3 (C)	7.18×10^{4}	2.49×10^{5}	4.26×10^{5}	1.91×10^{5}	3.90×10^{5}	3.28×10^{5}
TUBA1C (CS)	1.02×10^{3}	1.09×10^{5}	4.26×10^{4}	9.09×10^{3}	1.68×10^{5}	8.09×10^{4}
TXN (C/NU/EC)	3.19×10^{5}	5.27×10^{5}	7.39×10^{5}	8.25×10^{5}	1.96×10^{6}	2.43×10^{6}
UCHL1 (C/ER)	1.47×10^{4}	7.21×10^{3}	9.42×10^{3}	7.20×10^{3}	1.99×10^{4}	3.95×10^{4}
VDAC1 (MT/PM)	0	2.64×10^{3}	1.36×10^{4}	0	1.32×10^{4}	0
YWHAQ (C)	8.01×10^{3}	6.92×10^{3}	7.82×10^{3}	1.59×10^{4}	4.80×10^{4}	8.31×10^{4}
YWHAZ (C)	3.30×10^{4}	2.39×10^{4}	4.68×10^{4}	5.49×10^{4}	1.07×10^{5}	1.39×10^{5}

raw abundance: 1×10^{7}, 8×10^{6}, 6×10^{6}, 4×10^{6}, 2×10^{6}, 0

TABLE S6. Heat map summarizing the raw abundances of proteins trapped by c-IBD-SBP-YAP1C upon treatment of Flp-In T-REx 293 cells with different H_2O_2 concentrations for 10 min. DTT eluates of the experiment shown in Fig. S10 were processed for LC-MS/MS analysis. After validation, proteins enriched 2.5-fold or more in at least one of the H_2O_2-treated conditions were retrieved, named according to their UniProtKB gene name, and sorted in alphabetical order. The primary subcellular localizations of the protein (according to the same database) are indicated between brackets. The values represent raw abundances and are color-coded (with white and blue being low and high, respectively). ???, no specified localization; C, cytosol; CS, cytoskeleton; EC, extracellular; EE, early endosome; ER, endoplasmic reticulum; ES, endosomes; GA, Golgi apparatus; LY, lysosome; MT, mitochondria; NU, nucleus; PM, plasma membrane; RE, recycling endosome.

Table S6
(page 1)

external-H_2O_2 / c-YAP1C / 10 min

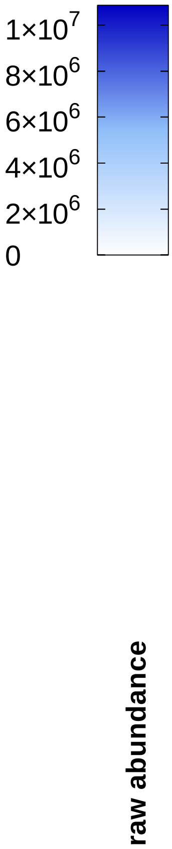

	0	10	30	100	300	1000
AAMDC (C)	5.66×10^{4}	4.59×10^{4}	5.82×10^{4}	1.31×10^{5}	1.44×10^{5}	2.05×10^{5}
AASDHPPT (C)	9.52×10^{4}	1.55×10^{5}	6.20×10^{5}	1.10×10^{6}	9.46×10^{5}	8.45×10^{5}
ACP1 (C)	4.63×10^{4}	5.61×10^{4}	6.09×10^{4}	9.36×10^{4}	1.43×10^{5}	2.61×10^{5}
ACP6 (MT)	0	0	1.29×10^{3}	3.61×10^{3}	1.96×10^{4}	6.24×10^{4}
ACT (???)	3.49×10^{3}	1.00×10^{4}	2.78×10^{4}	2.85×10^{4}	7.03×10^{4}	5.80×10^{4}
ACTN1 (C/PM)	6.44×10^{4}	9.06×10^{4}	2.00×10^{5}	3.47×10^{5}	2.92×10^{5}	3.24×10^{5}
ACYP2 (?)	4.64×10^{1}	3.57×10^{2}	4.31×10^{3}	1.09×10^{3}	7.28×10^{2}	2.44×10^{3}
ADH5 (C)	9.32×10^{4}	9.22×10^{4}	1.48×10^{5}	2.34×10^{5}	1.91×10^{5}	1.15×10^{5}
ADO (C)	2.06×10^{4}	2.49×10^{4}	7.82×10^{4}	1.01×10^{5}	9.28×10^{4}	1.02×10^{5}
AHCY (C)	1.80×10^{6}	2.24×10^{6}	3.05×10^{6}	7.48×10^{6}	6.94×10^{6}	7.06×10^{6}
AIF1L (CS/PM)	1.32×10^{3}	2.41×10^{3}	2.88×10^{4}	5.13×10^{4}	4.03×10^{4}	5.05×10^{4}
AK2 (MT)	2.54×10^{5}	3.55×10^{5}	6.33×10^{5}	1.26×10^{6}	1.19×10^{6}	1.98×10^{6}
AKAP12 (CS/PM)	4.22×10^{5}	4.96×10^{5}	7.01×10^{5}	1.35×10^{6}	1.13×10^{6}	9.40×10^{5}
AMPD2 (C)	2.96×10^{4}	3.03×10^{4}	6.46×10^{4}	1.09×10^{5}	1.05×10^{5}	9.06×10^{4}
ANP32B (C/NU)	0	1.07×10^{2}	1.82×10^{3}	4.18×10^{3}	3.07×10^{3}	1.11×10^{4}
ANXA1 (C/NU/PM/EC)	1.81×10^{4}	1.71×10^{4}	1.35×10^{4}	3.35×10^{4}	2.08×10^{4}	1.41×10^{5}
ANXA2 (PM/EC)	4.67×10^{6}	6.87×10^{6}	1.78×10^{7}	2.45×10^{7}	2.09×10^{7}	2.00×10^{7}
APIP (C)	1.08×10^{4}	2.56×10^{4}	3.93×10^{4}	6.00×10^{4}	6.72×10^{4}	6.08×10^{4}
ARFGAP1 (GA/C)	9.56×10^{3}	1.45×10^{4}	7.00×10^{4}	1.23×10^{5}	9.87×10^{4}	1.06×10^{5}
ARL6IP4 (NU)	0	0	2.88×10^{4}	5.74×10^{4}	5.37×10^{4}	8.61×10^{4}
ASF1A (NU)	1.00×10^{4}	6.18×10^{4}	2.96×10^{5}	4.60×10^{5}	4.81×10^{5}	4.77×10^{5}
ASF1B (NU)	1.28×10^{5}	2.43×10^{5}	7.00×10^{5}	1.15×10^{6}	9.31×10^{5}	7.64×10^{5}
ASNS (C)	2.09×10^{4}	2.26×10^{4}	1.61×10^{4}	1.14×10^{5}	3.43×10^{5}	6.26×10^{5}
ASRGL1 (C)	0	6.26×10^{3}	1.98×10^{4}	5.78×10^{4}	5.42×10^{4}	6.21×10^{4}
ATG3 (C)	4.09×10^{4}	4.74×10^{4}	5.90×10^{4}	1.46×10^{5}	8.48×10^{4}	1.07×10^{5}
ATOX1 (C)	6.31×10^{3}	9.52×10^{3}	2.31×10^{4}	4.08×10^{4}	4.75×10^{4}	1.24×10^{5}
ATP5F1C (MT)	7.04×10^{3}	1.09×10^{4}	1.20×10^{4}	2.16×10^{4}	1.74×10^{4}	1.36×10^{4}
ATP6V1B1 (PM)	1.70×10^{5}	2.19×10^{5}	2.67×10^{5}	4.87×10^{5}	4.57×10^{5}	4.78×10^{5}
BAG3 (C/NU)	5.03×10^{4}	7.98×10^{4}	3.22×10^{5}	6.99×10^{5}	5.13×10^{5}	5.48×10^{5}
BAG4 (C)	5.37×10^{3}	1.32×10^{4}	3.51×10^{4}	1.05×10^{5}	9.49×10^{4}	6.90×10^{4}
BAG5 (C/NU/MT)	7.34×10^{4}	1.98×10^{5}	1.08×10^{6}	1.58×10^{6}	1.41×10^{6}	1.34×10^{6}
BCAP31 (ER)	1.74×10^{5}	2.14×10^{5}	2.25×10^{5}	4.40×10^{5}	3.38×10^{5}	2.27×10^{5}
BICD2 (C/NU/CS/GA)	6.56×10^{2}	2.48×10^{4}	4.29×10^{4}	7.68×10^{4}	6.52×10^{4}	8.22×10^{4}
BID (C/MT)	5.74×10^{4}	2.34×10^{5}	4.98×10^{5}	8.55×10^{5}	7.53×10^{5}	8.61×10^{5}
BOLA1 (MT)	6.09×10^{4}	7.13×10^{4}	2.56×10^{5}	2.94×10^{5}	2.30×10^{5}	2.16×10^{5}
BOLA2B (C/NU)	1.49×10^{6}	1.58×10^{6}	6.39×10^{6}	5.92×10^{6}	4.42×10^{6}	6.05×10^{6}
C11orf54 (NU)	1.69×10^{4}	3.07×10^{4}	1.54×10^{5}	1.94×10^{5}	1.88×10^{5}	1.53×10^{5}
C11orf58 (???)	6.56×10^{4}	1.61×10^{5}	1.60×10^{6}	2.56×10^{6}	1.46×10^{6}	2.04×10^{6}
C1orf174 (NU)	0	0	0	0	4.62×10^{2}	1.68×10^{3}
C1orf52 (NU)	5.81×10^{3}	7.76×10^{3}	5.91×10^{4}	1.36×10^{5}	1.19×10^{5}	1.35×10^{5}
CACYBP (C/NU)	1.06×10^{6}	1.55×10^{6}	3.91×10^{6}	6.73×10^{6}	5.70×10^{6}	5.32×10^{6}
CALD1 (CS)	1.20×10^{5}	1.77×10^{5}	3.44×10^{5}	8.82×10^{5}	6.43×10^{5}	4.05×10^{5}
CAPZA2 (C/CS/EC)	5.97×10^{4}	1.23×10^{5}	1.33×10^{5}	2.29×10^{5}	2.46×10^{5}	1.83×10^{5}
CARHSP1 (C)	6.32×10^{5}	6.67×10^{5}	9.60×10^{5}	1.64×10^{6}	1.31×10^{6}	1.45×10^{6}
CASP3 (C)	2.25×10^{5}	3.27×10^{5}	5.20×10^{5}	7.18×10^{5}	7.13×10^{5}	6.64×10^{5}
CAST(C/ER)	4.55×10^{5}	1.14×10^{6}	7.40×10^{6}	1.14×10^{7}	9.43×10^{6}	8.88×10^{6}
CCDC50 (C)	2.02×10^{4}	1.04×10^{5}	3.35×10^{5}	5.44×10^{5}	4.09×10^{5}	3.86×10^{5}
CCDC6 (C/CS)	2.37×10^{5}	3.70×10^{5}	1.33×10^{6}	2.69×10^{6}	2.18×10^{6}	1.97×10^{6}
CCS (C)	2.01×10^{5}	2.36×10^{5}	5.09×10^{5}	1.04×10^{6}	1.13×10^{6}	1.19×10^{6}
CDK4 (C/NU)	3.28×10^{5}	1.57×10^{6}	2.99×10^{6}	4.88×10^{6}	4.37×10^{6}	3.60×10^{6}

Table S6
(page 2)

external-H_2O_2 / c-YAP1C / 10 min

raw abundance: 1×10^{7}, 8×10^{6}, 6×10^{6}, 4×10^{6}, 2×10^{6}, 0

0	10	30	100	300	1000	
2.17×10^{4}	2.92×10^{4}	1.21×10^{5}	2.29×10^{5}	1.69×10^{5}	1.62×10^{5}	CDK6(C/NU/CS)
7.07×10^{5}	1.60×10^{6}	3.15×10^{6}	3.98×10^{6}	3.79×10^{6}	3.43×10^{6}	CDKN2A (C/NU)
1.57×10^{4}	4.57×10^{4}	7.87×10^{4}	1.49×10^{5}	1.21×10^{5}	1.38×10^{5}	CHAC2 (C)
4.34×10^{5}	4.95×10^{5}	9.87×10^{5}	1.15×10^{6}	8.28×10^{5}	9.70×10^{5}	CHCHD4 (MT)
2.08×10^{4}	4.21×10^{4}	2.97×10^{4}	5.26×10^{4}	4.40×10^{4}	3.85×10^{4}	CHMP2B (C/ES)
5.54×10^{4}	9.32×10^{4}	2.98×10^{5}	7.92×10^{5}	6.70×10^{5}	5.18×10^{5}	CHMP5 (C/ES)
3.49×10^{4}	3.93×10^{4}	5.66×10^{4}	8.79×10^{4}	9.06×10^{4}	6.60×10^{4}	CIAO2A (C/NU)
1.19×10^{6}	3.48×10^{6}	1.67×10^{7}	2.65×10^{7}	2.42×10^{7}	2.57×10^{7}	CIAPIN1 (C/NU/MT)
3.28×10^{3}	2.14×10^{3}	4.71×10^{3}	1.61×10^{4}	3.21×10^{4}	7.01×10^{4}	CKMT1A (MT)
1.18×10^{5}	1.37×10^{5}	1.17×10^{5}	1.48×10^{6}	3.79×10^{5}	6.04×10^{5}	CLU (C)
9.86×10^{5}	1.61×10^{6}	1.52×10^{6}	2.78×10^{6}	2.46×10^{6}	1.98×10^{6}	CNN3 (C/CS)
1.14×10^{5}	4.71×10^{4}	4.09×10^{5}	1.95×10^{5}	9.25×10^{4}	2.62×10^{5}	COX17 (C/MT)
5.09×10^{5}	1.01×10^{6}	2.05×10^{6}	3.52×10^{6}	3.98×10^{6}	5.88×10^{6}	CPOX (MT)
1.57×10^{3}	2.62×10^{4}	8.86×10^{4}	1.22×10^{5}	1.32×10^{5}	1.79×10^{5}	CPPED1 (C)
1.36×10^{6}	1.89×10^{6}	1.01×10^{7}	1.71×10^{7}	1.59×10^{7}	1.51×10^{7}	CRKL (C/NU/PM)
1.64×10^{4}	8.02×10^{4}	2.08×10^{5}	3.64×10^{5}	3.71×10^{5}	2.87×10^{5}	CRMP1 (C/CS)
1.77×10^{4}	6.95×10^{4}	1.28×10^{5}	1.74×10^{5}	1.68×10^{5}	1.60×10^{5}	CRYZL1 (C)
7.85×10^{5}	1.71×10^{6}	1.33×10^{7}	1.18×10^{7}	8.26×10^{6}	1.57×10^{7}	CSTB (C/NU)
3.39×10^{4}	4.89×10^{4}	2.35×10^{5}	5.99×10^{5}	6.67×10^{5}	8.13×10^{5}	CTH (C)
4.07×10^{6}	4.80×10^{6}	1.09×10^{7}	8.74×10^{6}	5.96×10^{6}	6.65×10^{6}	CTSB (LY/EC)
3.28×10^{6}	4.24×10^{6}	7.27×10^{6}	1.38×10^{7}	1.19×10^{7}	9.38×10^{6}	CTTN (CS/PM)
1.96×10^{0}	1.07×10^{3}	1.76×10^{4}	5.18×10^{4}	3.84×10^{4}	5.18×10^{4}	CWC15 (NU)
8.47×10^{4}	9.56×10^{4}	1.04×10^{5}	2.12×10^{5}	2.00×10^{5}	1.41×10^{5}	CYB5R3 (C/MT/ER)
1.94×10^{5}	2.86×10^{5}	2.69×10^{5}	4.99×10^{5}	4.14×10^{5}	3.76×10^{5}	DBNL (C/GA/PM/EE/CS)
1.53×10^{4}	1.47×10^{4}	1.48×10^{5}	2.39×10^{5}	2.16×10^{5}	2.29×10^{5}	DCPS (C/NU)
3.38×10^{5}	8.84×10^{5}	4.17×10^{6}	6.63×10^{6}	6.44×10^{6}	5.53×10^{6}	DCTPP1 (C/NU/MT)
1.09×10^{4}	3.44×10^{4}	5.61×10^{4}	1.96×10^{5}	1.77×10^{5}	2.06×10^{5}	DENR (???)
2.25×10^{5}	3.95×10^{5}	5.05×10^{5}	9.70×10^{5}	8.98×10^{5}	6.40×10^{5}	DFFA (C)
3.88×10^{4}	6.60×10^{4}	2.94×10^{5}	4.04×10^{5}	3.57×10^{5}	4.32×10^{5}	DHPS (C)
3.12×10^{3}	6.05×10^{3}	6.11×10^{3}	2.91×10^{4}	1.81×10^{4}	1.88×10^{4}	DLAT (MT)
1.39×10^{5}	5.96×10^{5}	2.69×10^{6}	5.39×10^{6}	6.09×10^{6}	7.63×10^{6}	DNPEP (C)
2.66×10^{6}	4.64×10^{6}	4.80×10^{6}	8.89×10^{6}	7.39×10^{6}	5.35×10^{6}	DPYSL2 (C/CS/PM)
7.76×10^{5}	2.13×10^{6}	5.57×10^{6}	1.06×10^{7}	9.16×10^{6}	7.32×10^{6}	DPYSL3 (C)
7.38×10^{4}	9.79×10^{4}	2.04×10^{5}	3.84×10^{5}	3.84×10^{5}	2.33×10^{5}	DPYSL5 (C)
1.21×10^{3}	3.22×10^{2}	4.01×10^{2}	2.88×10^{3}	3.46×10^{3}	1.99×10^{3}	DRG1 (C/NU)
2.90×10^{4}	4.77×10^{4}	4.23×10^{4}	1.33×10^{5}	2.02×10^{5}	2.73×10^{5}	DUSP3 (NU)
1.40×10^{5}	2.50×10^{5}	7.81×10^{5}	1.16×10^{6}	7.20×10^{5}	7.76×10^{5}	DYNLL1 (NU/CS/MT)
1.21×10^{4}	2.17×10^{4}	1.49×10^{5}	2.75×10^{5}	1.81×10^{5}	2.12×10^{5}	ECI1 (MT)
8.22×10^{4}	2.35×10^{5}	6.89×10^{5}	1.09×10^{6}	9.53×10^{5}	1.10×10^{6}	EFHD2(PM)
1.11×10^{4}	1.78×10^{4}	1.06×10^{5}	1.66×10^{5}	1.76×10^{5}	1.97×10^{5}	EIF1AD (NU)
1.24×10^{3}	2.88×10^{3}	3.16×10^{3}	7.98×10^{3}	1.17×10^{4}	4.71×10^{3}	EIF3B (C)
2.18×10^{5}	5.85×10^{5}	3.06×10^{5}	6.46×10^{5}	6.49×10^{5}	5.18×10^{5}	EIF3G (C/NU)
5.30×10^{4}	8.59×10^{4}	7.04×10^{4}	1.14×10^{5}	1.33×10^{5}	1.09×10^{5}	EIF3I (C)
3.17×10^{1}	7.16×10^{1}	7.38×10^{1}	4.73×10^{0}	8.58×10^{1}	2.37×10^{1}	EIF4A2 (C)
2.07×10^{4}	4.08×10^{4}	1.86×10^{5}	3.69×10^{5}	3.13×10^{5}	4.47×10^{5}	EIF4B (C)
3.94×10^{3}	3.89×10^{4}	1.53×10^{5}	1.03×10^{5}	4.67×10^{4}	5.29×10^{4}	EIF4EBP1 (C/NU)
1.50×10^{6}	1.40×10^{6}	1.62×10^{6}	4.35×10^{6}	3.29×10^{6}	2.69×10^{6}	EIF5A (C/NU/ER)
1.26×10^{5}	1.70×10^{5}	4.59×10^{5}	6.46×10^{5}	5.26×10^{5}	4.33×10^{5}	EIF6 (C/NU)
1.62×10^{4}	3.59×10^{4}	3.50×10^{4}	8.01×10^{4}	6.36×10^{4}	5.95×10^{4}	EPS15L1 (NU/PM)
8.55×10^{4}	2.58×10^{4}	4.77×10^{5}	6.99×10^{5}	6.46×10^{5}	7.32×10^{5}	ERICH5 (???)

Table S6
(page 3)

external-H_2O_2 / c-YAP1C / 10 min

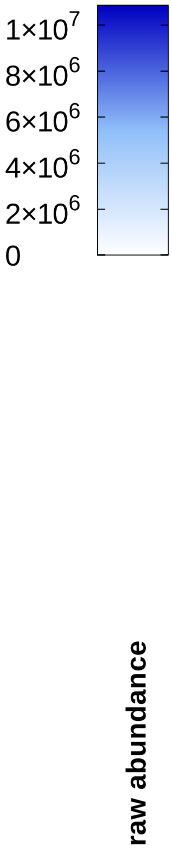

0	10	30	100	300	1000	
9.03×10^{4}	3.01×10^{5}	6.04×10^{5}	9.12×10^{5}	6.05×10^{5}	5.05×10^{5}	EWSR1 (C/NU/PM)
1.05×10^{4}	2.75×10^{4}	1.22×10^{4}	4.99×10^{4}	3.67×10^{4}	2.99×10^{4}	FAF1 (NU)
1.80×10^{3}	6.13×10^{3}	4.06×10^{4}	5.20×10^{4}	5.33×10^{4}	6.28×10^{4}	FAH (C/EC)
1.53×10^{4}	3.73×10^{4}	9.76×10^{3}	1.77×10^{4}	4.47×10^{4}	2.49×10^{4}	FKBP3 (NU)
1.12×10^{4}	4.33×10^{4}	5.18×10^{4}	7.47×10^{4}	6.59×10^{4}	7.99×10^{4}	FLYWCH2 (???)
2.12×10^{5}	5.74×10^{5}	1.28×10^{6}	2.08×10^{6}	1.67×10^{6}	1.68×10^{6}	FN3KRP (C)
3.31×10^{3}	1.58×10^{4}	8.42×10^{4}	1.57×10^{5}	1.07×10^{5}	9.30×10^{4}	FNTA (C/PM/CS)
1.58×10^{4}	3.48×10^{4}	1.52×10^{5}	2.26×10^{5}	1.95×10^{5}	2.23×10^{5}	FNTB (C/CS)
1.59×10^{6}	2.10×10^{6}	2.72×10^{6}	4.37×10^{6}	3.64×10^{6}	2.44×10^{6}	FSCN1 (C/CS)
2.20×10^{4}	5.22×10^{4}	1.58×10^{5}	2.27×10^{5}	2.00×10^{5}	2.22×10^{5}	FTH1 (C/NU/LY/EC)
2.24×10^{5}	2.61×10^{5}	3.34×10^{5}	5.82×10^{5}	4.93×10^{5}	4.37×10^{5}	GARS1 (C/EC)
4.41×10^{4}	1.38×10^{5}	2.35×10^{5}	4.37×10^{5}	3.62×10^{5}	3.47×10^{5}	GCLC (C)
8.06×10^{4}	2.93×10^{5}	8.95×10^{5}	1.48×10^{6}	1.10×10^{6}	9.80×10^{5}	GCLM (C)
1.40×10^{5}	2.15×10^{5}	2.37×10^{5}	5.03×10^{5}	4.11×10^{5}	3.73×10^{5}	GEMIN6 (C/NU)
7.74×10^{4}	1.73×10^{5}	3.90×10^{5}	7.52×10^{5}	6.56×10^{5}	9.19×10^{5}	GFER (C/MT/EC)
4.39×10^{3}	9.36×10^{3}	2.19×10^{4}	6.31×10^{4}	6.36×10^{4}	6.21×10^{4}	GLDC (MT)
8.58×10^{4}	2.93×10^{5}	8.74×10^{5}	1.24×10^{6}	1.04×10^{6}	1.15×10^{6}	GLOD4 (MT)
2.83×10^{4}	4.08×10^{4}	9.66×10^{4}	1.72×10^{5}	1.65×10^{5}	1.64×10^{5}	GLRX3 (C)
3.81×10^{5}	6.27×10^{5}	7.33×10^{5}	1.52×10^{6}	1.93×10^{6}	1.60×10^{6}	GLRX5 (MT)
3.64×10^{5}	1.65×10^{6}	5.42×10^{6}	9.09×10^{6}	7.80×10^{6}	7.13×10^{6}	GPHN (C/PM)
4.78×10^{4}	6.60×10^{4}	1.29×10^{5}	2.52×10^{5}	2.14×10^{5}	2.24×10^{5}	GPX1 (C)
4.55×10^{5}	7.81×10^{5}	1.65×10^{6}	2.42×10^{6}	2.15×10^{6}	2.14×10^{6}	GPX4 (C/MT)
5.22×10^{4}	7.58×10^{4}	2.21×10^{5}	4.85×10^{5}	4.25×10^{5}	2.97×10^{5}	GRIPAP1 (ES)
4.16×10^{3}	4.15×10^{4}	5.49×10^{3}	1.28×10^{4}	1.32×10^{4}	1.34×10^{4}	GRN (LY/EC)
1.18×10^{4}	2.27×10^{4}	2.50×10^{4}	5.52×10^{4}	5.02×10^{4}	6.43×10^{4}	GSN (CS/EC)
1.77×10^{6}	5.79×10^{6}	3.20×10^{7}	4.67×10^{7}	4.30×10^{7}	3.78×10^{7}	GSR (C/MT)
1.59×10^{5}	2.91×10^{5}	4.09×10^{5}	7.22×10^{5}	6.79×10^{5}	7.46×10^{5}	GSTO1 (C)
2.96×10^{4}	5.47×10^{4}	7.86×10^{4}	1.40×10^{5}	1.22×10^{5}	1.09×10^{5}	H2BC11 (NU)
6.95×10^{4}	8.05×10^{4}	1.96×10^{5}	2.87×10^{5}	2.64×10^{5}	2.30×10^{5}	HAT1 (C/NU/MT)
1.69×10^{5}	1.43×10^{4}	1.01×10^{4}	2.19×10^{4}	4.90×10^{4}	3.91×10^{6}	HBA2 (C/EC)
1.80×10^{5}	3.03×10^{4}	2.85×10^{4}	5.28×10^{4}	8.15×10^{4}	7.85×10^{5}	HBB (C/EC)
3.72×10^{3}	2.57×10^{3}	4.04×10^{3}	9.76×10^{3}	4.92×10^{3}	4.58×10^{3}	HDAC2 (C/NU)
7.22×10^{4}	7.54×10^{4}	7.04×10^{5}	1.20×10^{6}	9.69×10^{5}	1.27×10^{6}	HDGF (NU)
3.97×10^{4}	9.84×10^{4}	1.79×10^{5}	3.79×10^{5}	2.41×10^{5}	2.28×10^{5}	HEXIM1 (C/NU)
2.06×10^{3}	5.97×10^{3}	2.40×10^{4}	5.60×10^{4}	3.94×10^{4}	3.53×10^{4}	HIRIP3 (NU)
8.54×10^{3}	1.58×10^{4}	1.60×10^{4}	3.12×10^{4}	2.17×10^{4}	2.01×10^{4}	HMGA1 (NU)
1.58×10^{5}	2.07×10^{5}	3.11×10^{5}	4.66×10^{5}	3.51×10^{5}	2.37×10^{5}	HMGCS1 (C)
9.88×10^{5}	3.98×10^{6}	1.35×10^{7}	2.09×10^{7}	2.15×10^{7}	2.54×10^{7}	HPRT1 (C)
1.67×10^{6}	2.68×10^{6}	9.94×10^{6}	2.17×10^{7}	2.08×10^{7}	2.18×10^{7}	HSD17B10 (MT)
2.17×10^{3}	1.04×10^{4}	5.63×10^{4}	9.99×10^{4}	1.10×10^{5}	1.12×10^{5}	HSD17B8 (MT)
1.71×10^{5}	1.50×10^{5}	2.56×10^{5}	4.33×10^{5}	3.81×10^{5}	3.79×10^{5}	HSPA4L (C/NU)
4.64×10^{3}	3.03×10^{4}	1.14×10^{5}	1.98×10^{5}	1.97×10^{5}	2.94×10^{5}	IAH1 (???)
0	2.34×10^{4}	5.96×10^{4}	1.28×10^{5}	1.27×10^{5}	1.34×10^{5}	IBA57 (MT)
2.05×10^{4}	2.16×10^{4}	3.58×10^{4}	7.24×10^{4}	4.94×10^{4}	4.24×10^{4}	IKBKG (C/NU)
1.51×10^{5}	2.98×10^{5}	5.82×10^{5}	1.18×10^{6}	1.06×10^{6}	1.17×10^{6}	IMPDH2 (C/NU)
1.13×10^{5}	2.23×10^{5}	4.01×10^{5}	9.51×10^{5}	9.23×10^{5}	6.56×10^{5}	ISCA2 (MT)
6.77×10^{4}	1.20×10^{5}	2.87×10^{5}	7.78×10^{5}	6.41×10^{5}	9.01×10^{5}	ISCU (C/NU/MT)
3.64×10^{6}	4.45×10^{6}	1.58×10^{7}	2.15×10^{7}	2.14×10^{7}	2.30×10^{7}	ISYNA1 (C)
3.27×10^{4}	4.02×10^{4}	8.65×10^{4}	1.48×10^{5}	1.26×10^{5}	9.19×10^{4}	JMJD6 (C/NU)
2.66×10^{5}	1.51×10^{6}	8.23×10^{6}	8.55×10^{6}	4.94×10^{6}	8.12×10^{6}	JPT2 (C/NU)

Table S6 (page 4)

external-H_2O_2 / c-YAP1C / 10 min

raw abundance (colour scale): 1×10^{7}, 8×10^{6}, 6×10^{6}, 4×10^{6}, 2×10^{6}, 0

0	10	30	100	300	1000	
1.06×10^{3}	1.05×10^{4}	3.93×10^{4}	6.88×10^{4}	5.08×10^{4}	5.02×10^{4}	KCTD5 (C/NU)
2.47×10^{4}	1.96×10^{4}	4.04×10^{4}	6.75×10^{4}	4.66×10^{4}	1.81×10^{4}	KHDRBS1 (C/NU/PM)
1.79×10^{4}	5.48×10^{4}	1.00×10^{5}	2.05×10^{5}	1.73×10^{5}	1.52×10^{5}	KHSRP (C/NU)
4.26×10^{4}	7.01×10^{4}	1.04×10^{5}	1.39×10^{5}	1.41×10^{5}	1.22×10^{5}	KIF3A (CS)
6.35×10^{3}	9.50×10^{3}	5.05×10^{4}	9.99×10^{4}	9.43×10^{4}	1.06×10^{5}	LARP7 (NU)
9.69×10^{3}	3.38×10^{4}	2.01×10^{5}	2.19×10^{5}	1.70×10^{5}	1.15×10^{5}	LBH (C/NU)
7.90×10^{5}	7.01×10^{5}	2.80×10^{6}	4.46×10^{6}	3.83×10^{6}	3.86×10^{6}	LCP1 (PM/CS)
2.50×10^{4}	6.44×10^{4}	1.58×10^{5}	2.93×10^{5}	3.08×10^{5}	3.11×10^{5}	LRRFIP1 (C/NU)
3.20×10^{4}	1.27×10^{5}	1.87×10^{5}	4.65×10^{5}	2.96×10^{5}	3.51×10^{5}	LYRM7 (MT)
8.13×10^{4}	1.31×10^{5}	1.42×10^{5}	2.10×10^{5}	1.69×10^{5}	2.11×10^{5}	MAD2L1(C/CS/NU)
3.05×10^{5}	3.64×10^{5}	1.23×10^{6}	2.19×10^{6}	1.85×10^{6}	1.80×10^{6}	MAP4 (CS)
1.76×10^{4}	8.93×10^{3}	3.53×10^{4}	6.89×10^{4}	6.34×10^{4}	4.59×10^{4}	MAPK14 (C/NU)
5.64×10^{5}	1.53×10^{6}	2.78×10^{6}	6.35×10^{6}	5.02×10^{6}	4.21×10^{6}	MARCKS (CS/PM)
6.71×10^{4}	3.29×10^{5}	1.39×10^{6}	3.46×10^{6}	2.64×10^{6}	2.84×10^{6}	MARCKSL1(CS/PM)
3.64×10^{3}	1.72×10^{4}	4.51×10^{4}	9.06×10^{4}	7.36×10^{4}	1.54×10^{5}	MCMBP (NU)
2.13×10^{5}	2.22×10^{5}	7.48×10^{5}	1.16×10^{6}	9.44×10^{5}	9.73×10^{5}	MDH1 (C)
4.24×10^{3}	2.91×10^{4}	4.61×10^{4}	6.86×10^{4}	5.46×10^{4}	4.03×10^{4}	METTL26 (???)
1.11×10^{5}	9.48×10^{4}	2.77×10^{5}	4.53×10^{5}	5.25×10^{5}	4.17×10^{5}	MINDY3 (NU)
2.41×10^{4}	3.23×10^{4}	6.75×10^{4}	1.29×10^{5}	1.41×10^{5}	1.58×10^{5}	MMAB (MT)
3.50×10^{4}	7.29×10^{4}	2.24×10^{5}	3.96×10^{5}	3.38×10^{5}	3.18×10^{5}	MOCS2 (C)
7.02×10^{3}	2.27×10^{4}	6.20×10^{4}	1.53×10^{5}	1.38×10^{5}	1.01×10^{5}	MPP1 (PM)
0	0	6.94×10^{2}	7.72×10^{2}	2.55×10^{3}	3.67×10^{2}	MRPL2 (MT)
4.93×10^{3}	3.93×10^{4}	9.31×10^{4}	1.53×10^{5}	1.37×10^{5}	1.36×10^{5}	MSRB3 (MT/ER)
6.07×10^{4}	1.06×10^{5}	3.28×10^{5}	5.41×10^{5}	5.64×10^{5}	4.30×10^{5}	MTAP (C/NU)
9.19×10^{4}	1.57×10^{5}	1.83×10^{5}	4.18×10^{5}	2.94×10^{5}	2.88×10^{5}	NAA10 (C/NU)
2.78×10^{6}	3.44×10^{6}	4.62×10^{6}	7.06×10^{6}	6.46×10^{6}	5.97×10^{6}	NASP (C/NU)
1.93×10^{5}	2.14×10^{5}	6.51×10^{5}	1.05×10^{6}	8.34×10^{5}	6.78×10^{5}	NDUFAB1 (MT)
3.22×10^{5}	3.83×10^{5}	7.07×10^{5}	9.01×10^{5}	7.05×10^{5}	8.71×10^{5}	NDUFS6 (MT)
4.76×10^{4}	6.70×10^{4}	9.44×10^{4}	2.22×10^{5}	1.09×10^{5}	1.09×10^{5}	NDUFV3 (MT)
4.95×10^{5}	7.11×10^{5}	3.63×10^{6}	9.03×10^{6}	7.79×10^{6}	7.47×10^{6}	NFU1 (C/MT)
2.26×10^{6}	2.17×10^{6}	5.93×10^{6}	9.79×10^{6}	8.57×10^{6}	7.65×10^{6}	NPEPPS (C/NU)
1.18×10^{5}	1.56×10^{5}	1.83×10^{5}	2.94×10^{5}	2.98×10^{5}	2.85×10^{5}	NRDC (C/MT)
1.29×10^{4}	3.14×10^{4}	1.32×10^{5}	4.15×10^{5}	3.46×10^{5}	3.30×10^{5}	NT5DC2 (???)
8.17×10^{5}	4.45×10^{6}	1.57×10^{7}	2.56×10^{7}	2.23×10^{7}	2.37×10^{7}	NUDC (CS/NU)
8.99×10^{3}	3.04×10^{4}	2.54×10^{4}	3.40×10^{4}	3.57×10^{4}	4.73×10^{4}	NUDT1 (C/NU/MT)
2.22×10^{4}	8.22×10^{4}	8.94×10^{5}	1.37×10^{6}	1.20×10^{6}	1.25×10^{6}	OGFR (C/NU)
9.16×10^{3}	6.42×10^{4}	9.36×10^{4}	1.68×10^{5}	1.49×10^{5}	1.36×10^{5}	OPTN (C/GA/RE)
2.63×10^{4}	3.43×10^{4}	2.94×10^{4}	1.90×10^{5}	4.49×10^{5}	1.22×10^{6}	OTUB1 (C)
7.66×10^{4}	8.01×10^{4}	1.30×10^{5}	2.40×10^{5}	1.63×10^{5}	1.38×10^{5}	PABIR2 (???)
7.78×10^{4}	1.12×10^{5}	1.97×10^{5}	3.44×10^{5}	3.35×10^{5}	4.05×10^{5}	PABPC1 (C/NU)
3.70×10^{3}	4.04×10^{4}	9.42×10^{4}	1.92×10^{5}	1.94×10^{5}	2.07×10^{5}	PABPC4 (C)
6.48×10^{5}	1.35×10^{6}	2.66×10^{6}	5.82×10^{6}	4.99×10^{6}	2.91×10^{6}	PAICS (C/EC)
4.57×10^{4}	1.27×10^{5}	3.17×10^{5}	7.81×10^{5}	7.15×10^{5}	6.33×10^{5}	PAWR (C/NU)
6.22×10^{5}	1.39×10^{6}	3.19×10^{6}	5.21×10^{6}	4.70×10^{6}	4.61×10^{6}	PBK (NU)
9.18×10^{4}	1.67×10^{5}	6.29×10^{5}	1.45×10^{6}	1.23×10^{6}	9.36×10^{5}	PCBP1 (C/NU)
9.91×10^{4}	7.31×10^{4}	3.78×10^{5}	4.04×10^{5}	4.37×10^{5}	4.29×10^{5}	PCBP2 (C/NU)
3.01×10^{6}	5.48×10^{6}	1.31×10^{7}	2.01×10^{7}	1.95×10^{7}	1.79×10^{7}	PCMT1 (C)
8.67×10^{3}	1.55×10^{4}	3.55×10^{4}	7.53×10^{4}	5.32×10^{4}	4.04×10^{4}	PDCD6IP (C/CS)
2.43×10^{5}	1.76×10^{5}	2.66×10^{5}	6.88×10^{5}	9.32×10^{5}	1.13×10^{6}	PDHB (MT)
8.40×10^{6}	1.54×10^{7}	1.38×10^{7}	2.25×10^{7}	1.93×10^{7}	1.50×10^{7}	PDIA3 (ER)

Table S6
(page 5)

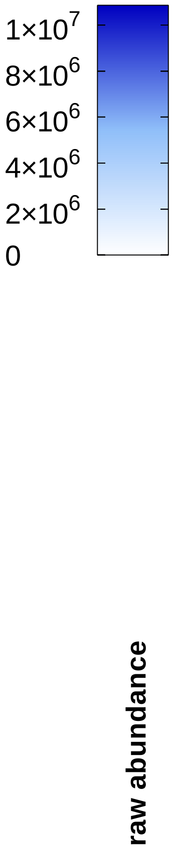

external-H_2O_2 / c-YAP1C / 10 min

0	10	30	100	300	1000	
7.23×10^{5}	1.06×10^{6}	7.23×10^{5}	1.94×10^{6}	1.45×10^{6}	1.09×10^{6}	PDLIM1 (C/CS)
3.29×10^{4}	2.88×10^{4}	5.75×10^{4}	8.80×10^{4}	6.43×10^{4}	8.12×10^{4}	PFDN1 (C)
1.29×10^{5}	2.88×10^{5}	1.17×10^{6}	1.61×10^{6}	1.39×10^{6}	1.79×10^{6}	PFDN2 (C/NU/MT)
6.33×10^{5}	1.26×10^{6}	3.54×10^{6}	5.06×10^{6}	5.05×10^{6}	4.73×10^{6}	PFDN4 (C/NU/MT)
5.77×10^{4}	1.16×10^{5}	5.67×10^{5}	7.81×10^{5}	6.36×10^{5}	8.09×10^{5}	PFDN5 (C/NU)
5.09×10^{5}	1.21×10^{6}	4.35×10^{6}	5.76×10^{6}	5.32×10^{6}	5.38×10^{6}	PFN2 (CS)
2.21×10^{5}	2.56×10^{5}	5.83×10^{5}	7.14×10^{5}	7.33×10^{5}	6.08×10^{5}	PGLS (C)
7.25×10^{3}	5.43×10^{4}	4.20×10^{5}	9.78×10^{5}	1.06×10^{6}	9.81×10^{5}	PHAX (C/NU)
1.47×10^{4}	7.28×10^{3}	1.28×10^{4}	4.81×10^{4}	1.11×10^{5}	2.01×10^{5}	PIN1 (C/NU)
2.41×10^{5}	1.01×10^{6}	9.40×10^{6}	1.42×10^{7}	1.19×10^{7}	1.01×10^{7}	PITHD1 (C)
8.84×10^{3}	1.26×10^{4}	3.16×10^{4}	6.85×10^{4}	7.69×10^{4}	1.04×10^{5}	PITRM1 (MT)
1.29×10^{6}	1.87×10^{6}	5.91×10^{6}	9.49×10^{6}	8.01×10^{6}	8.45×10^{6}	PLS3 (C)
3.40×10^{3}	3.56×10^{4}	2.83×10^{4}	8.58×10^{4}	4.15×10^{4}	4.23×10^{4}	PMPCB (MT)
2.44×10^{5}	5.38×10^{5}	2.04×10^{6}	2.87×10^{6}	2.52×10^{6}	2.78×10^{6}	PNPO (C)
2.84×10^{4}	4.65×10^{4}	6.87×10^{4}	1.64×10^{5}	1.24×10^{5}	1.43×10^{5}	POLDIP2 (NU/MT)
6.02×10^{3}	5.76×10^{4}	2.77×10^{5}	3.98×10^{5}	3.23×10^{5}	5.88×10^{5}	PPAT (C)
2.48×10^{4}	1.38×10^{5}	6.25×10^{5}	8.42×10^{5}	7.51×10^{5}	8.02×10^{5}	PPCDC (C)
2.15×10^{2}	0	4.39×10^{1}	0	0	9.10×10^{2}	PPL (C/CS/PM)
1.45×10^{3}	1.08×10^{3}	2.19×10^{3}	6.84×10^{3}	4.02×10^{3}	4.61×10^{3}	PPP1CC (C/NU/MT)
3.99×10^{5}	1.17×10^{6}	6.23×10^{6}	7.75×10^{6}	6.00×10^{6}	6.75×10^{6}	PPP1R11 (C/NU)
0	0	4.68×10^{4}	9.10×10^{4}	4.77×10^{4}	1.08×10^{5}	PPP1R2 (???)
2.85×10^{4}	3.64×10^{4}	1.15×10^{5}	1.57×10^{5}	1.49×10^{5}	1.38×10^{5}	PPP1R7 (NU)
4.58×10^{5}	2.15×10^{6}	1.04×10^{7}	1.57×10^{7}	1.36×10^{7}	1.38×10^{7}	PPP2R2A (C/NU)
2.43×10^{3}	2.82×10^{4}	1.72×10^{5}	2.58×10^{5}	2.35×10^{5}	2.30×10^{5}	PPP2R2D (C)
1.18×10^{5}	1.68×10^{5}	2.20×10^{5}	3.52×10^{5}	4.31×10^{5}	2.34×10^{5}	PPP5C (C/NU/PM)
5.42×10^{7}	2.36×10^{8}	7.01×10^{8}	8.78×10^{8}	7.59×10^{8}	6.49×10^{8}	PRDX1 (C)
1.17×10^{7}	3.36×10^{7}	8.60×10^{7}	1.02×10^{8}	8.59×10^{7}	5.30×10^{7}	PRDX2 (C)
7.04×10^{6}	1.88×10^{7}	4.45×10^{7}	7.60×10^{7}	5.08×10^{7}	2.62×10^{7}	PRDX3 (MT/C/EE)
6.44×10^{6}	1.05×10^{7}	1.71×10^{7}	2.79×10^{7}	2.38×10^{7}	2.05×10^{7}	PRDX4 (C/ER)
1.32×10^{6}	3.00×10^{6}	7.06×10^{6}	1.43×10^{7}	1.41×10^{7}	1.14×10^{7}	PRDX5 (C/NU/MT/PO)
2.75×10^{6}	7.30×10^{6}	1.42×10^{7}	3.10×10^{7}	2.95×10^{7}	2.35×10^{7}	PRDX6 (C/LY)
5.68×10^{4}	1.19×10^{5}	2.80×10^{5}	4.11×10^{5}	4.68×10^{5}	4.92×10^{5}	PREP (C)
6.30×10^{5}	1.94×10^{6}	4.20×10^{6}	6.67×10^{6}	6.25×10^{6}	6.80×10^{6}	PRMT5 (C/NU/GA)
7.59×10^{5}	3.87×10^{5}	1.75×10^{6}	2.52×10^{6}	2.02×10^{6}	2.11×10^{6}	PSMD9 (C/NU)
7.91×10^{5}	1.02×10^{6}	3.08×10^{6}	4.61×10^{6}	4.28×10^{6}	3.46×10^{6}	PSME3 (C/NU)
1.04×10^{4}	5.18×10^{4}	1.48×10^{5}	2.43×10^{5}	2.17×10^{5}	1.67×10^{5}	PSME3IP1 (NU)
5.51×10^{4}	1.13×10^{5}	5.05×10^{4}	1.19×10^{5}	1.55×10^{5}	1.03×10^{5}	PSMG4 (C)
1.42×10^{5}	3.19×10^{5}	5.20×10^{5}	8.34×10^{5}	7.63×10^{5}	6.63×10^{5}	RABEP2 (C/CS/ES)
2.95×10^{5}	5.98×10^{5}	2.05×10^{6}	3.08×10^{6}	2.67×10^{6}	2.87×10^{6}	RANBP3 (C/NU)
6.02×10^{5}	1.33×10^{6}	3.86×10^{6}	5.30×10^{6}	4.43×10^{6}	4.47×10^{6}	RBBP7 (NU)
1.11×10^{5}	1.39×10^{5}	3.36×10^{5}	5.36×10^{5}	4.91×10^{5}	5.27×10^{5}	RBM8A(C/NU)
9.14×10^{4}	1.25×10^{5}	1.80×10^{5}	2.55×10^{5}	1.95×10^{5}	1.81×10^{5}	RBMX (NU)
8.10×10^{4}	1.42×10^{5}	5.79×10^{5}	9.41×10^{5}	8.80×10^{5}	1.00×10^{6}	RCC2(NU/PM/CS)
1.64×10^{3}	2.29×10^{4}	6.19×10^{4}	9.33×10^{4}	8.36×10^{4}	1.32×10^{5}	RILPL1 (C/CS)
3.06×10^{4}	3.36×10^{4}	4.81×10^{4}	7.86×10^{4}	9.04×10^{4}	5.81×10^{4}	RNPS1 (C/NU)
1.70×10^{4}	3.84×10^{4}	1.58×10^{5}	2.61×10^{5}	2.59×10^{5}	2.67×10^{5}	RPIA(C)
8.05×10^{4}	1.39×10^{5}	1.06×10^{5}	1.54×10^{5}	1.29×10^{5}	2.11×10^{5}	RPL15 (PM)
2.58×10^{4}	3.74×10^{4}	6.71×10^{4}	1.55×10^{5}	8.12×10^{4}	7.50×10^{4}	RPL27 (C/ER)
2.08×10^{2}	3.06×10^{2}	3.60×10^{2}	4.53×10^{2}	6.80×10^{2}	1.77×10^{2}	RPL36A (C)
3.14×10^{5}	4.09×10^{5}	9.60×10^{5}	3.02×10^{6}	3.22×10^{6}	3.70×10^{6}	RRM2 (C)

external-H_2O_2 / c-YAP1C / 10 min

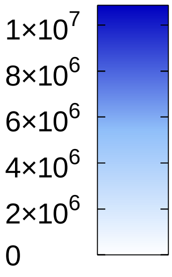

raw abundance

0	10	30	100	300	1000	
1.55×10^{4}	2.30×10^{4}	4.11×10^{4}	6.85×10^{4}	9.21×10^{4}	6.63×10^{4}	RRM2B (C/NU)
1.30×10^{5}	1.27×10^{5}	3.38×10^{5}	1.49×10^{5}	2.04×10^{5}	4.60×10^{5}	S100A9 (C/CS/PM/EC)
2.21×10^{2}	2.04×10^{2}	5.07×10^{2}	4.37×10^{2}	8.08×10^{2}	2.28×10^{3}	SAP18 (C/NU)
9.01×10^{3}	2.79×10^{4}	6.41×10^{4}	1.95×10^{5}	1.69×10^{5}	1.20×10^{5}	SDE2 (NU)
3.29×10^{4}	4.74×10^{4}	3.91×10^{4}	1.03×10^{5}	6.17×10^{4}	5.18×10^{4}	SEC22B (GA/ER)
1.45×10^{5}	1.67×10^{5}	7.40×10^{5}	1.16×10^{6}	1.05×10^{6}	1.07×10^{6}	SELENBP1 (C/NU)
3.03×10^{4}	3.20×10^{4}	4.41×10^{4}	7.16×10^{4}	6.03×10^{4}	1.32×10^{6}	SERPINA1 (ER/EC)
2.14×10^{5}	4.49×10^{5}	2.25×10^{6}	3.58×10^{6}	3.22×10^{6}	3.22×10^{6}	SERPINB1 (C/ES/LY/EC)
3.72×10^{3}	4.62×10^{3}	6.71×10^{3}	1.13×10^{4}	9.78×10^{3}	8.25×10^{3}	SF3B4 (NU)
1.26×10^{4}	1.10×10^{4}	3.96×10^{4}	6.15×10^{4}	4.48×10^{4}	4.30×10^{4}	SFN (C/NU/EC)
0	3.49×10^{2}	9.67×10^{3}	1.39×10^{4}	1.28×10^{4}	1.38×10^{4}	SH3BP5 (MT/VE)
2.22×10^{5}	4.09×10^{5}	9.50×10^{5}	2.42×10^{6}	2.37×10^{6}	2.37×10^{6}	SHTN1 (CS)
8.26×10^{5}	4.62×10^{6}	3.33×10^{7}	4.76×10^{7}	3.84×10^{7}	2.65×10^{7}	SKP1 (C/NU)
5.64×10^{4}	1.11×10^{5}	2.55×10^{5}	5.31×10^{5}	4.94×10^{5}	5.59×10^{5}	SLC9A3R (NU/PM)
1.55×10^{4}	9.95×10^{4}	2.57×10^{5}	8.67×10^{5}	7.04×10^{5}	5.30×10^{5}	SMAP1 (PM)
2.84×10^{5}	5.72×10^{5}	6.21×10^{5}	1.33×10^{6}	1.07×10^{6}	9.36×10^{5}	SNAP23 (PM)
5.83×10^{2}	6.22×10^{3}	1.23×10^{4}	2.82×10^{4}	2.23×10^{4}	2.51×10^{4}	SNAPIN (C/LY/GA/ES)
9.87×10^{3}	1.99×10^{4}	5.73×10^{4}	1.17×10^{5}	1.06×10^{5}	1.19×10^{5}	SNU13 (NU)
2.15×10^{4}	5.85×10^{4}	4.56×10^{4}	7.55×10^{4}	6.59×10^{4}	5.38×10^{4}	SNUPN (C/NU)
7.95×10^{3}	5.16×10^{3}	3.23×10^{4}	5.16×10^{4}	4.95×10^{4}	5.77×10^{4}	SOD2 (MT)
3.67×10^{4}	4.57×10^{4}	1.24×10^{5}	2.11×10^{5}	1.73×10^{5}	2.01×10^{5}	SORBS3 (NU/CS)
1.17×10^{5}	1.34×10^{5}	1.53×10^{5}	3.57×10^{5}	3.32×10^{5}	3.41×10^{5}	SPTAN1 (CS/PM)
1.14×10^{4}	1.89×10^{4}	3.49×10^{4}	5.27×10^{4}	7.22×10^{4}	8.54×10^{4}	SRSF10 (C/NU)
7.04×10^{4}	1.46×10^{5}	3.11×10^{5}	5.58×10^{5}	5.08×10^{5}	4.47×10^{5}	SRXN1 (C)
3.82×10^{5}	6.09×10^{5}	4.65×10^{5}	8.81×10^{5}	9.78×10^{5}	8.01×10^{5}	ST13 (C)
1.29×10^{6}	1.83×10^{6}	4.69×10^{6}	7.15×10^{6}	6.00×10^{6}	5.60×10^{6}	STRAP (C/NU)
8.00×10^{3}	3.66×10^{4}	2.97×10^{4}	6.14×10^{4}	5.84×10^{4}	4.48×10^{4}	STRIP1 (C)
6.60×10^{4}	1.34×10^{5}	1.12×10^{5}	1.86×10^{5}	1.27×10^{5}	1.77×10^{5}	SUMF1 (ER)
2.41×10^{2}	8.37×10^{2}	2.37×10^{3}	5.77×10^{3}	2.89×10^{3}	9.12×10^{2}	SUMF2 (ER)
8.20×10^{4}	1.20×10^{5}	4.13×10^{5}	1.03×10^{6}	8.22×10^{5}	6.25×10^{5}	TACC3 (C/CS)
0	4.51×10^{2}	3.97×10^{2}	0	7.71×10^{2}	1.16×10^{4}	TAF15 (C/NU)
3.37×10^{4}	2.49×10^{4}	1.25×10^{5}	8.57×10^{4}	6.73×10^{4}	8.29×10^{4}	TBCA (CS)
5.38×10^{3}	2.04×10^{4}	2.97×10^{4}	8.55×10^{4}	7.45×10^{4}	5.62×10^{4}	TCEAL4 (NU)
1.42×10^{5}	3.24×10^{5}	8.76×10^{5}	1.45×10^{6}	1.53×10^{6}	1.68×10^{6}	THOP1 (C)
7.45×10^{5}	8.97×10^{5}	1.48×10^{6}	2.25×10^{6}	1.75×10^{6}	1.75×10^{6}	TIPRL (C)
2.82×10^{4}	3.88×10^{3}	8.72×10^{4}	2.87×10^{5}	2.04×10^{5}	1.44×10^{5}	TK1 (C)
2.17×10^{2}	1.58×10^{3}	4.82×10^{2}	2.39×10^{3}	1.33×10^{3}	1.47×10^{3}	TMA7 (???)
2.89×10^{4}	8.09×10^{4}	1.67×10^{5}	2.03×10^{5}	2.06×10^{5}	2.23×10^{5}	TPM1 (CS)
1.86×10^{6}	1.21×10^{7}	3.98×10^{7}	4.74×10^{7}	4.02×10^{7}	3.52×10^{7}	TPM3 (CS)
4.78×10^{5}	1.93×10^{6}	7.47×10^{6}	1.13×10^{7}	1.13×10^{7}	1.03×10^{7}	TPM4 (CS)
1.41×10^{5}	2.59×10^{5}	8.68×10^{5}	1.29×10^{6}	1.15×10^{6}	1.14×10^{6}	TSN (C/NU)
5.68×10^{4}	7.58×10^{4}	1.35×10^{5}	2.48×10^{5}	2.31×10^{5}	1.96×10^{5}	TSNAX (NU/GA/C)
4.22×10^{2}	2.48×10^{3}	2.86×10^{4}	1.99×10^{4}	4.24×10^{4}	3.00×10^{4}	TSR2 (???)
6.10×10^{3}	1.68×10^{4}	8.03×10^{4}	1.66×10^{5}	1.44×10^{5}	1.27×10^{5}	TTC1 (C/PO)
1.01×10^{3}	7.33×10^{3}	4.51×10^{3}	3.23×10^{3}	7.92×10^{3}	9.62×10^{4}	TTR (C/EC)
1.96×10^{4}	4.70×10^{3}	1.43×10^{5}	9.33×10^{3}	1.56×10^{4}	1.14×10^{4}	TUBA4A (CS)
7.31×10^{4}	1.45×10^{5}	3.20×10^{5}	5.67×10^{5}	4.63×10^{5}	4.11×10^{5}	TWF1 (C/CS)
1.41×10^{5}	2.12×10^{5}	3.87×10^{5}	7.07×10^{5}	6.33×10^{5}	5.10×10^{5}	TWF2 (C/CS)
0	0	0	1.31×10^{3}	1.52×10^{3}	1.35×10^{3}	TXLNA (C/EC)
3.96×10^{6}	2.83×10^{7}	1.15×10^{8}	1.41×10^{8}	1.32×10^{8}	1.21×10^{8}	TXN (C/NU/EC)

Table S6
(page 7)

external-H_2O_2 / c-YAP1C / 10 min

raw abundance: 0, 2×10^{6}, 4×10^{6}, 6×10^{6}, 8×10^{6}, 1×10^{7}

	0	10	30	100	300	1000
TXNDC17 (C)	8.04×10^{4}	7.19×10^{5}	1.69×10^{6}	2.98×10^{6}	2.31×10^{6}	1.39×10^{6}
TXNDC5 (ER)	5.14×10^{5}	9.04×10^{5}	8.68×10^{5}	1.37×10^{6}	1.24×10^{6}	1.36×10^{6}
TXNL1 (C/NU)	4.91×10^{4}	3.88×10^{4}	8.70×10^{4}	1.32×10^{5}	1.38×10^{5}	1.29×10^{5}
UBAP2 (C/NU)	3.66×10^{2}	3.49×10^{3}	1.02×10^{3}	4.48×10^{3}	4.77×10^{3}	3.27×10^{3}
UBE2A (C/NU)	1.46×10^{5}	2.68×10^{5}	2.14×10^{6}	3.92×10^{6}	3.04×10^{6}	4.32×10^{6}
UBE2B (NU/PM)	1.18×10^{5}	1.90×10^{5}	1.09×10^{6}	2.12×10^{6}	2.34×10^{6}	2.31×10^{6}
UBE2H (C/NU)	2.27×10^{4}	1.54×10^{4}	2.38×10^{4}	5.81×10^{4}	9.52×10^{4}	1.70×10^{5}
UBE2I (C/NU)	8.78×10^{4}	1.24×10^{5}	1.50×10^{5}	5.30×10^{5}	8.72×10^{5}	2.26×10^{6}
UBE2K (C)	5.65×10^{5}	7.10×10^{5}	1.04×10^{6}	2.09×10^{6}	2.37×10^{6}	2.86×10^{6}
UBE2L3 (C/NU)	2.00×10^{6}	1.38×10^{6}	1.46×10^{7}	3.05×10^{7}	2.89×10^{7}	3.40×10^{7}
UBE2N (C/NU)	9.27×10^{5}	1.08×10^{6}	1.53×10^{6}	3.17×10^{6}	4.74×10^{6}	7.37×10^{6}
UBFD1 (???)	6.39×10^{4}	6.67×10^{4}	2.12×10^{5}	5.02×10^{5}	3.82×10^{5}	4.17×10^{5}
UQCRH (MT)	7.77×10^{3}	6.43×10^{3}	2.02×10^{4}	1.26×10^{4}	7.44×10^{3}	1.07×10^{4}
VBP1 (C/NU)	3.99×10^{5}	6.05×10^{5}	2.04×10^{6}	3.20×10^{6}	2.68×10^{6}	3.12×10^{6}
VPS26B (C/ES)	1.82×10^{3}	2.05×10^{3}	9.29×10^{3}	1.26×10^{4}	1.22×10^{4}	7.15×10^{3}
WBP2 (C/NU)	2.13×10^{5}	6.29×10^{5}	2.89×10^{6}	5.09×10^{6}	3.80×10^{6}	3.26×10^{6}
WDR44 (C/GA/ES)	1.65×10^{5}	1.17×10^{5}	3.58×10^{5}	5.70×10^{5}	5.40×10^{5}	6.77×10^{5}
WDR5 (NU)	3.67×10^{5}	4.54×10^{5}	8.92×10^{5}	1.42×10^{6}	1.36×10^{6}	1.04×10^{6}
WDR77 (C/NU)	2.62×10^{5}	4.96×10^{5}	1.17×10^{6}	2.11×10^{6}	1.84×10^{6}	1.88×10^{6}
XRCC4 (NU)	6.91×10^{3}	1.21×10^{4}	7.03×10^{4}	1.38×10^{5}	1.26×10^{5}	1.31×10^{5}
YWHAE (C/NU)	6.87×10^{6}	7.82×10^{6}	1.39×10^{7}	1.77×10^{7}	1.65×10^{7}	1.62×10^{7}
YWHAG (C)	6.56×10^{5}	9.69×10^{5}	1.31×10^{6}	1.73×10^{6}	1.52×10^{6}	1.65×10^{6}
YWHAQ (C)	1.93×10^{6}	3.46×10^{6}	9.81×10^{6}	1.24×10^{7}	1.11×10^{7}	1.17×10^{7}
YWHAZ (C)	3.62×10^{6}	5.09×10^{6}	7.54×10^{6}	9.64×10^{6}	8.90×10^{6}	8.76×10^{6}
ZFAND5 (C)	0	0	0	6.94×10^{2}	0	0
ZFYVE16 (C/ES)	0	3.07×10^{2}	3.45×10^{3}	1.02×10^{4}	8.46×10^{3}	5.35×10^{3}

TABLE S7. Heat map summarizing the relative abundances of proteins trapped by c-IBD-SBP-YAP1C in response to L- or D-Ala treatment of chased versus non-chased c-DD-DAO-expressing Flp-In T-REx 293 cells. DTT eluates of the experiment shown in Fig. S11 were processed for LC-MS/MS analysis. After validation, proteins enriched 2.5-fold or more in at least one of the H_2O_2-treated conditions were retrieved, named according to their UniProtKB gene name, and sorted in alphabetical order. The primary subcellular localizations of the protein (according to the same database) are indicated between brackets. The values of the no chase (NC) conditions represent the raw protein abundances. Given that DOX significantly reduces the proliferation rate of HEK-293 cells [59], the raw abundances in the chase (C) conditions were first corrected for DOX-induced protein level differences observed as differences in total peptide abundance between the L-Ala-treated no chase and chase conditions. Protein abundances are color-coded (with white and blue being low and high, respectively). ???, no specified localization; C, cytosol; CS, cytoskeleton; EC, extracellular; ER, endoplasmic reticulum; GA, Golgi apparatus; LY, lysosome; MT, mitochondria; NU, nucleus; PM, plasma membrane.

Table S7 (page 1)

c-DD-DAO (10 min): no chase (NC) vs. chase (C)

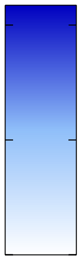

NC (L-Ala)	NC (D-Ala)	C (L-Ala)	C (D-Ala)	
1.59×10^{5}	4.55×10^{5}	1.95×10^{5}	1.74×10^{5}	ACTC1 (CS)
1.21×10^{6}	3.55×10^{6}	1.18×10^{6}	1.06×10^{6}	ACTG1 (CS)
1.07×10^{4}	6.64×10^{4}	1.62×10^{4}	2.14×10^{4}	ACTN4 (C/NU/CS)
1.54×10^{5}	5.78×10^{5}	1.79×10^{5}	1.84×10^{5}	AHCY (C)
3.69×10^{5}	9.41×10^{5}	3.71×10^{5}	4.84×10^{5}	AHSA1 (C/ER)
6.72×10^{5}	2.98×10^{6}	5.38×10^{5}	1.09×10^{6}	AIFM1 (C/NU/MT)
2.85×10^{3}	9.44×10^{3}	4.23×10^{3}	3.11×10^{3}	AKR1B1 (C)
6.92×10^{3}	2.25×10^{4}	5.53×10^{3}	6.86×10^{3}	ALDH9A1 (C)
2.36×10^{4}	7.38×10^{4}	2.57×10^{4}	1.79×10^{4}	ALDOC (C/CS/EC)
5.93×10^{5}	2.55×10^{6}	3.14×10^{5}	4.16×10^{5}	ANXA2 (PM/EC)
3.57×10^{5}	1.07×10^{6}	4.07×10^{5}	4.27×10^{5}	ANXA5 (C/EC)
8.39×10^{4}	8.08×10^{4}	1.34×10^{4}	4.57×10^{4}	ANXA6 (C)
2.24×10^{5}	5.79×10^{5}	2.11×10^{5}	2.01×10^{5}	ARF3 (GA/C)
4.83×10^{3}	1.95×10^{4}	7.01×10^{3}	5.91×10^{3}	ARF4 (GA/PM)
1.76×10^{6}	5.35×10^{6}	1.94×10^{6}	1.97×10^{6}	ARHGDIA (C)
2.12×10^{2}	2.36×10^{4}	0	0	ASF1A (NU)
3.71×10^{3}	2.22×10^{4}	3.20×10^{3}	7.19×10^{3}	ASPRV1 (C/NU)
8.94×10^{4}	2.96×10^{5}	1.07×10^{5}	9.13×10^{4}	ATP1A1 (PM)
1.69×10^{4}	5.81×10^{4}	1.61×10^{4}	1.56×10^{4}	ATP5F1C (MT)
3.34×10^{5}	8.78×10^{5}	2.41×10^{5}	2.89×10^{5}	ATP5F1D (MT)
2.36×10^{4}	6.38×10^{4}	1.80×10^{4}	1.81×10^{4}	ATP5ME (MT)
1.33×10^{3}	1.19×10^{4}	1.73×10^{3}	1.61×10^{3}	BCAT1 (C)
1.24×10^{4}	4.52×10^{4}	1.30×10^{4}	1.21×10^{4}	BLVRB (C)
3.97×10^{4}	5.51×10^{5}	2.90×10^{4}	3.34×10^{4}	BOLA2B (C/NU)
0	4.96×10^{4}	2.85×10^{2}	1.89×10^{2}	C11orf58 (???)
5.62×10^{2}	6.14×10^{3}	8.18×10^{2}	1.46×10^{3}	CACYBP (C/NU)
7.33×10^{4}	1.91×10^{5}	5.72×10^{4}	4.45×10^{4}	CAPZA1 (CS)
9.13×10^{4}	3.30×10^{5}	1.41×10^{5}	7.56×10^{4}	CAPZB (CS)
6.43×10^{3}	7.07×10^{4}	1.18×10^{4}	1.72×10^{4}	CARHSP1 (C)
3.41×10^{3}	2.47×10^{4}	5.06×10^{3}	3.67×10^{3}	CASP3 (C)
8.19×10^{3}	1.72×10^{5}	3.08×10^{3}	3.79×10^{3}	CAST(C/ER)
2.39×10^{4}	7.34×10^{4}	2.67×10^{4}	3.35×10^{4}	CBX1 (NU)
3.71×10^{5}	1.25×10^{6}	3.58×10^{5}	5.19×10^{5}	CBX3 (NU)
2.06×10^{3}	1.60×10^{5}	4.88×10^{2}	2.69×10^{2}	CDK4 (C/NU)
1.25×10^{4}	2.03×10^{5}	1.13×10^{4}	1.35×10^{4}	CDKN2A (C/NU)
1.64×10^{6}	5.15×10^{6}	1.43×10^{6}	1.48×10^{6}	CFL1 (C/NU/CS)
5.42×10^{3}	1.86×10^{4}	9.46×10^{3}	1.12×10^{4}	CHORDC1 (???)
2.45×10^{3}	2.63×10^{5}	2.14×10^{3}	3.83×10^{3}	CIAPIN1 (C/NU/MT)
2.17×10^{6}	6.30×10^{6}	2.34×10^{6}	2.10×10^{6}	CKB (C)
4.92×10^{4}	1.23×10^{5}	4.50×10^{4}	4.82×10^{4}	CNN3 (C/CS)
5.71×10^{4}	1.64×10^{5}	7.84×10^{3}	1.99×10^{3}	COX17 (C/MT)
1.06×10^{5}	5.26×10^{5}	8.04×10^{4}	1.49×10^{5}	COX5B (MT)
1.24×10^{4}	3.46×10^{4}	1.31×10^{4}	1.14×10^{4}	CPD (PM)
1.43×10^{4}	1.59×10^{5}	1.42×10^{4}	1.44×10^{4}	CPOX (MT)
3.42×10^{3}	3.52×10^{5}	4.42×10^{2}	2.02×10^{3}	CRKL (C/NU/PM)
6.91×10^{4}	1.16×10^{6}	2.46×10^{4}	1.90×10^{4}	CSTB (C/NU)
2.69×10^{5}	9.95×10^{5}	1.92×10^{5}	2.94×10^{5}	CTSB (LY/EC)
1.59×10^{5}	4.00×10^{5}	1.14×10^{5}	2.00×10^{5}	CTSD (LY/EC)
6.01×10^{4}	4.13×10^{5}	4.55×10^{4}	1.07×10^{5}	CTTN (CS/PM)
4.89×10^{3}	2.46×10^{4}	5.97×10^{3}	5.51×10^{3}	CYB5R3 (C/MT/ER)

Table S7
(page 2)

c-DD-DAO (10 min): no chase (NC) vs. chase (C)

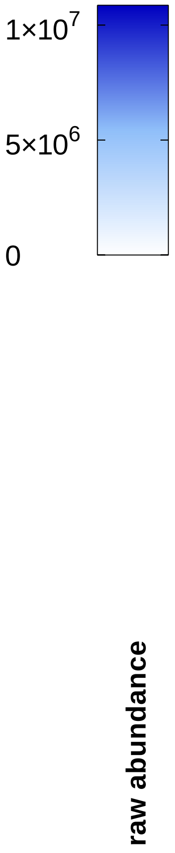

NC (L-Ala)	NC (D-Ala)	C (L-Ala)	C (D-Ala)	
3.17×10^{2}	6.68×10^{4}	8.70×10^{1}	1.92×10^{2}	DCTPP1 (C/NU/MT)
4.94×10^{3}	3.69×10^{4}	1.18×10^{4}	8.15×10^{3}	DDB1 (C/NU)
8.50×10^{4}	2.64×10^{5}	6.11×10^{4}	5.76×10^{4}	DDOST (ER)
1.23×10^{5}	3.61×10^{5}	1.36×10^{5}	1.20×10^{5}	DDX39B (C/NU)
2.29×10^{5}	1.29×10^{6}	2.33×10^{5}	4.42×10^{5}	DNAJB11 (ER)
9.22×10^{4}	4.17×10^{5}	8.42×10^{4}	1.25×10^{5}	DPYSL2 (C/CS/PM)
3.26×10^{3}	7.96×10^{4}	1.27×10^{3}	6.88×10^{2}	DPYSL3 (C)
3.72×10^{4}	1.50×10^{5}	3.09×10^{4}	2.90×10^{4}	DSTN (C/CS/EC)
8.03×10^{4}	3.43×10^{5}	6.02×10^{4}	8.85×10^{4}	DUT (NU/MT)
0	1.36×10^{4}	1.16×10^{3}	2.67×10^{2}	DYNLL1 (NU/CS/MT)
4.83×10^{5}	1.47×10^{6}	5.72×10^{5}	5.36×10^{5}	EEF1A1 (C/NU/PM)
1.19×10^{5}	3.67×10^{5}	7.07×10^{4}	1.09×10^{5}	EEF1B2 (C)
8.46×10^{3}	4.06×10^{4}	1.22×10^{4}	1.02×10^{4}	EEF1D (NU)
2.50×10^{3}	1.20×10^{4}	3.19×10^{3}	2.62×10^{3}	EEF1G (C/NU/EC)
5.16×10^{3}	3.49×10^{4}	1.08×10^{4}	9.42×10^{3}	EIF4A2 (C)
2.76×10^{4}	1.20×10^{5}	2.24×10^{4}	2.46×10^{4}	EIF5A (C/NU/ER)
7.79×10^{3}	8.06×10^{4}	1.84×10^{4}	2.39×10^{4}	EIF6 (C/NU)
2.68×10^{5}	8.61×10^{5}	2.39×10^{5}	2.24×10^{5}	ELOC (NU)
9.40×10^{5}	2.62×10^{6}	8.91×10^{5}	8.79×10^{5}	ENO1 (C/NU)
5.04×10^{2}	4.83×10^{3}	2.20×10^{2}	1.23×10^{3}	ENO3 (C)
1.40×10^{3}	1.64×10^{4}	3.53×10^{3}	2.93×10^{3}	ENSG00000276612 (MT)
2.10×10^{6}	6.81×10^{6}	1.60×10^{6}	1.88×10^{6}	ERP44 (ER)
1.64×10^{5}	5.78×10^{5}	1.47×10^{5}	1.70×10^{5}	FLG (C/PM)
2.94×10^{4}	1.14×10^{5}	2.67×10^{4}	2.29×10^{4}	FSCN1 (C/CS)
3.30×10^{4}	8.40×10^{4}	3.44×10^{4}	3.84×10^{4}	GANAB (GA/ER)
1.04×10^{6}	2.79×10^{6}	1.22×10^{6}	1.23×10^{6}	GAPDH (C/NU/CS)
3.64×10^{2}	4.55×10^{3}	6.91×10^{2}	6.28×10^{2}	GCLC (C)
4.45×10^{2}	3.12×10^{4}	2.59×10^{2}	1.63×10^{2}	GCLM (C)
5.90×10^{4}	1.80×10^{5}	5.49×10^{4}	4.69×10^{4}	GDI1 (C/GA)
1.56×10^{2}	4.75×10^{2}	4.47×10^{2}	2.83×10^{3}	GFAP (C)
2.35×10^{5}	9.74×10^{5}	2.55×10^{5}	3.67×10^{5}	GLO1 (C/NU/PM/EC)
2.88×10^{4}	1.16×10^{5}	3.35×10^{3}	5.00×10^{3}	GLOD4 (MT)
5.17×10^{3}	1.86×10^{5}	5.69×10^{3}	4.32×10^{3}	GPHN (C/PM)
1.54×10^{4}	2.03×10^{5}	1.46×10^{4}	2.13×10^{4}	GPX4 (C/MT)
5.70×10^{4}	4.64×10^{6}	4.09×10^{4}	5.50×10^{4}	GSR (C/MT)
4.34×10^{3}	3.34×10^{4}	2.76×10^{3}	4.62×10^{3}	GSTO1 (C)
3.49×10^{4}	2.68×10^{5}	4.07×10^{4}	4.64×10^{4}	GSTP1 (C/NU/MT)
8.77×10^{3}	5.44×10^{4}	9.29×10^{3}	5.87×10^{3}	H2AC6 (NU)
5.73×10^{3}	1.89×10^{4}	5.08×10^{3}	6.02×10^{3}	HNRNPA2B1 (C/NU/EC)
4.07×10^{4}	1.73×10^{5}	3.80×10^{4}	3.93×10^{4}	HNRNPDL (C/NU)
1.96×10^{4}	1.88×10^{6}	1.75×10^{4}	1.43×10^{4}	HPRT1 (C)
2.32×10^{4}	5.76×10^{5}	2.81×10^{4}	4.03×10^{4}	HSD17B10 (MT)
4.67×10^{3}	1.33×10^{4}	3.96×10^{3}	3.08×10^{3}	HSD17B4 (PO)
1.06×10^{6}	2.94×10^{6}	1.18×10^{6}	1.05×10^{6}	HSP90AA1 (C/NU/MT/PM)
1.00×10^{6}	3.45×10^{6}	1.45×10^{6}	1.32×10^{6}	HSP90AB1 (C/NU/PM/EC)
1.58×10^{6}	4.70×10^{6}	1.61×10^{6}	1.61×10^{6}	HSP90B1 (ER)
2.86×10^{6}	7.90×10^{6}	3.25×10^{6}	3.10×10^{6}	HSPA1B (C/CS)
2.81×10^{5}	7.11×10^{5}	3.26×10^{5}	2.95×10^{5}	HSPA4 (C)
9.59×10^{3}	5.35×10^{4}	1.80×10^{4}	1.61×10^{4}	HSPA4L (C/NU)
5.46×10^{5}	1.72×10^{6}	5.47×10^{5}	7.77×10^{5}	HSPA8 (C/PM/NU)

Table S7 (page 3)

c-DD-DAO (10 min): no chase (NC) vs. chase (C)

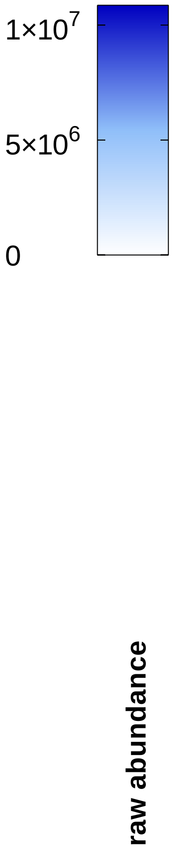

NC (L-Ala)	NC (D-Ala)	C (L-Ala)	C (D-Ala)	
6.60×10^{4}	4.37×10^{5}	4.96×10^{4}	6.52×10^{4}	HSPB1 (C/NU/CS)
3.69×10^{4}	1.37×10^{5}	4.66×10^{4}	3.83×10^{4}	HSPH1 (C)
6.88×10^{3}	1.78×10^{4}	7.04×10^{3}	5.69×10^{3}	IMPA1 (C)
2.83×10^{4}	9.14×10^{4}	2.76×10^{4}	3.63×10^{4}	ISOC1 (C/PO)
4.72×10^{3}	3.19×10^{5}	6.06×10^{3}	5.33×10^{3}	ISYNA1 (C)
1.52×10^{5}	4.33×10^{5}	1.56×10^{5}	1.44×10^{5}	KCTD12 (PM)
3.62×10^{3}	6.10×10^{4}	5.41×10^{2}	1.64×10^{3}	LCP1 (PM/CS)
1.66×10^{5}	4.39×10^{5}	1.44×10^{5}	1.38×10^{5}	LDHA (C)
9.55×10^{4}	5.90×10^{5}	7.07×10^{4}	7.58×10^{4}	LGALS7 (C/NU/EC)
1.16×10^{4}	6.28×10^{4}	4.54×10^{3}	6.25×10^{3}	LMNA (NU)
1.12×10^{5}	2.97×10^{5}	1.36×10^{5}	1.05×10^{5}	LTA4H (C)
3.63×10^{3}	1.74×10^{4}	6.22×10^{3}	5.93×10^{3}	LUC7L2(NU)
1.67×10^{4}	4.81×10^{4}	1.62×10^{4}	1.01×10^{4}	LXN (C)
3.10×10^{3}	1.59×10^{4}	4.26×10^{3}	5.54×10^{3}	MAD2L1(C/CS/NU)
1.12×10^{4}	2.49×10^{5}	7.79×10^{3}	8.08×10^{3}	MARCKS (CS/PM)
6.62×10^{2}	2.50×10^{4}	2.01×10^{2}	7.36×10^{2}	MARCKSL1(CS/PM)
1.07×10^{5}	3.68×10^{5}	1.15×10^{5}	9.17×10^{4}	MAT2A (C)
4.65×10^{4}	3.99×10^{5}	5.80×10^{4}	4.66×10^{4}	MIF (C/EC)
3.34×10^{4}	1.13×10^{5}	2.84×10^{4}	2.55×10^{4}	MYG1 (NU/MT)
1.11×10^{4}	4.49×10^{4}	4.53×10^{3}	5.44×10^{3}	MYH9 (CS)
1.12×10^{4}	3.36×10^{4}	7.18×10^{3}	6.90×10^{3}	MYL6 (C/CS/EC)
1.01×10^{5}	3.68×10^{5}	1.02×10^{5}	9.64×10^{4}	NASP (C/NU)
3.49×10^{5}	1.57×10^{6}	3.41×10^{5}	4.89×10^{5}	NME1 (C/NU)
1.71×10^{5}	5.42×10^{5}	1.49×10^{5}	1.08×10^{5}	NPEPPS (C/NU)
1.94×10^{5}	8.66×10^{5}	2.24×10^{5}	3.04×10^{5}	NPM1 (NU)
6.04×10^{3}	3.25×10^{5}	3.97×10^{3}	5.33×10^{3}	NUDC (CS/NU)
4.96×10^{4}	1.64×10^{5}	5.27×10^{4}	6.50×10^{4}	NUDCD2 (CS)
2.38×10^{4}	7.30×10^{4}	1.93×10^{4}	2.08×10^{4}	NUDT5 (NU)
2.31×10^{6}	5.88×10^{6}	1.74×10^{6}	1.91×10^{6}	P4HB (PM/ER)
7.36×10^{3}	4.47×10^{4}	1.52×10^{4}	1.26×10^{4}	PAFAH1B3 (C)
6.57×10^{3}	8.83×10^{4}	4.45×10^{3}	4.87×10^{3}	PAICS (C/EC)
4.66×10^{5}	1.28×10^{6}	4.15×10^{5}	4.69×10^{5}	PARK7 (C/ER/NU/PM/MT)
1.28×10^{5}	3.73×10^{5}	1.48×10^{5}	1.64×10^{5}	PBDC1 (???)
7.38×10^{4}	2.09×10^{5}	7.73×10^{4}	7.87×10^{4}	PCCA (MT)
1.35×10^{4}	5.63×10^{5}	3.35×10^{4}	5.28×10^{4}	PCMT1 (C)
6.55×10^{5}	2.98×10^{6}	7.11×10^{5}	6.59×10^{5}	PDIA3 (ER)
3.65×10^{5}	1.34×10^{6}	3.77×10^{5}	4.18×10^{5}	PDIA4 (ER)
8.86×10^{4}	3.51×10^{5}	8.26×10^{4}	9.92×10^{4}	PDIA6 (ER/PM)
2.58×10^{5}	9.31×10^{5}	2.83×10^{5}	2.68×10^{5}	PEBP1 (C)
8.14×10^{2}	6.08×10^{4}	6.09×10^{1}	7.26×10^{2}	PFDN2 (C/NU/MT)
0	1.08×10^{5}	0	0	PFDN4 (C/NU/MT)
5.52×10^{4}	1.90×10^{5}	3.09×10^{4}	2.94×10^{4}	PFN1 (CS)
1.67×10^{4}	4.39×10^{4}	1.62×10^{4}	1.40×10^{4}	PGD (C)
7.00×10^{4}	2.43×10^{5}	8.73×10^{4}	7.98×10^{4}	PGK1 (C)
1.55×10^{5}	6.93×10^{5}	1.18×10^{5}	1.78×10^{5}	PHPT1 (C)
1.79×10^{3}	4.49×10^{5}	1.46×10^{3}	1.24×10^{3}	PITHD1 (C)
2.53×10^{4}	9.34×10^{4}	2.35×10^{4}	3.06×10^{4}	PKM (C/NU)
2.01×10^{5}	7.02×10^{5}	1.03×10^{5}	1.92×10^{5}	PKP1 (NU)
1.26×10^{4}	5.34×10^{4}	1.99×10^{4}	2.05×10^{4}	PLS1 (C)
2.44×10^{4}	2.23×10^{5}	2.35×10^{4}	2.17×10^{4}	PLS3 (C)

Table S7
(page 4)

c-DD-DAO (10 min): no chase (NC) vs. chase (C)

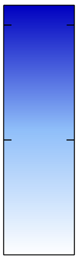

NC (L-Ala)	NC (D-Ala)	C (L-Ala)	C (D-Ala)	
5.43×10^{4}	1.60×10^{5}	2.49×10^{4}	4.28×10^{4}	POF1B (C/CS)
3.33×10^{5}	1.13×10^{6}	4.13×10^{5}	3.35×10^{5}	PPA1 (C)
2.64×10^{6}	7.95×10^{6}	2.53×10^{6}	2.16×10^{6}	PPIA (C/NU/EC)
4.15×10^{3}	3.18×10^{5}	2.09×10^{3}	7.69×10^{3}	PPP2R2A (C/NU)
2.86×10^{6}	4.05×10^{7}	2.43×10^{6}	1.64×10^{6}	PRDX1 (C)
7.39×10^{5}	8.36×10^{6}	6.26×10^{5}	5.39×10^{5}	PRDX2 (C)
5.65×10^{5}	1.12×10^{7}	3.65×10^{5}	4.46×10^{5}	PRDX3 (MT/C/EE)
4.41×10^{5}	3.47×10^{6}	3.78×10^{5}	4.25×10^{5}	PRDX4 (C/ER)
1.57×10^{3}	5.49×10^{4}	2.03×10^{3}	3.42×10^{3}	PRDX5 (C/NU/MT/PO)
8.82×10^{4}	1.31×10^{6}	5.20×10^{4}	1.07×10^{5}	PRDX6 (C/LY)
6.03×10^{3}	1.21×10^{5}	3.23×10^{3}	3.57×10^{3}	PRMT5 (C/NU/GA)
1.56×10^{4}	4.68×10^{4}	1.52×10^{4}	1.50×10^{4}	PSMA7 (C/NU)
5.57×10^{4}	1.49×10^{5}	6.94×10^{4}	6.73×10^{4}	PSMD1 (C/NU/LY/EC)
4.57×10^{4}	1.76×10^{5}	6.61×10^{4}	6.79×10^{4}	PSMD4 (C/NU)
5.52×10^{3}	2.43×10^{4}	2.78×10^{3}	3.54×10^{3}	RAB11A (PM/ES)
3.27×10^{4}	8.20×10^{4}	4.56×10^{4}	3.53×10^{4}	RAB1A (C/ES/GA/PM)
1.12×10^{4}	4.55×10^{4}	6.12×10^{3}	8.54×10^{3}	RAB2A (GA/ER)
3.08×10^{4}	8.20×10^{4}	3.06×10^{4}	2.88×10^{4}	RAB35 (PM/ES)
1.12×10^{4}	4.21×10^{4}	1.27×10^{4}	1.16×10^{4}	RACK1 (C/NU/PM)
7.65×10^{5}	2.69×10^{6}	6.13×10^{5}	7.16×10^{5}	RANBP1 (C/NU/CS)
1.50×10^{4}	5.44×10^{4}	1.36×10^{4}	1.36×10^{4}	RAP1A (PM/C/ES)
2.80×10^{4}	6.53×10^{5}	3.68×10^{4}	2.86×10^{4}	RBBP7 (NU)
2.71×10^{5}	7.25×10^{5}	2.31×10^{5}	2.79×10^{5}	RCN1 (ER)
1.12×10^{4}	3.43×10^{4}	8.35×10^{3}	8.14×10^{3}	RPA3 (NU)
3.61×10^{2}	2.83×10^{3}	5.88×10^{2}	2.96×10^{2}	RPL8 (C)
2.72×10^{4}	8.32×10^{4}	1.63×10^{4}	1.53×10^{4}	RPLP2 (C/EC)
4.25×10^{4}	1.42×10^{5}	4.25×10^{4}	4.52×10^{4}	RPN1 (ER)
3.36×10^{4}	1.07×10^{5}	4.09×10^{4}	3.95×10^{4}	RPN2 (ER)
7.54×10^{4}	4.89×10^{5}	7.00×10^{4}	1.48×10^{5}	RPS21 (C/ER)
8.01×10^{5}	2.71×10^{6}	6.67×10^{5}	6.79×10^{5}	RPS27A (C/NU)
6.75×10^{4}	2.19×10^{5}	6.70×10^{4}	4.51×10^{4}	RPSA (NU/C/PM)
1.90×10^{4}	6.92×10^{4}	3.03×10^{4}	2.24×10^{4}	RUVBL2 (C/NU)
6.64×10^{4}	2.27×10^{5}	2.64×10^{4}	2.43×10^{4}	S100A7 (C/EC)
1.20×10^{4}	3.02×10^{4}	8.05×10^{3}	7.61×10^{3}	SCO2 (MT)
1.57×10^{3}	1.36×10^{4}	6.29×10^{1}	4.87×10^{1}	SERPINB1 (C/ES/LY/EC)
9.10×10^{3}	2.55×10^{4}	3.78×10^{3}	4.70×10^{3}	SERPINB7 (C)
1.74×10^{4}	1.15×10^{5}	7.12×10^{3}	4.33×10^{3}	SFN (C/NU/EC)
4.98×10^{3}	9.85×10^{5}	7.27×10^{3}	1.02×10^{4}	SKP1 (C/NU)
1.62×10^{5}	6.17×10^{5}	1.62×10^{5}	2.38×10^{5}	SNRPF (C/NU)
3.26×10^{3}	2.58×10^{4}	2.91×10^{3}	5.70×10^{3}	SOD1 (C/NU/MT)
4.97×10^{3}	1.51×10^{4}	1.17×10^{4}	1.46×10^{4}	SSR1 (ER)
4.18×10^{4}	1.21×10^{5}	3.79×10^{4}	3.60×10^{4}	SSR4 (ER)
1.23×10^{4}	4.36×10^{4}	1.74×10^{4}	1.21×10^{4}	ST13 (C)
8.85×10^{3}	2.95×10^{4}	1.29×10^{4}	7.78×10^{3}	STIP1 (C/NU)
6.08×10^{4}	4.44×10^{5}	6.74×10^{4}	5.43×10^{4}	STRAP (C/NU)
9.95×10^{3}	3.20×10^{4}	1.15×10^{4}	1.16×10^{4}	SYPL1 (VE)
1.27×10^{3}	9.93×10^{3}	9.65×10^{2}	2.03×10^{2}	THOP1 (C)
4.90×10^{3}	3.42×10^{4}	7.17×10^{3}	7.78×10^{3}	TIPRL (C)
3.52×10^{3}	1.35×10^{4}	5.42×10^{3}	5.61×10^{3}	TKT (C/NU/ER/PO/EC)
1.54×10^{4}	3.95×10^{4}	1.30×10^{4}	1.14×10^{4}	TMED10 (PM/ER/GA)

Table S7
(page 5)

c-DD-DAO (10 min): no chase (NC) vs. chase (C)

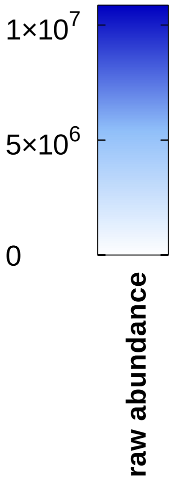

	NC (L-Ala)	NC (D-Ala)	C (L-Ala)	C (D-Ala)
TMED7	7.92×10^{4}	3.00×10^{5}	5.40×10^{4}	5.04×10^{4}
TOMM22	2.90×10^{4}	8.01×10^{4}	3.01×10^{4}	2.79×10^{4}
TOMM40	7.82×10^{3}	2.58×10^{4}	1.01×10^{4}	7.73×10^{3}
TPM3	8.77×10^{4}	2.50×10^{6}	3.44×10^{4}	5.15×10^{4}
TPM4	7.79×10^{3}	2.84×10^{5}	1.64×10^{3}	3.14×10^{3}
TRAP1	7.65×10^{4}	2.44×10^{5}	7.87×10^{4}	8.00×10^{4}
TRMT112	1.14×10^{4}	4.15×10^{4}	1.05×10^{4}	1.16×10^{4}
TUBA1C	4.63×10^{4}	1.27×10^{5}	3.18×10^{4}	2.68×10^{4}
TUBB	2.05×10^{3}	3.72×10^{4}	2.91×10^{3}	2.86×10^{3}
TXN	7.37×10^{5}	1.85×10^{7}	4.00×10^{5}	4.77×10^{5}
TXNDC17	4.75×10^{3}	8.02×10^{5}	4.34×10^{3}	9.65×10^{3}
UBA1	3.48×10^{5}	1.01×10^{6}	4.25×10^{5}	3.68×10^{5}
UBE2K	9.95×10^{3}	9.96×10^{4}	1.71×10^{4}	2.73×10^{4}
UBE2L3	0	5.92×10^{4}	7.78×10^{2}	7.87×10^{2}
UCHL1	4.42×10^{6}	1.28×10^{7}	4.82×10^{6}	4.38×10^{6}
UGGT1	6.01×10^{4}	1.97×10^{5}	4.23×10^{4}	6.95×10^{4}
UQCRFS1	1.14×10^{5}	4.84×10^{5}	9.99×10^{4}	1.63×10^{5}
VCP	6.72×10^{4}	2.79×10^{5}	7.36×10^{4}	8.16×10^{4}
VIM	1.19×10^{3}	1.19×10^{4}	2.19×10^{3}	1.30×10^{3}
WDR77	4.58×10^{3}	1.40×10^{5}	7.12×10^{3}	5.89×10^{3}
YWHAB	2.53×10^{5}	6.64×10^{5}	2.08×10^{5}	2.42×10^{5}
YWHAE	9.77×10^{5}	3.46×10^{6}	9.00×10^{5}	9.51×10^{5}
YWHAG	1.64×10^{4}	7.14×10^{4}	1.71×10^{4}	1.55×10^{4}
YWHAQ	1.93×10^{5}	1.51×10^{6}	2.45×10^{5}	2.54×10^{5}
YWHAZ	2.78×10^{5}	1.05×10^{6}	2.95×10^{5}	3.08×10^{5}
ZPR1	2.48×10^{3}	7.48×10^{3}	3.68×10^{3}	2.69×10^{3}

TABLE S8. Heat map summarizing the percentage distribution of NaCl- and DTT-eluted proteins trapped by c-IBD-SBP-YAP1C upon treatment of Flp-In T-REx 293 cells with 1 mM H_2O_2 for 10 min. The percentage distribution of proteins trapped by c-IBD-SBP-YAP1C after H_2O_2 treatment and sequentially eluted with 1 M NaCl and 10 mM DTT was calculated and presented in a heat map (with red and green intensities representing low and high percentages of elution). The UniProtKB gene name was used to name the interactors, and their primary subcellular localizations (according to the same database) are indicated between brackets. ???, no specified localization; C, cytosol; CS, cytoskeleton; EC, extracellular; ER, endoplasmic reticulum; ES, endosome; GA, Golgi apparatus; LY, lysosome; MT, mitochondria; NU, nucleus; PM, plasma membrane; PO, peroxisome.

Table S8
(page 1)

External H_2O_2 (1 mM, 10 min)

Colour scale: 100, 80, 60, 40, 20, 0 — protein abundance (percentage distribution)

	NaCl eluate	DTT eluate
AAMDC (C)	19.94	80.06
AASDHPPT (C)	4.68	95.32
ACP1 (C)	15.82	84.18
ACP6 (MT)	0.63	99.37
ACT (???)	17.54	82.46
ACTN1 (C/PM)	40.51	59.49
ACYP2 (?)	1.01	98.99
ADO (C)	3.74	96.26
AHNAK (NU)	16.98	83.02
AIF1L (CS/PM)	1.38	98.62
AK2 (MT)	4.74	95.26
AKAP12 (CS/PM)	14.67	85.33
ALDH18A1 (MT)	37.27	62.73
AMPD2 (C)	24.57	75.43
ANXA1 (C/NU/PM/EC)	25.30	74.70
ANXA2 (PM/EC)	8.25	91.75
ANXA7 (C/ER/NU/EC)	21.17	78.83
ARFGAP1 (GA/C)	0.93	99.07
ARL6IP4 (NU)	24.90	75.10
ASF1A (NU)	4.38	95.62
ASF1B (NU)	7.15	92.85
ASNS (C)	4.64	95.36
ASPRV1 (C/NU)	10.26	89.74
ASRGL1 (C)	34.14	65.86
ATOX1 (C)	0.44	99.56
BAG3 (C/NU)	7.63	92.37
BAG4 (C)	1.85	98.15
BAG5 (C/NU/MT)	2.56	97.44
BICD2 (C/NU/CS/GA)	6.26	93.74
BID (C/MT)	3.69	96.31
BLMH (C)	45.25	54.75
BOLA1 (MT)	5.48	94.52
BOLA2B (C/NU)	4.78	95.22
C11orf54 (NU)	2.04	97.96
C11orf58 (???)	5.54	94.46
C1orf174 (NU)	0	100.00
C1orf52 (NU)	2.72	97.28
CALD1 (CS)	14.08	85.92
CARHSP1 (C)	17.34	82.66
CASP3 (C)	18.89	81.11
CAST(C/ER)	4.37	95.63
CCDC50 (C)	4.15	95.85
CCDC6 (C/CS)	8.10	91.90
CCS (C)	11.59	88.41
CDK4 (C/NU)	9.21	90.79
CDK6(C/NU/CS)	40.85	59.15
CDKN2A (C/NU)	13.75	86.25
CHAC2 (C)	9.98	90.02
CHMP5 (C/ES)	5.40	94.60
CIAPIN1 (C/NU/MT)	5.60	94.40

Table S8 (page 2)

External H_2O_2 (1 mM, 10 min)

protein abundance (percentage distribution)

Protein	NaCl eluate	DTT eluate
CKMT1A (MT)	6.60	93.40
CLIC1 (C/NU/PM)	31.32	68.68
COX17 (C/MT)	0.17	99.83
CPOX (MT)	5.10	94.90
CPPED1 (C)	2.70	97.30
CRKL (C/NU/PM)	5.06	94.94
CRMP1 (C/CS)	20.52	79.48
CRYZL1 (C)	9.59	90.41
CSTB (C/NU)	1.72	98.28
CTH (C)	2.49	97.51
CTSG (C/NU/LY/PM/EC)	76.08	23.92
CTTN (CS/PM)	32.05	67.95
CWC15 (NU)	3.41	96.59
DCPS (C/NU)	2.54	97.46
DCTPP1 (C/NU/MT)	5.99	94.01
DENR (???)	2.34	97.66
DHPS (C)	3.01	96.99
DLAT (MT)	14.54	85.46
DNPEP (C)	2.44	97.56
DPYSL3 (C)	18.38	81.62
DPYSL5 (C)	11.58	88.42
DSC1 (PM)	18.23	81.77
DSC3 (PM)	15.45	84.55
DSG1 (PM)	19.34	80.66
DSP (CS/PM)	14.77	85.23
DUSP3 (NU)	19.04	80.96
DYNLL1 (NU/CS/MT)	6.02	93.98
ECI1 (MT)	0.89	99.11
EFHD1 (MT)	13.26	86.74
EFHD2(PM)	2.39	97.61
EIF1AD (NU)	15.07	84.93
EIF3B (C)	40.98	59.02
EIF4B (C)	4.66	95.34
EIF4EBP1 (C/NU)	0.06	99.94
EIF6 (C/NU)	22.92	77.08
EMD (NU)	20.66	79.34
EPPK1 (CS)	2.84	97.16
EPS15L1 (NU/PM)	0.70	99.30
ERICH5 (???)	6.78	93.22
EVPL (CS)	8.10	91.90
FAH (C/EC)	0.85	99.15
FLG (C/PM)	10.08	89.92
FLG2 (C)	46.36	53.64
FLYWCH2 (???)	6.09	93.91
FN3KRP (C)	14.60	85.40
FNTA (C/PM/CS)	5.23	94.77
FNTB (C/CS)	1.66	98.34
FTH1 (C/NU/LY/EC)	10.90	89.10
GCLC (C)	4.80	95.20
GCLM (C)	6.39	93.61

Table S8
(page 3)

External H_2O_2 (1 mM, 10 min)

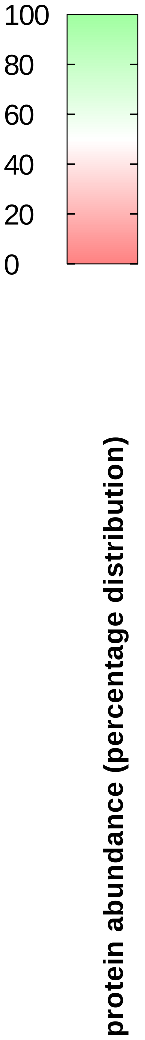

NaCl eluate	DTT eluate	
12.15	87.85	GEMIN6 (C/NU)
2.04	97.96	GFER (C/MT/EC)
15.13	84.87	GLDC (MT)
8.69	91.31	GLOD4 (MT)
28.42	71.58	GLRX3 (C)
18.01	81.99	GLRX5 (MT)
77.67	22.33	GLUD2 (MT)
7.51	92.49	GPHN (C/PM)
30.09	69.91	GPX1 (C)
7.30	92.70	GRB2 (C/NU/EE/GA)
5.08	94.92	GRIPAP1 (ES)
2.96	97.04	GSN (CS/EC)
2.75	97.25	GSR (C/MT)
24.49	75.51	GSTO1 (C)
1.29	98.71	HDGF (NU)
10.39	89.61	HEXIM1 (C/NU)
7.26	92.74	HIRIP3 (NU)
5.80	94.20	HPRT1 (C)
7.47	92.53	HSD17B10 (MT)
1.38	98.62	HSD17B8 (MT)
23.19	76.81	HSPA4L (C/NU)
7.90	92.10	HSPB1 (C/NU/CS)
1.40	98.60	IAH1 (???)
1.95	98.05	IBA57 (MT)
18.40	81.60	IMPDH2 (C/NU)
14.05	85.95	ISCA2 (MT)
3.25	96.75	ISCU (C/NU/MT)
5.50	94.50	ISYNA1 (C)
7.58	92.42	IVL (C)
72.75	27.25	JMJD6 (C/NU)
2.89	97.11	JPT2 (C/NU)
14.71	85.29	JUP (CS/PM)
4.23	95.77	KCTD5 (C/NU)
64.88	35.12	KHDRBS1 (C/NU/PM)
8.08	91.92	KHSRP (C/NU)
19.01	80.99	KIF3A (CS)
0.36	99.64	LBH (C/NU)
10.97	89.03	LCP1 (PM/CS)
23.01	76.99	LGALS7 (C/NU/EC)
34.33	65.67	LMNA (NU)
7.96	92.04	LRRFIP1 (C/NU)
51.89	48.11	LSM2 (NU)
13.02	86.98	LYRM7 (MT)
8.83	91.17	MAP4 (CS)
31.02	68.98	MAPK14 (C/NU)
11.65	88.35	MARCKS (CS/PM)
1.03	98.97	MARCKSL1(CS/PM)
9.50	90.50	MCMBP (NU)
11.37	88.63	MDH1 (C)
9.62	90.38	METTL26 (???)

Table S8 (page 4)

External H_2O_2 (1 mM, 10 min)

Color scale: protein abundance (percentage distribution), 0–100 (0, 20, 40, 60, 80, 100)

Protein	NaCl eluate	DTT eluate
MINDY3 (NU)	27.76	72.24
MMAB (MT)	15.54	84.46
MOCS2 (C)	5.15	94.85
MPP1 (PM)	9.97	90.03
MSRB3 (MT/ER)	6.29	93.71
MTAP (C/NU)	4.42	95.58
NACA (C/NU)	30.47	69.53
NAGK (C/EC)	4.46	95.54
NDUFAB1 (MT)	24.98	75.02
NFU1 (C/MT)	10.13	89.87
NT5DC2 (???)	5.75	94.25
NUDC (CS/NU)	3.63	96.37
OGFR (C/NU)	4.78	95.22
OPTN (C/GA/RE)	9.61	90.39
OTUB1 (C)	18.88	81.12
PABPC4 (C)	51.19	48.81
PAICS (C/EC)	14.71	85.29
PARVA (CS/PM)	35.04	64.96
PAWR (C/NU)	2.85	97.15
PBK (NU)	8.35	91.65
PCBP1 (C/NU)	20.71	79.29
PCBP2 (C/NU)	11.10	88.90
PCMT1 (C)	6.64	93.36
PDCD6IP (C/CS)	38.25	61.75
PDHB (MT)	6.52	93.48
PFDN2 (C/NU/MT)	10.89	89.11
PFDN4 (C/NU/MT)	7.73	92.27
PFDN5 (C/NU)	2.89	97.11
PFN2 (CS)	11.60	88.40
PHAX (C/NU)	9.19	90.81
PIN1 (C/NU)	5.65	94.35
PITHD1 (C)	2.89	97.11
PITRM1 (MT)	21.04	78.96
PKM (C/NU)	37.57	62.43
PKP1 (NU)	13.01	86.99
PLS3 (C)	23.34	76.66
PM20D2 (NU)	14.16	85.84
PNPO (C)	4.91	95.09
POF1B (C/CS)	14.61	85.39
POLDIP2 (NU/MT)	23.16	76.84
PPAT (C)	21.46	78.54
PPCDC (C)	4.18	95.82
PPL (C/CS/PM)	0.87	99.13
PPP1CA (C/NU)	91.06	8.94
PPP1R11 (C/NU)	2.97	97.03
PPP1R2 (???)	0	100.00
PPP1R7 (NU)	26.03	73.97
PPP2R2A (C/NU)	1.76	98.24
PPP2R2D (C)	1.76	98.24
PPP5C (C/NU/PM)	38.92	61.08

Table S8
(page 5)

External H_2O_2 (1 mM, 10 min)

Color scale: protein abundance (percentage distribution), 0–100 (0, 20, 40, 60, 80, 100)

Protein	NaCl eluate	DTT eluate
PRDX1 (C)	4.06	95.94
PRDX2 (C)	9.17	90.83
PRDX3 (MT/C/EE)	9.94	90.06
PRDX4 (C/ER)	2.23	97.77
PRDX5 (C/NU/MT/PO)	8.32	91.68
PRDX6 (C/LY)	4.91	95.09
PREP (C)	31.79	68.21
PRMT5 (C/NU/GA)	16.12	83.88
PSMA4 (C/NU)	49.28	50.72
PSMA6 (C/NU)	60.86	39.14
PSMB2 (C/NU)	50.86	49.14
PSMD9 (C/NU)	6.77	93.23
PSME3 (C/NU)	14.05	85.95
PSME3IP1 (NU)	8.70	91.30
RABEP2 (C/CS/ES)	15.22	84.78
RANBP3 (C/NU)	8.17	91.83
RBBP7 (NU)	19.23	80.77
RBM8A(C/NU)	34.49	65.51
RCC2(NU/PM/CS)	12.27	87.73
RILPL1 (C/CS)	8.88	91.12
RPIA(C)	20.88	79.12
RPL4 (C/NU/ER/EC)	81.08	18.92
RRM2 (C)	19.37	80.63
RRM2B (C/NU)	13.50	86.50
RRP9 (NU)	83.78	16.22
S100A14 (C)	21.87	78.13
S100A8 (C/PM/EC/CS)	60.49	39.51
S100A9 (C/CS/PM/EC)	65.19	34.81
SDE2 (NU)	4.12	95.88
SELENBP1 (C/NU)	5.50	94.50
SERBP1 (C/NU)	19.84	80.16
SERPINA1 (ER/EC)	6.31	93.69
SERPINB1 (C/ES/LY/EC)	5.58	94.42
SERPINB3 (C)	67.93	32.07
SFN (C/NU/EC)	5.47	94.53
SH3BP5 (MT/VE)	0	100.00
SHTN1 (CS)	28.26	71.74
SKP1 (C/NU)	2.29	97.71
SLC9A3R (NU/PM)	7.80	92.20
SMAP1 (PM)	2.11	97.89
SNAP23 (PM)	25.24	74.76
SNAPIN (C/LY/GA/ES)	9.15	90.85
SOD1 (C/NU/MT)	14.65	85.35
SOD2 (MT)	10.13	89.87
SORBS3 (NU/CS)	3.98	96.02
SRSF10 (C/NU)	78.62	21.38
SRXN1 (C)	23.09	76.91
STRAP (C/NU)	13.52	86.48
TACC3 (C/CS)	11.77	88.23
TBCA (CS)	7.00	93.00

Table S8 (page 6)

External H_2O_2 (1 mM, 10 min)

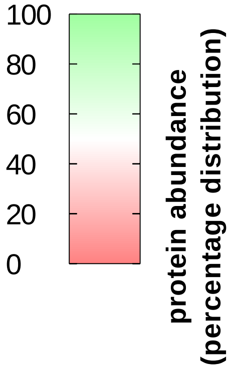

NaCl eluate	DTT eluate	
10.63	89.37	TCEAL4 (NU)
31.42	68.58	TGM1 (C/PM)
7.81	92.19	THOP1 (C)
16.26	83.74	TIPRL (C)
2.48	97.52	TK1 (C)
12.60	87.40	TPM1 (CS)
5.18	94.82	TPM3 (CS)
2.70	97.30	TPM4 (CS)
14.17	85.83	TSN (C/NU)
16.90	83.10	TSNAX (NU/GA/C)
0.96	99.04	TSR2 (???)
2.51	97.49	TTC1 (C/PO)
1.70	98.30	TTR (C/EC)
11.00	89.00	TUBA4A (CS)
39.39	60.61	TUBB (CS)
27.06	72.94	TWF1 (C/CS)
12.82	87.18	TWF2 (C/CS)
0	100.00	TXLNA (C/EC)
2.82	97.18	TXN (C/NU/EC)
37.68	62.32	TXNDC17 (C)
56.22	43.78	TXNL1 (C/NU)
3.08	96.92	UBAP2 (C/NU)
3.61	96.39	UBE2A (C/NU)
2.94	97.06	UBE2B (NU/PM)
7.96	92.04	UBE2G1 (C/EC)
3.62	96.38	UBE2H (C/NU)
2.64	97.36	UBE2I (C/NU)
10.10	89.90	UBE2K (C)
2.86	97.14	UBE2L3 (C/NU)
7.49	92.51	UBE2N (C/NU)
17.42	82.58	UBFD1 (???)
6.76	93.24	UQCRH (MT)
46.99	53.01	USP5 (C/NU/LY)
9.83	90.17	VBP1 (C/NU)
30.79	69.21	VCL (PM/CS)
37.99	62.01	VDAC2 (MT)
8.97	91.03	WBP2 (C/NU)
14.31	85.69	WDR44 (C/GA/ES)
66.21	33.79	WDR48 (C/NU/LY/ES)
15.71	84.29	WDR77 (C/NU)
0.22	99.78	XRCC4 (NU)
10.31	89.69	YWHAQ (C)
71.55	28.45	ZFAND5 (C)
0	100.00	ZFYVE16 (C/ES)

TABLE S9. Overview of S-carbamidomethylated cysteines. The S-carbamidomethylated cysteines identified in proteins that were at least 2.5-fold enriched in an (external or endogenous) H_2O_2-treated condition were compared to oxidative cysteine modifications reported in the iCysMod database and color coded: green, S-sulfenylation is has already been reported by others; blue, the iCysMod database lists other oxidative cysteine thiol modifications; red, the cysteine residue is not yet listed in the iCysMod database (based on the information available on January 7, 2022).

Protein	Cysteine position		
	External H_2O_2	Peroxisomal H_2O_2	Cytosolic H_2O_2
ACP1	13, 18		
ACP6	416		
ADH5	97, 100		
ADO	239		
AHCY	195		
AIFM1			317, 441
AK2	92		
AKAP12	1139, 1407, 1521		
ALDOA	339	339	73, 339
ANXA2	133		
ARFGAP1	351		
ASF1B	172, 178		
ASNS	158		
BAG3	151		
BLMH	326	326	
CASP14		24	
CAST	241, 328, 381, 408, 413, 661		
CBX3			177
CDK4	78	78	
CHCHD4	87		
CHMP5	20		
CHORDC1			59
CIAPIN1	249, 251, 274, 277, 285, 288		274, 277
CNN3	173		
COX17	23, 24, 26, 45		45
CPD			195
CPOX	319		
CRKL	44, 249		44
CTH	109		
CTSB	93, 105, 108		93, 105, 108
CTSD		329	
CTNN	112, 246		
DCTPP1	162		
DFFA	38		
DHPS	142		
DNAJB11			193, 196
DNPEP	48, 337, 457		
DPYSL2	439, 504		248

Protein	Cysteine position		
	External H_2O_2	Peroxisomal H_2O_2	Cytosolic H_2O_2
DPYSL3	39 (isoform LCRMP-4), 248, 448, 471		248
DSC1	196, 454	196, 255, 315, 454	315
DSG1	77, 253, 255, 669, 699	77, 127, 253, 255, 589, 669, 699	
DSP	174, 798, 1069, 1805, 2259	135, 798, 2259	
DYNLL1	56		
EIF3G	139		
EIF3I	76		
EIF5A	73		
ERH		28	
ERICH5	35		
ERP44		92, 318	92, 189, 301, 318
FABP5		43, 47, 67, 87, 120, 127	
FLG2	25, 329, 338, 346, 366	317, 329, 338, 346, 366	
FN3KRP	24		
GAPDH			152, 156, 247
GLOD4	41 (isoforms 2 and 3), 197		197
GLRX3	229		
GLRX5	67		
GPHN	212, 284, 419		
GRB2	32		
GRN	164, 165, 171, 215, 221, 222, 284, 290, 296, 297		
GSR	102, 107, 278, 377, 484	377	102, 107
HAT1	101		
HDGF	108		
HPRT1	66, 106, 206	106, 206	206
HSD17B10	58, 91, 214	58, 91	91
HSPA1B			306
HSPA4L	540		
HSPA8		17	17
HSPA9	66		
IMPDH2	468		
ISCA2	79		
ISCU	69, 95		
JMJD6	101		
JPT2	118		
JUP	341, 372, 457, 511, 563, 609	457, 511, 563	
LCP1	101		

Protein	Cysteine position		
	External H_2O_2	Peroxisomal H_2O_2	Cytosolic H_2O_2
LRRFIP1	644		
LYRM7	97		
MAP4	535		
MARCKSL1	134		
MCMBP	287		
MDH1	137, 154		
NAA10	194		
NASP	254, 708		254, 708
NDUFAB1	140		
NFU1	210		
OTUB1	23		
PAWR	173		
PBK	22, 70, 112, 300		
PCBP1	54, 109, 158, 194		
PCMT1	95	95	
PDIA3	85, 92		
PDLIM1	73		
PHAX	51		
PIN1	113		
PITHD1	14, 187		42, 187
PKP1			409
PLS3	104, 167	167	104
PM20D2	303		
PNPO	156		
PPCDC	7		
PPIA		62	
PPP1R11	60, 61, 62		
PRDX1	71, 83, 173	173	71, 83
PRDX2	172		
PRDX3	229	229	229
PRDX4	51	51	51
PRDX5	100, 204		
PRDX6	47, 91	47, 91	47
PSMA6	78		
PSMD9	59		
PSME3	92		
RANBP3	228, 244 (isoform 2), 249		
RBBP7	97		97
RCC2	209, 337		
RPL7A		199	
S100A14	74	74	
S100A7			47
S100A8	42		

Protein	Cysteine position		
	External H_2O_2	Peroxisomal H_2O_2	Cytosolic H_2O_2
SELENBP1	31, 57, 268		
SHTN1	565		
SKP1	160		
SMAP1	33, 36		
SNAP23	112		
SOD1	58		
SRXN1	99		
STRAP	152, 305		305
TACC3	20, 206, 242, 426		
TBCA	67		
TGM3		367	
TIPRL	14, 87		
TK1	66, 153, 156, 206, 230		
TMED7			48, 109
TPI1		218	
TPM3	170 (isoforms 2, 3, and 6), 226 (isoform 2, position 136 in isoform 7), 233 (isoforms 2 and 5, position 143 in isoform 7)		170 (isoforms 2, 3, and 6)
TSR2	114		
TTC1	28		
TWF2	67, 141		
TXN	73	73	32, 35
TXNDC17	43, 46		
TXNDC5	121, 128, 381		
TXNRD2	54, 86, 91		
UBE2I	43		
UBE2K	170		
UBE2L3	86		86
UBE2N	87		
UCHL1			90, 152
UQCRH	37		
VBP1	113		
WDR48	225		
YWHAQ	94, 134, 237		134
YWHAZ	94		
ZFAND5	76		
