# Supplementary material for: Peroxisome-Derived Hydrogen Peroxide Modulates the Sulfenylation Profiles of Key Redox Signaling Proteins in Flp-In T-REx 293 Cells
Source: Front Cell Dev Biol. 2022 Apr 26;10:888873. doi: 10.3389/fcell.2022.888873 (PMC9086853; doi:10.3389/fcell.2022.888873)
Supplement: Supplementary file 2 [file DataSheet1.PDF]

```
gaattcccaccatgggactccggcagtcacaacctacaaactggtgattaacgggaagaca
          M  G  T  P  A  V  T  T  Y  K  L  V  I  N  G  K  T
  ctcaaaggtgaaacgaccacaaaagcagttgatgcggaaactgccgaaaaggcatttaag
   L  K  G  E  T  T  T  K  A  V  D  A  E  T  A  E  K  A  F  K
  caatatgccaacgataatggggtggacggggtgtggacgtatgacgatgccacgaaaaca
   Q  Y  A  N  D  N  G  V  D  G  V  W  T  Y  D  D  A  T  K  T
  ttcaccgtgactgaagtgaataccccgccgttacaacttataaattggttattaacggc
   F  T  V  T  E  V  N  T  P  A  V  T  T  Y  K  L  V  I  N  G
  aaaacactcaaggggagaccaccactaaggcagtggatgcagagactgcagagaaggca
   K  T  L  K  G  E  T  T  T  K  A  V  D  A  E  T  A  E  K  A
  ttcaagcaatacgcgaatgataacggcgtcgacggggtttggacttatgatgacgccact
   F  K  Q  Y  A  N  D  N  G  V  D  G  V  W  T  Y  D  D  A  T
  aagacttttacggtcacagagatcgatgagaacctgtactttcaaggcggaagcatggac
   K  T  F  T  V  T  E  I  D  E  N  L  Y  F  Q  G  G  S  M  D
  gaaaagacgacagggtggcggggaggtcacgttgttgagggtttggcaggtgaacttgaa
   E  K  T  T  G  W  R  G  G  H  V  V  E  G  L  A  G  E  L  E
  cagctccgggcgaggctggaacaccatcctcaaggtcaacgagaacccggggggaggagga
   Q  L  R  A  R  L  E  H  H  P  Q  G  Q  R  E  P  G  G  G  G
  tcactggagattacatctctttacaagaaggctggaagcacaaatggatcaagcctgcag
   S  L  E  I  T  S  L  Y  K  K  A  G  S  T  N  G  S  S  L  Q
  aatgctgacaagattaataatggaaatgacaacgataatgacaatgatgtggtaccttca
   N  A  D  K  I  N  N  G  N  D  N  D  N  D  N  D  V  V  P  S
  aaggagggctcactgctccgctgtagcgagatttgggaccgaataacaactcatccgaaa
   K  E  G  S  L  L  R  C  S  E  I  W  D  R  I  T  T  H  P  K
  tactctgacattgatgtagatgggctcgcgtccgaattgatggccaaggccaaaacaagc
   Y  S  D  I  D  V  D  G  L  A  S  E  L  M  A  K  A  K  T  S
  gaacgaggagtcgtgataaacgcggaagatgtccagcttgcattgaataaacatatgaac
   E  R  G  V  V  I  N  A  E  D  V  Q  L  A  L  N  K  H  M  N
  tgagcggccgc
```

FIGURE S1. DNA and protein sequence of human codon-optimized IBD-SBP-YAP1C. The start and stop codons are indicated in bold. The restriction sites for EcoRI (gaattc) and NotI (gcggccgc) are shaded in grey. The IgG-binding domains (IBD), the streptavidin-binding domain (SBP), and the YAP1 C-terminal cysteine-rich domain (YAP1C) are shaded in yellow, blue, and black, respectively. The redox-active cysteine is shaded in green, and the amino acids shaded in red indicate positions where cysteines were replaced by alanine and threonine residues, respectively.

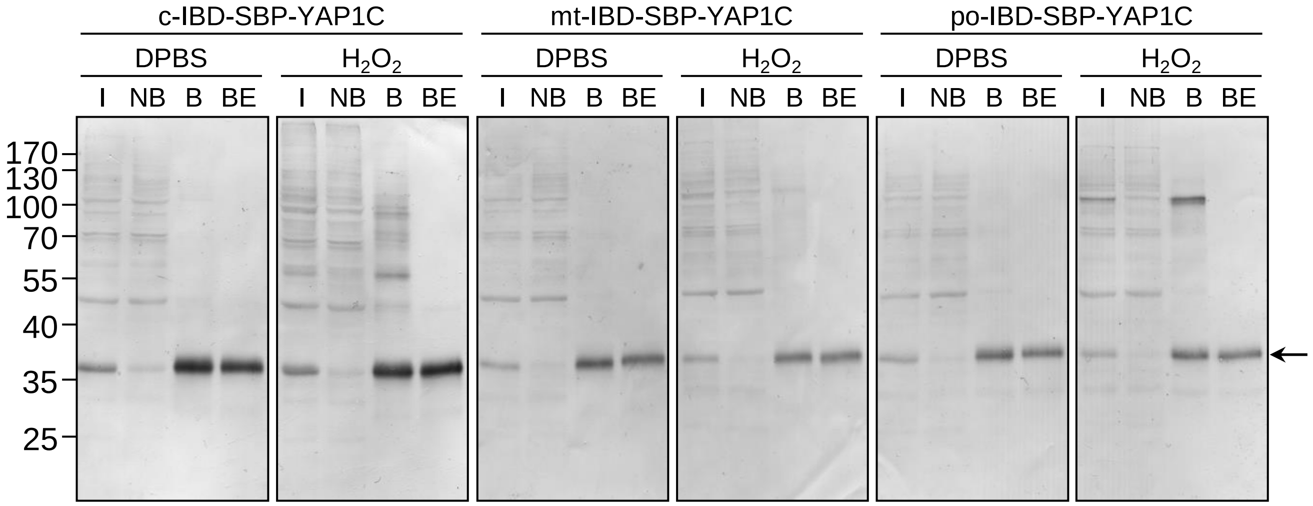

FIGURE S2. Differentially-localized IBD-SBP-YAP1C proteins form different complexes upon treatment of cells with external H_2O_2. Flp-In T-REx 293 cells expressing c-, mt-, or po-IBD-SBP-YAP1C were harvested and resuspended in DPBS containing 10 mM 3-AT, in combination (H_2O_2) or not (DPBS) with 1 mM H_2O_2. After 10 minutes, the cells were processed as detailed in the legend of Fig. 2B. The migration points of relevant molecular mass markers (expressed in kDa) are shown on the left. The arrow indicates the non-oxidatively modified YAP1C fusion proteins. I, input; NB, not bound to beads; B, bound to beads; BE, bound to beads after DTT elution. The DTT eluates were further processed for LC-MS/MS analysis (see Table S1).

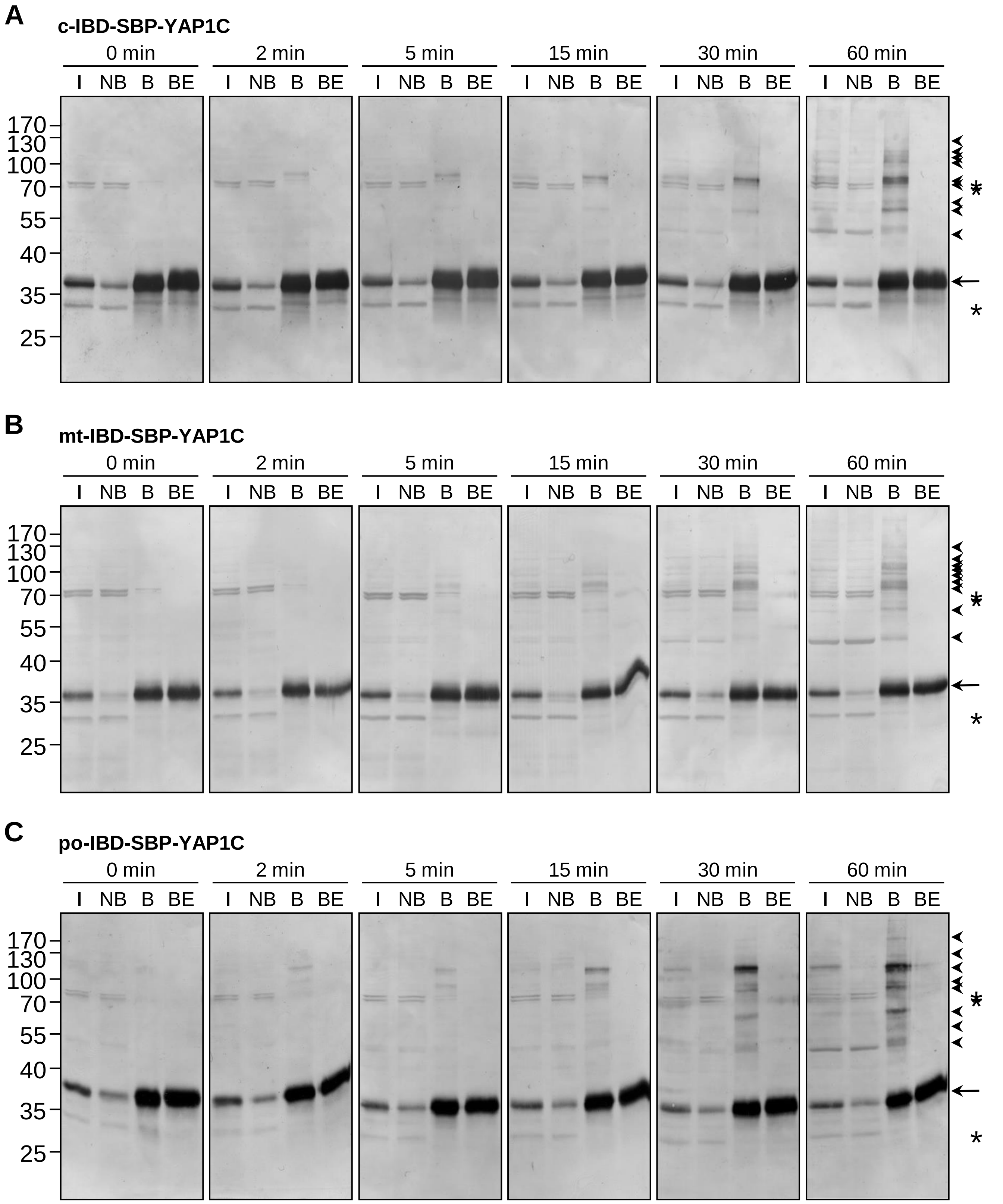

FIGURE S4. Formation kinetics of c-, mt-, and po-IBD-SBP-YAP1C complexes in response to peroxisomal H_2O_2 production. Flp-In T-REx 293 cells expressing po-DD-DAO and c-, mt-, or po-IBD-SBP-YAP1C (panels **A**, **B**, and **C**, respectively) were incubated in DPBS containing 10 mM 3-AT and 10 mM D-alanine. At selected time points (0, 2, 5, 15, 30, and 60 min), the cells were processed as detailed in the legend of Fig. 2B. The migration points of relevant molecular mass markers (expressed in kDa) are shown on the left. The arrow and arrowheads mark the non-modified and oxidatively modified IBD-SBP-YAP1C complexes, respectively. Non-specific bands are indicated with asterisks. I, input; NB, not bound to beads; B, bound to beads; BE, bound to beads after DTT elution. Note that the c-IBD-SBP-YAP1C experiment also included a condition without 3-AT, which is shown separately in Fig. S12.

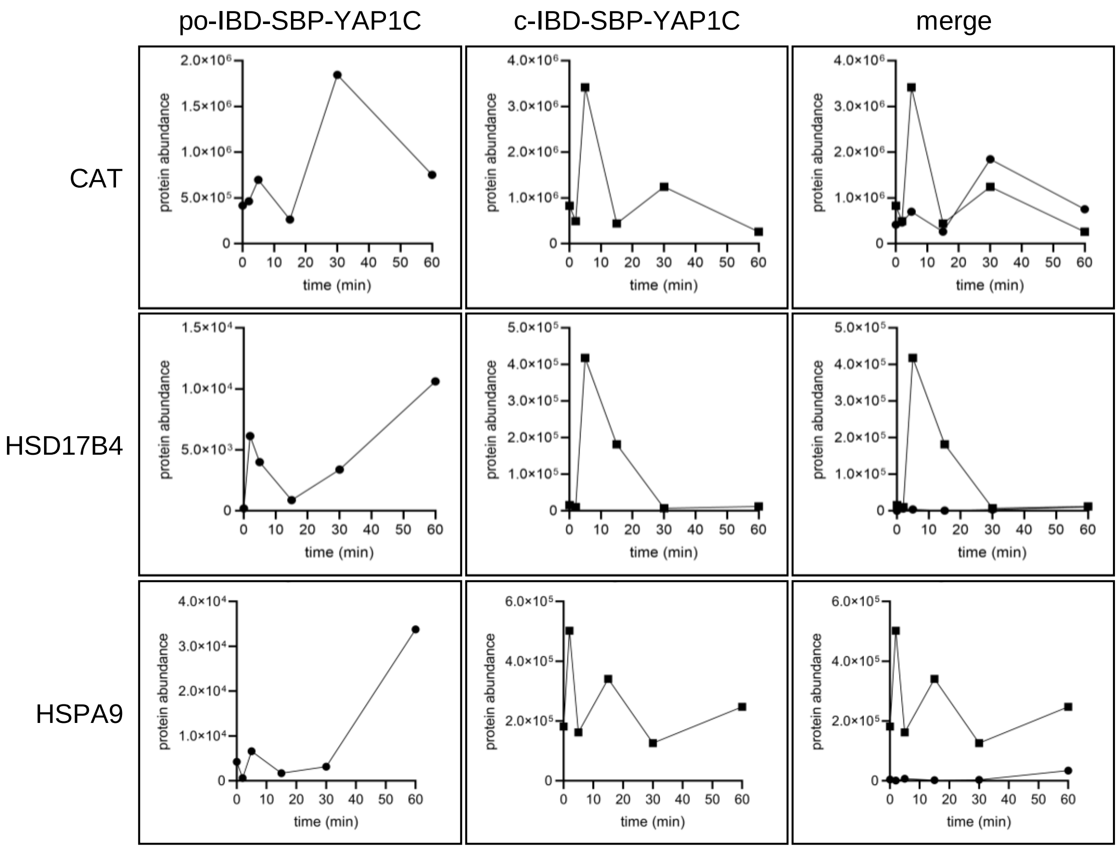

FIGURE S5. Sulfenome profiles of peroxisome-targeted po-IBD-SBP-YAP1C interactors in response to peroxisomal H_2O_2 production. Flp-In T-REx 293 cells expressing po-DD-DAO and po- or c-IBD-SBP-YAP1C were incubated in DPBS containing 10 mM 3-AT and 10 mM D-alanine. At selected time points (0, 2, 5, 15, 30, and 60 min), the cells were processed as detailed in the legend of Fig. 5. The raw protein abundances are plotted over time. Protein abundances are based on peptides that were commonly retrieved in the experiments shown, with the exception of HSD17B4 (for this protein, no common peptides were identified in the po-IBD-SBP-YAP1C and c-IBD-SBP-YAP1C samples).

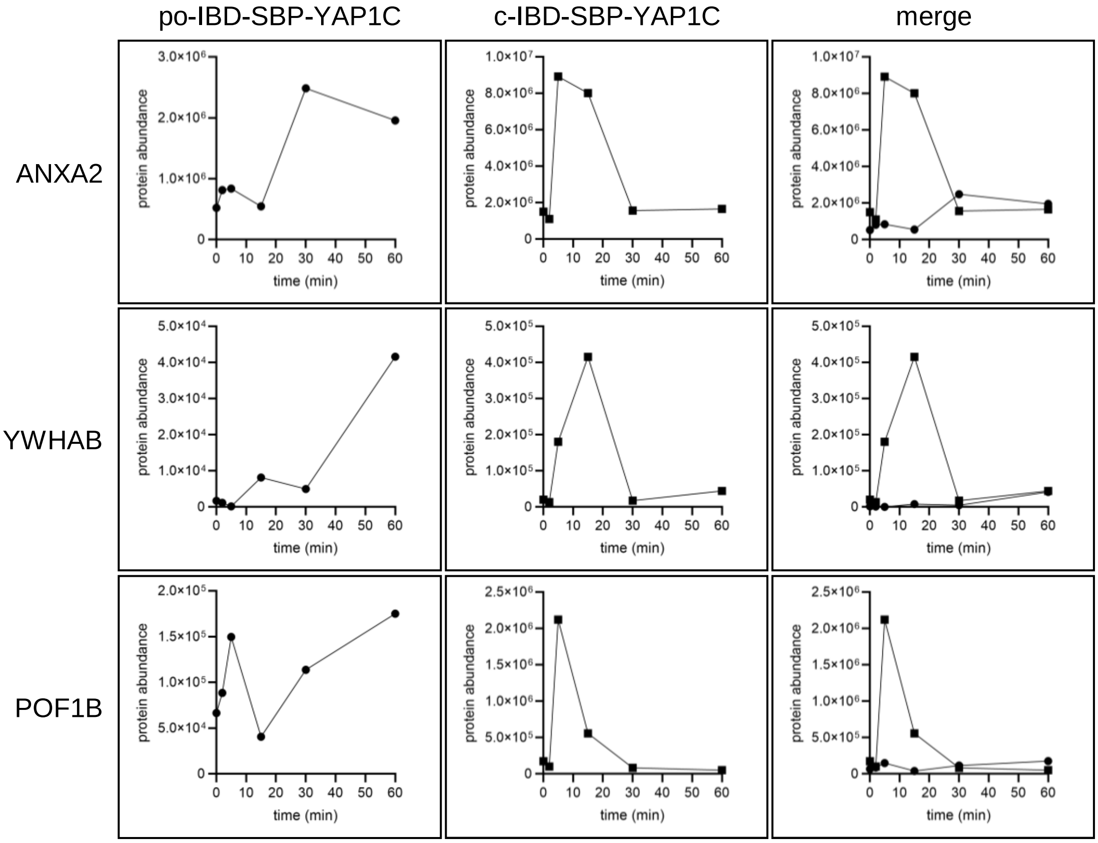

FIGURE S6. Sulfenome profiles of a selected set of cytosolically located po-IBD-SBP-YAP1C interactors in response to peroxisomal H_2O_2 production. Flp-In T-REx 293 cells expressing po-DD-DAO and po- or c-IBD-SBP-YAP1C were incubated in DPBS containing 10 mM 3-AT and 10 mM D-alanine. At selected time points (0, 2, 5, 15, 30, and 60 min), the cells were processed as detailed in the legend of Fig. 5. The raw protein abundances are plotted over time. Protein abundances are based on peptides that were commonly retrieved in the experiments shown.

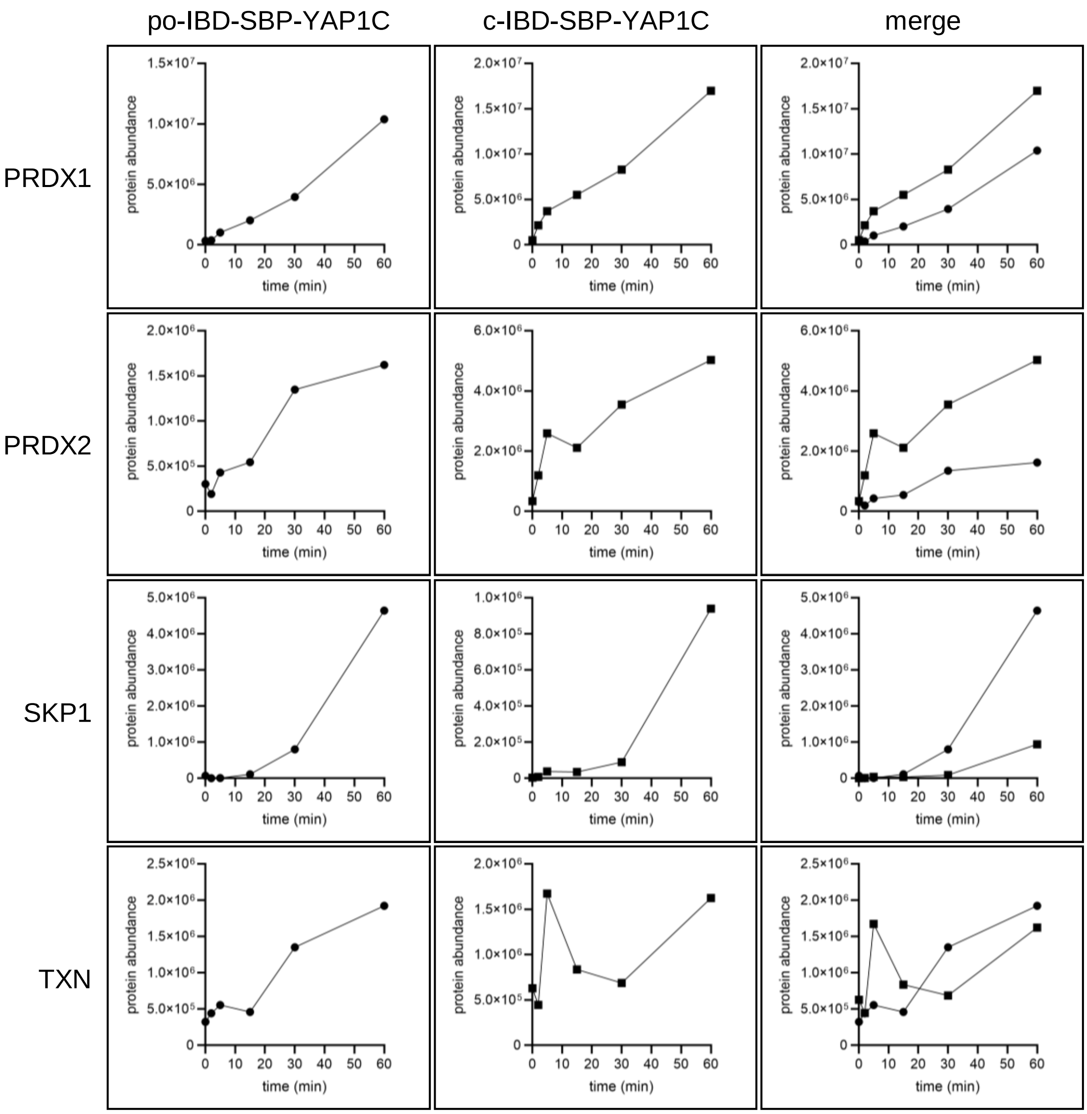

FIGURE S7. Sulfenome profiles of aberrantly behaving po-IBD-SBP-YAP1C interactors in response to peroxisomal H_2O_2 production. Flp-In T-REx 293 cells expressing po-DD-DAO and po- or c-IBD-SBP-YAP1C were incubated in DPBS containing 10 mM 3-AT and 10 mM D-alanine. At selected time points (0, 2, 5, 15, 30, and 60 min), the cells were processed as detailed in the legend of Fig. 5. The raw protein abundances are plotted over time. Protein abundances are based on peptides that were commonly retrieved in the experiments shown.

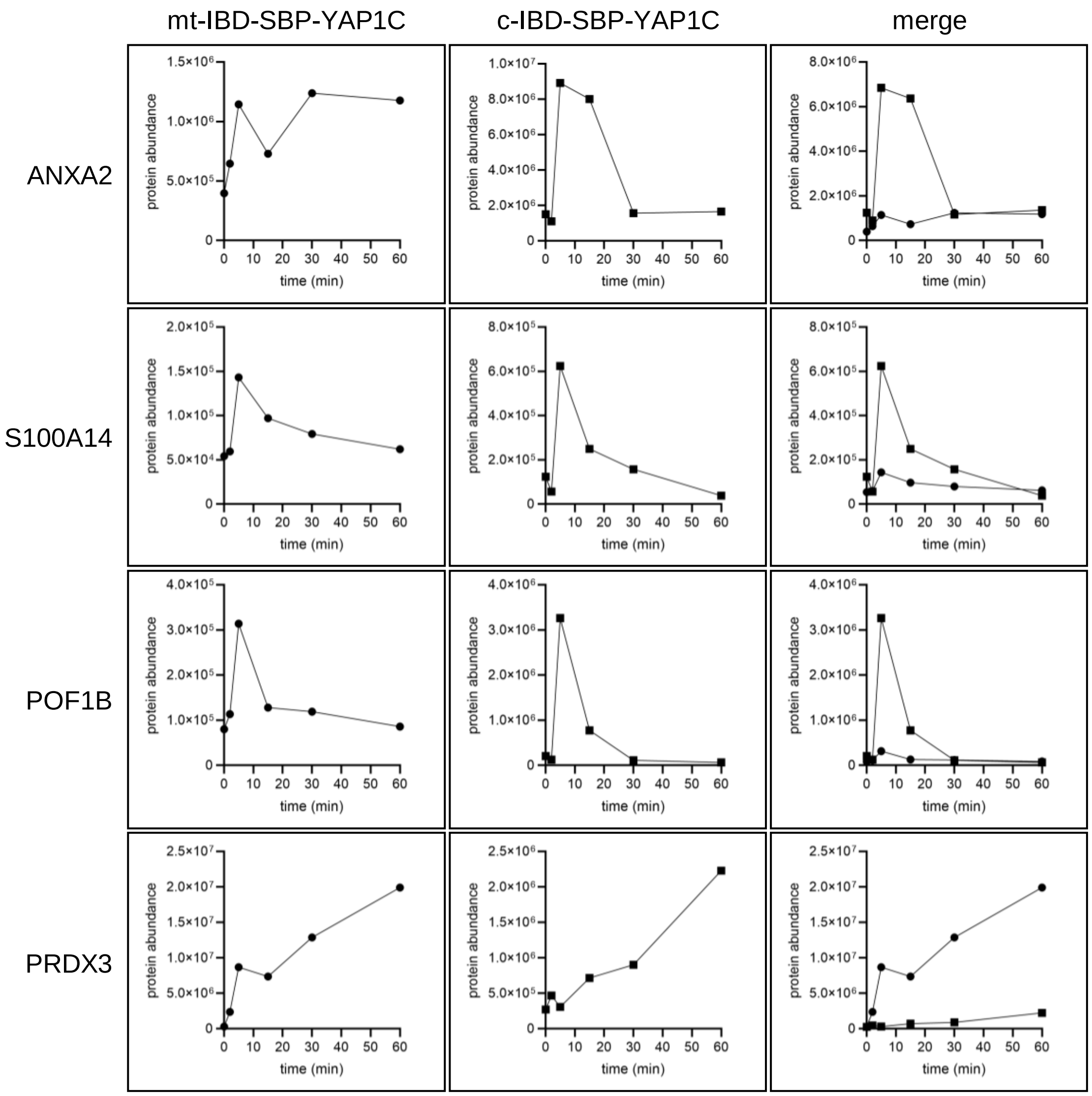

FIGURE S8. Sulfenome profiles of cytosolically located mt-IBD-SBP-YAPC1 interactors in response to peroxisomal H_2O_2 production. Flp-In T-REx 293 cells expressing po-DD-DAO and mt- or c-IBD-SBP-YAP1C were incubated in DPBS containing 10 mM 3-AT and 10 mM D-alanine. At selected time points (0, 2, 5, 15, 30, and 60 min), the cells were processed as detailed in the legend of Fig. 5. The raw protein abundances are plotted over time. Note that PRDX3 was included as reference profile of a typical mitochondrial protein. Protein abundances are based on peptides that were commonly retrieved in the experiments shown.

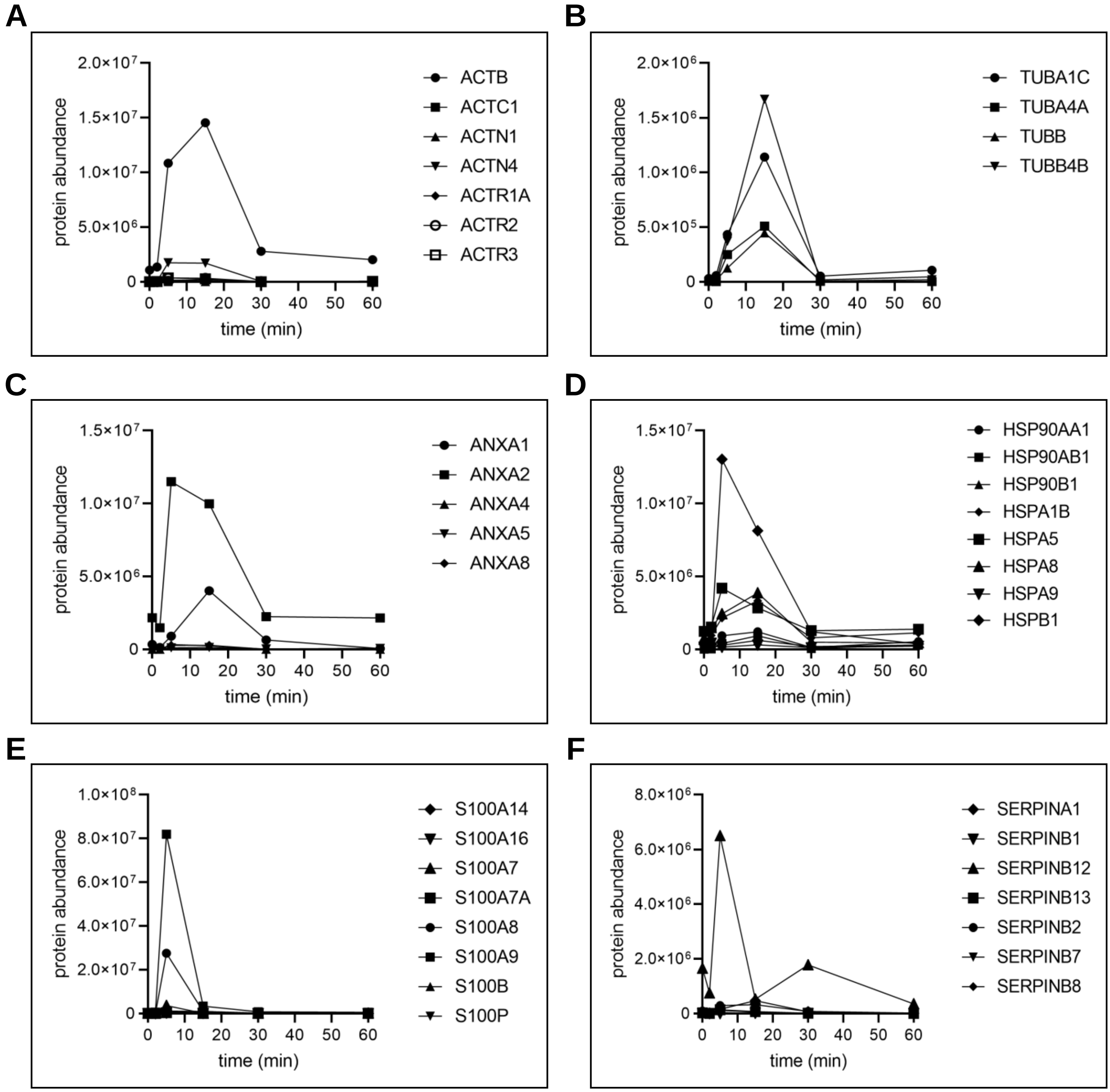

FIGURE S9. Sulfenome profiles of various protein families of c-IBD-SBP-YAPC1 interactors in response to peroxisomal H_2O_2 production. Flp-In T-REx 293 cells expressing po-DD-DAO and c-IBD-SBP-YAP1C were incubated in DPBS containing 10 mM 3-AT and 10 mM D-alanine. At selected time points (0, 2, 5, 15, 30, and 60 min), the cells were processed as detailed in the legend of Fig. 5. **(A)** Actin-related proteins, **(B)** tubulin-related proteins, **(C)** annexins, **(D)** protein chaperones, **(E)** S100 proteins, and **(F)** negative regulators of endopeptidase activity.

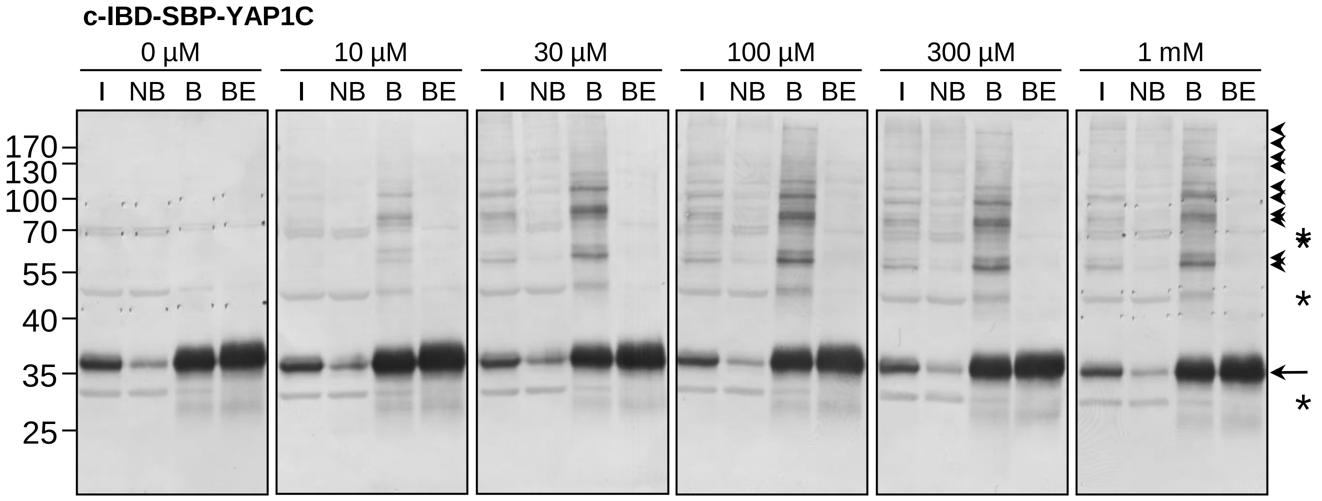

FIGURE S10. Formation of c-IBD-SBP-YAP1C complexes in response to different concentrations of external H_2O_2. Flp-In T-REx 293 cells expressing c-IBD-SBP-YAP1C were harvested and resuspended in DPBS containing 10 mM 3-AT and different concentrations of H_2O_2 (0, 10, 30, 100, 300, or 1000 µM). After 10 min, the cells were processed as detailed in the legend of Fig. 2B. The migration points of relevant molecular mass markers (expressed in kDa) are shown on the left. The arrow and arrowheads mark the non-modified and oxidatively modified c-YAP1C complexes, respectively. Non-specific bands are indicated with asterisks. I, input; NB, not bound to beads; B, bound to beads; BE, bound to beads after DTT elution.

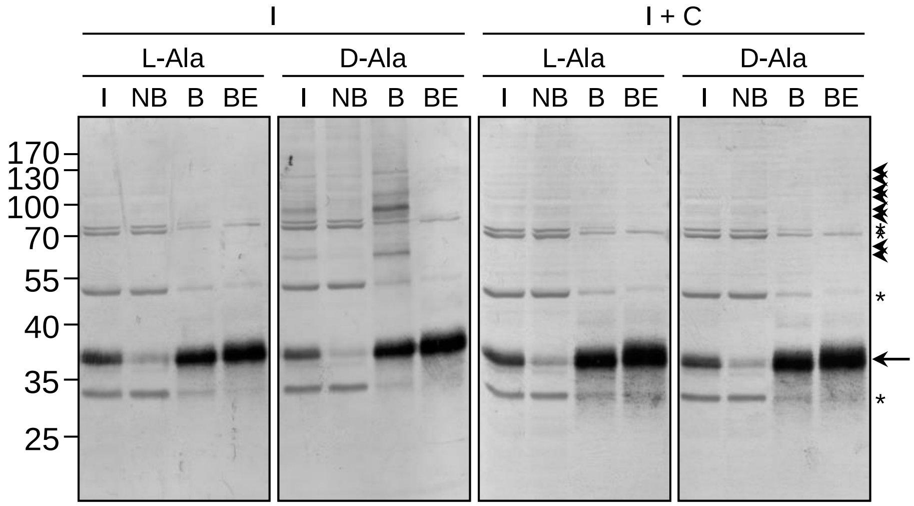

FIGURE S11. A 24-h chase period is sufficient to counteract c-DD-DAO activity-induced c-IBD-SBP-YAP1C complex formation. Flp-In T-REx 293 cells expressing c-DD-DAO and c-IBD-SBP-YAP1C were cultured in medium containing 1 µg/ml of DOX and 500 nM Shield1. After 3 days, the medium was replaced with medium containing (I, induction) or lacking (I+C, induction + chase) DOX/Shield1. 24-h later, the cells were incubated in DPBS containing 10 mM 3-AT and 10 mM L- or D-alanine. After 10 min, the cells were processed as detailed in the legend of Fig. 2B. The migration points of relevant molecular mass markers (expressed in kDa) are shown on the left. The arrow and arrowheads mark the non-modified and oxidatively modified c-IBD-SBP-YAP1C complexes, respectively. Non-specific bands are indicated with asterisks. I, input; NB, not bound to beads; B, bound to beads; BE, bound to beads after DTT elution.

A

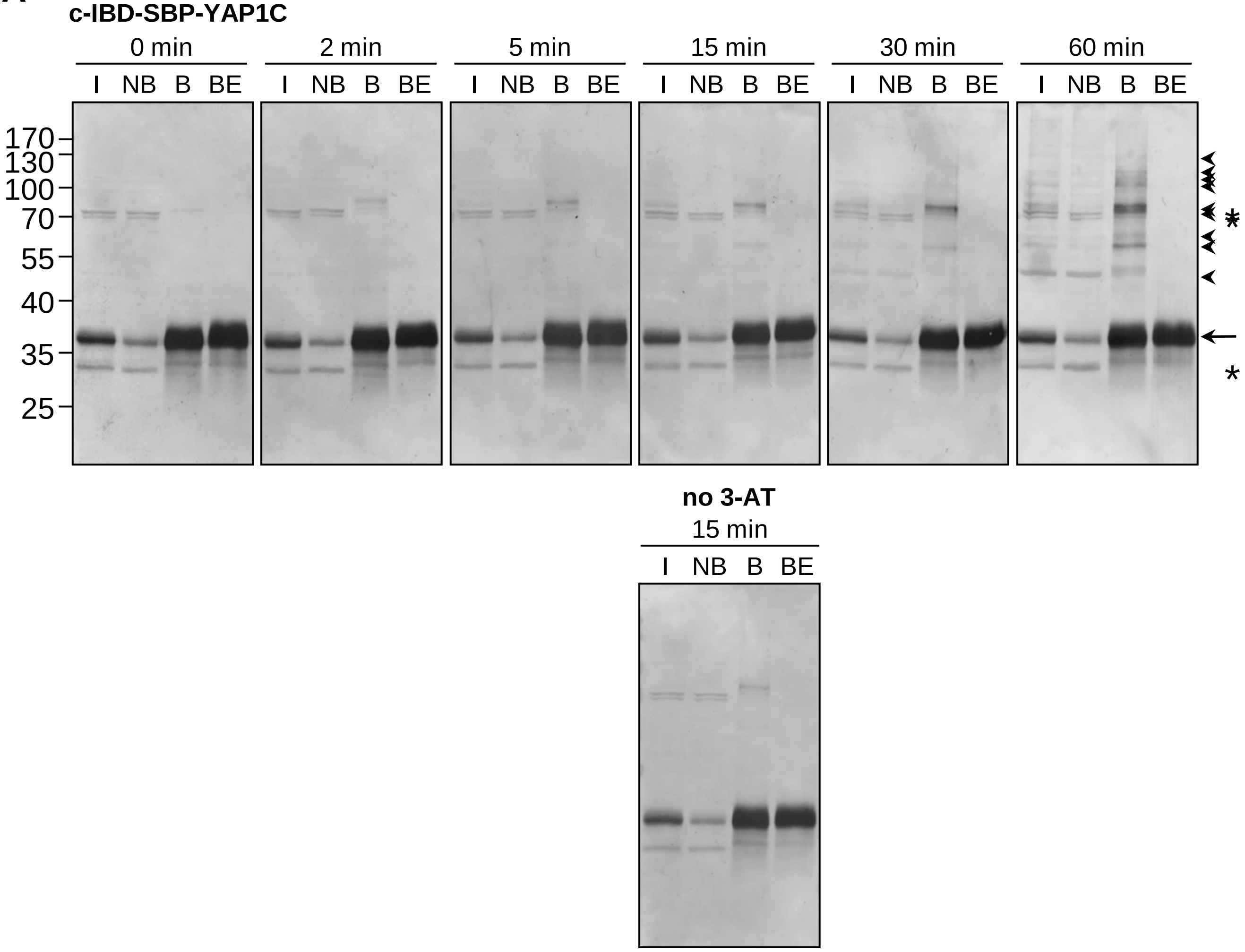

B

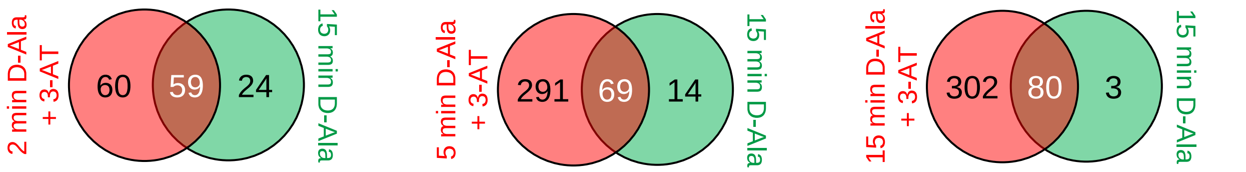

FIGURE S12. 3-AT increases the responsiveness of Flp-In T-REx 293 cells to peroxisome-derived H_2O_2. (A) For cell treatment and sample preparation, see legend to Fig. S4A. The migration points of relevant molecular mass markers (expressed in kDa) are shown on the left. The arrow and arrowheads mark the non-modified and oxidatively modified IBD-SBP-YAP1C complexes, respectively. Non-specific bands are indicated with asterisks. I, input; NB, not bound to beads; B, bound to beads; BE, bound to beads after DTT elution. **(B)** Venn diagrams comparing the number and overlap of c-IBD-SBP-YAP1C interactors in Flp-In T-REx 293 cells expressing po-DD-DAO and treated with 10 mM D-Ala for (i) 15 min in the absence of 3-AT and (ii) 2, 5, or 15 min in the presence of 10 mM 3-AT.
