# Supplementary figures and images for: Peroxisome-Derived Hydrogen Peroxide Modulates the Sulfenylation Profiles of Key Redox Signaling Proteins in Flp-In T-REx 293 Cells
Source: Front Cell Dev Biol. 2022 Apr 26;10:888873. doi: 10.3389/fcell.2022.888873 (PMC9086853; doi:10.3389/fcell.2022.888873)

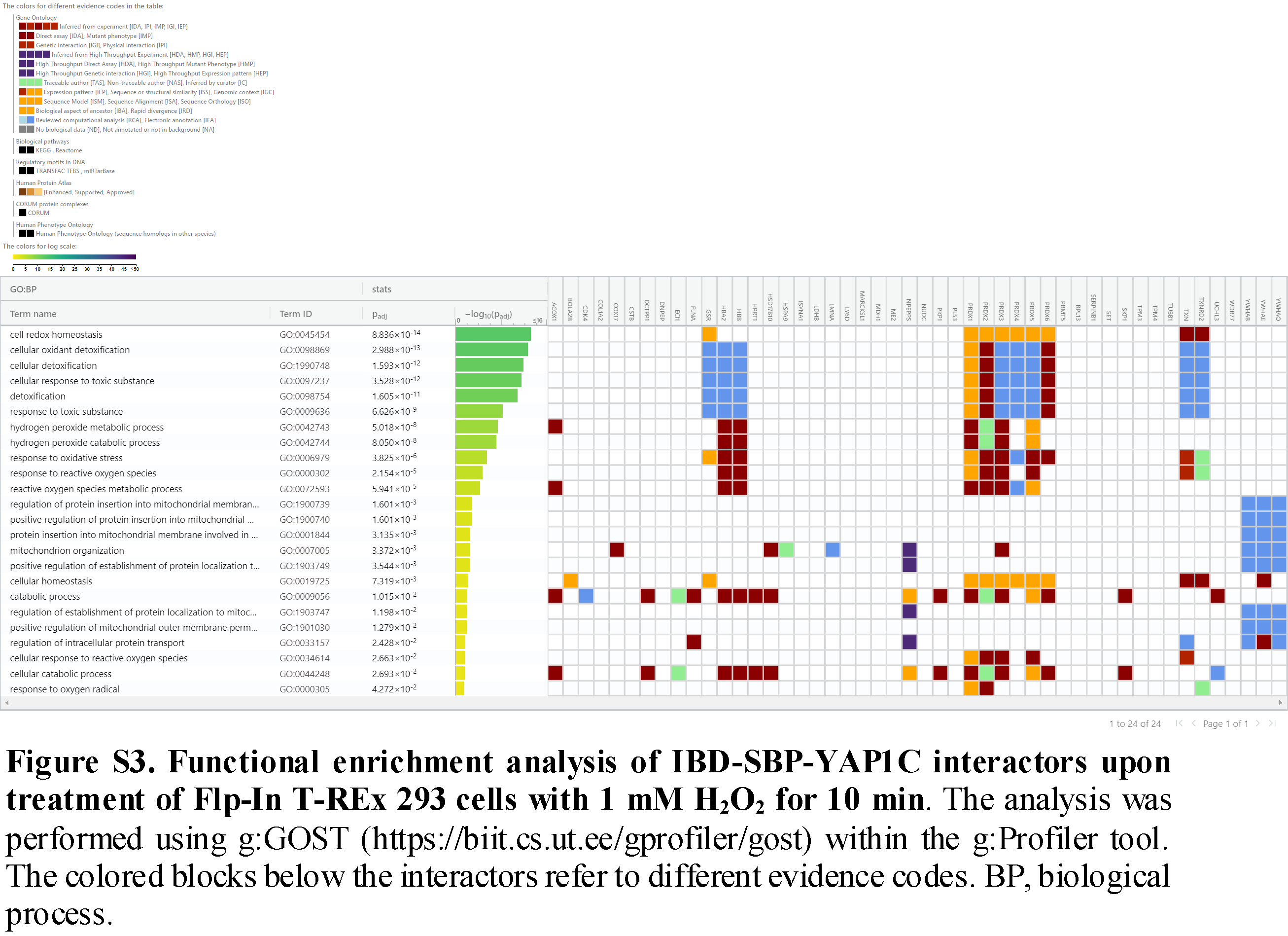

Supplement: Supplementary file 3 [file Image1.PNG]
